# A Sensitized Screen for Genes Promoting Invadopodia Function In Vivo: CDC-42 and Rab GDI-1 Direct Distinct Aspects of Invadopodia Formation

**DOI:** 10.1371/journal.pgen.1005786

**Published:** 2016-01-14

**Authors:** Lauren L. Lohmer, Matthew R. Clay, Kaleb M. Naegeli, Qiuyi Chi, Joshua W. Ziel, Elliott J. Hagedorn, Jieun E. Park, Ranjay Jayadev, David R. Sherwood

**Affiliations:** 1 Department of Biology, Duke University, Durham, North Carolina, United States of America; 2 Stem Cell Program and Division of Hematology/Oncology, Boston Children's Hospital and Dana Farber Cancer Institute, Howard Hughes Medical Institute, Harvard Stem Cell Institute, Harvard Medical School, Boston, Massachusetts, United States of America; University of California San Diego, UNITED STATES

## Abstract

Invadopodia are specialized membrane protrusions composed of F-actin, actin regulators, signaling proteins, and a dynamically trafficked invadopodial membrane that drive cell invasion through basement membrane (BM) barriers in development and cancer. Due to the challenges of studying invasion in vivo, mechanisms controlling invadopodia formation in their native environments remain poorly understood. We performed a sensitized genome-wide RNAi screen and identified 13 potential regulators of invadopodia during anchor cell (AC) invasion into the vulval epithelium in *C*. *elegans*. Confirming the specificity of this screen, we identified the Rho GTPase *cdc-42*, which mediates invadopodia formation in many cancer cell lines. Using live-cell imaging, we show that CDC-42 localizes to the AC-BM interface and is activated by an unidentified vulval signal(s) that induces invasion. CDC-42 is required for the invasive membrane localization of WSP-1 (N-WASP), a CDC-42 effector that promotes polymerization of F-actin. Loss of CDC-42 or WSP-1 resulted in fewer invadopodia and delayed BM breaching. We also characterized a novel invadopodia regulator, *gdi-1* (Rab GDP dissociation inhibitor), which mediates membrane trafficking. We show that GDI-1 functions in the AC to promote invadopodia formation. In the absence of GDI-1, the specialized invadopodial membrane was no longer trafficked normally to the invasive membrane, and instead was distributed to plasma membrane throughout the cell. Surprisingly, the pro-invasive signal(s) from the vulval cells also controls GDI-1 activity and invadopodial membrane trafficking. These studies represent the first in vivo screen for genes regulating invadopodia and demonstrate that invadopodia formation requires the integration of distinct cellular processes that are coordinated by an extracellular cue.

## Author Summary

During animal development specialized cells acquire the ability move and invade into other tissues to form complex organs and structures. Understanding this cellular behavior is important medically, as cancer cells can hijack the developmental program of invasion to metastasize throughout the body. One of the most formidable barriers invasive cells face is basement membrane–-a thin, dense, sheet-like assembly of proteins and carbohydrates that surrounds most tissues. Cells deploy small, protrusive, membrane associated structures called invadopodia (invasive feet) to breach basement membranes. How invadopodia are formed and controlled during invasion has been challenging to understand, as it is difficult to examine these dynamic structures in live animals. Using the nematode worm *Caenorhabditis elegans*, we have conducted the first large-scale screen to isolate genes that control invadopodia in live animals. Our screen isolated 13 genes and we confirmed two are key invadopodia regulators: the Rho GTPase CDC-42 that promotes F-actin polymerization at invadopodia to generate the force to breach basement membranes, and the Rab GDI-1 that promotes membrane addition at invadopodia that may allow invadopodia to extend through basement membranes. This work provides new insights into invadopodia construction and identifies potential novel targets for anti-metastasis therapies.

## Introduction

Basement membrane (BM) is a dense, highly cross-linked extracellular matrix that surrounds most tissues and acts as a barrier to migrating cells [1]. During development and immune function, specialized cells acquire the ability to cross BM barriers to facilitate cell movement into new tissues [2–4]. Misregulation of cell invasion also underlies the pathology of numerous human diseases, including cancer, where BM invasion initiates metastasis and is associated with poor prognosis [5,6]. Invadopodia are protrusive, F-actin rich, membrane associated structures that were identified over twenty years ago in vitro within transformed cells and highly metastatic cancer cell lines [7–9]. The formation and regulation of invadopodia have been examined extensively in cancer cell lines and tumor models because these structures are thought to facilitate tumor cell invasion across BM barriers [9–13]. Invadopodia are complex structures whose function requires the coordinated activity of actin regulators, signaling proteins, and membrane trafficking [8,14–17]. Due to the difficulty of examining the dynamic interactions between invasive cells, BM, and tissue being invaded in native physiological settings, the mechanisms that control invadopodia formation and activity in vivo remain largely unknown [18–20].

Anchor cell (AC) invasion into the vulval epithelium in *C*. *elegans* is a genetically and visually tractable model to dissect invadopodia formation and activity during BM invasion in vivo [20,21]. The AC is a specialized uterine cell that invades through the underlying BM to initiate contact with the vulval cells during uterine-vulval attachment. AC invasion is initiated by dynamic F-actin rich invadopodia that localize to the AC-BM interface (the invasive cell membrane) and breach the BM during a precise 15-minute window in the early-to-mid L3 larval stage. The netrin receptor UNC-40 (DCC) traffics to the breach site, where it promotes formation of a large protrusion that contacts the vulval tissue and clears a single large opening in the BM. Formation of the invasive protrusion also shuts down the production of invadopodia [22]. AC invadopodia are composed of F-actin and a number of actin regulators, including the ADF/cofilin ortholog UNC-60 and the Ena/VASP ortholog UNC-34. In addition, we have found that invadopodia are constructed from a specialized invadopodial membrane, enriched for the phospholipid PI(4,5)P_2_ and the membrane associated Rac GTPases MIG-2 and CED-10 [23]. The invadopodial membrane is dynamically recycled through the endolysosome during invadopodia formation and breakdown [23]. The mechanisms that control and coordinate the assembly of F-actin and the trafficking of the invadopodial membrane to invadopodia during their formation are poorly understood.

We performed a sensitized genome-wide RNAi screen and identified 13 putative regulators of invadopodia. Using quantitative live cell imaging, genetic analysis, and site of action studies, we have characterized two of these genes: the Rho GTPase *cdc-42*, which promotes invadopodia formation in a number of cancers; and the Rab GDP dissociation inhibitor *gdi-1*, a newly identified invadopodia regulator that mediates intracellular membrane trafficking. We find that both *cdc-42* and *gdi-1* are expressed and function in the AC to mediate distinct aspects of invadopodia formation. CDC-42 is localized and activated in puncta at the invasive cell membrane, where it promotes F-actin formation and initiates invadopodia generation through its effector WSP-1 (N-WASP). In contrast, GDI-1 is localized in the cytosol and regulates the proper targeting of the invadopodial membrane to the invasive cell membrane where invadopodia form. Strikingly, the activity of both GDI-1 and CDC-42 are controlled by the vulval cells, which secrete an unidentified cue(s) that stimulates AC invasion. Loss of the vulval cells led to a dramatic decrease in the rate of invadopodia formation and mislocalization of F-actin and the invadopodial membrane throughout the cell. Together, our findings have identified new regulators of invadopodia in vivo and show that an extrinsic signal(s) generated by the tissue being invaded coordinates distinct cellular aspects of invadopodia formation in the invading cell to promote BM breaching and invasion.

## Results

### A sensitized screen for genes regulating invadopodia and BM breaching

**Fig 1:**
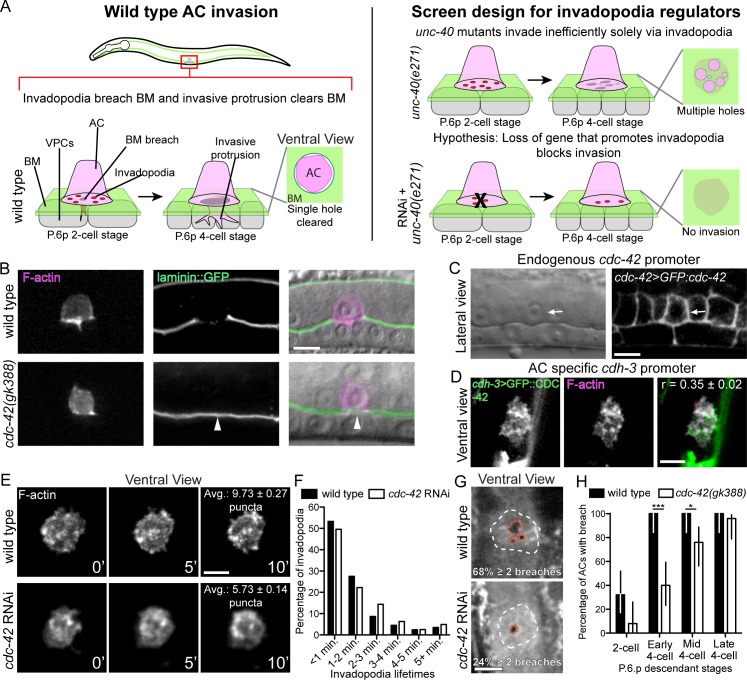
CDC-42 promotes invadopodia formation, BM breaching and invasion. (A) Diagram depicting anchor cell (AC) invasion through the basement membrane (BM) into the vulval epithelium in *C*. *elegans*. AC invasion is coordinated with the divisions of the P6.p vulval precursor cell descendants. At the P6.p two-cell stage, invadopodia dynamically form and turn over before breaching the BM. Following BM breach a single invasive protrusion forms, which displaces BM underneath the AC and creates a large hole in the BM. In *unc-40(e271)* mutants, the invasive protrusion does not form and the AC invades inefficiently by creating many BM breaches solely with invadopodia. In our genome-wide screen, enhancers of the *unc-40(e271)* invasion defect were identified as potential regulators of invadopodia. (B) Markers for F-actin (left; *cdh3>mCherry*::*moeABD*) and BM laminin (center; *lam-1>lam-1*::*GFP*) overlaid on DIC image (right). Top panels show wild type AC invasion and bottom panels show blocked AC invasion in a homozygous *cdc-42(gk388)* mutant (arrowhead marks intact BM) at the P6.p 4-cell stage. (C, D) Images of a functional GFP::CDC-42 GFP fusion protein acquired at the same developmental stage prior to invasion. (C) Lateral views of the GFP::CDC-42 reporter driven by the endogenous *cdc-42* promoter (*cdc-42>GFP*::*cdc-42*) show that *cdc-42* is expressed in the vulval and uterine cells, including the AC (arrow). (D) Ventral views showing that AC-specific GFP::CDC-42 (*cdh3>GFP*::*cdc-42*) is distributed in a punctate manner at the AC invasive surface and colocalizes with F-actin (r = Pearson’s correlation coefficient calculated from 5 animals). (E) A ventral time series shows dynamic formation of invadopodia (visualized with F-actin probe) in a wild type animal (top) and in an animal with reduced *cdc-42* (RNAi; bottom). The average number of invadopodia formed was decreased in animals treated with *cdc-42* RNAi (overlaid text reports average number of invadopodia per timepoint from 6 wild type animals and 10 *cdc-42* RNAi treated animals; p < 0.0001, Tukey’s multiple comparisons test). (F) The distribution of invadopodia lifetimes did not change in animals treated with *cdc-42* RNAi (n = 6 wild type and 10 *cdc-42* RNAi treated animals p = 0.178, Chi-square test) (G) Ventral views showing BM breaches mediated by invadopodia (white dashes outline AC footprint and orange dashes outline breaches). Wild type (top) animals typically breached through several small holes whereas animals with reduced *cdc-42* (RNAi; bottom) more frequently had a single breach (overlaid text reports percentage of animals with two or more holes; n = 17/25 wild type animals with > 2 breaches versus 6/25 animals treated with *cdc-42* RNAi; p<0.001, Fisher’s exact test. (H) BM breaching was delayed in *cdc-42(gk388)* mutants relative to wild type animals (P6.p vulval precursor cell development was used to calibrate time; n = 25 animals per stage, per genotype; *** p < 0001, * p < 0.05, Fisher’s exact test; bars represent 95% confidence intervals). Scale bars = 5 μm.

During AC cell invasion, F-actin rich, membrane associated invadopodia mediate the initial BM breach (Fig 1A). The netrin receptor UNC-40/DCC localizes to the BM breach site and directs formation of a large protrusion that widens the BM breach and shuts down additional invadopodia formation [22]. In the absence of UNC-40, BM invasion is driven solely by invadopodia, which create numerous BM breaches under the AC (Fig 1A). This inefficient form of BM clearing delays invasion in *unc-40* mutants [24,25]. To identify genes required for invadopodia BM breaching, we performed a sensitized genome-wide RNAi screen in an *unc-40*/DCC mutant background. Complete blocks in AC invasion disrupt uterine-vulval attachment and frequently result in an easily observed protruded vulva (Pvl) and egg-laying defective (Egl) phenotype. These phenotypes occur at low penetrance in *unc-40(e271)* mutants [24,25]. To identify regulators of invadopodia and BM breaching, we thus screened for genes whose RNAi-mediated knockdown enhanced the Pvl and Egl phenotypes in *unc-40(e271)* mutants. From the nearly 11,000 genes in the *C*. *elegans* ORF-RNAi library (approximately 55% of protein-coding genes in *C*. *elegans*) [26], we identified 722 genes that enhanced the *unc-40* Pvl and Egl phenotypes (S1 Table). To identify genes that directly control invadopodia and BM breaching, we performed a secondary screen of 150 candidate genes that are membrane associated, components of cell signaling pathways, or are predicted to be secreted (S2 Table; see Methods). Using differential interference contrast (DIC) microscopy to examine AC invasion, we found that RNAi-mediated knockdown of 13 genes significantly enhanced the *unc-40(e271)* invasion defect at the mid P6.p four-cell stage (the time when AC invasion is completed in wild-type animals; Tables 1 and S2). Confirming the rigor of this screen, we identified four genes previously characterized as regulators of AC invasion: *lin-3*, an EGF-like ligand required for specification of the vulval precursor cells, which generate a secreted cue(s) that promotes invasion [21]; *pat-4*, the *C*. *elegans* integrin-linked kinase [24]; *mig-6*, the *C*. *elegans* ortholog of the extracellular matrix protein Papilin [27]; and *cdc-42*, the *C*. *elegans* ortholog of the Cdc42 Rho GTPase [28]. Further, no known dedicated components of the UNC-40/DCC signaling pathway in the AC were identified [29,30].

**Table 1:** Genetic analysis of the roles of *cdc-42* and *gdi-1* during AC invasion.

Genotype/Treatment	Full invasion^±^	Partial invasion	No invasion	n =
**Secondary RNAi screen [human orthologs listed in brackets]**				
*unc-40(e271)* [Deleted in colon cancer (DCC)]	28%	17%	54%	150
*mig-6* [Papilin] RNAi; unc-40(e271)**	4%	7%	89%	150
*gdi-1* [Rab GDIβ] RNAi; unc-40(e271)**	5%	15%	80%	150
*gei-4* RNAi [LOC283658 portein]; unc-40(e271)*	6%	8%	86%	50
*cdc-42* RNAi [CDC42]; unc-40(e271)**	7%	11%	83%	150
*gpr-1* [n/a] RNAi; unc-40(e271)**	9%	13%	77%	150
*F09A5*.*4* [n/a] RNAi; unc-40(e271)**	9%	19%	71%	150
*mel-11* [n/a] RNAi; unc-40(e271)**	9%	24%	67%	150
*gpr-2* [n/a] RNAi; unc-40(e271)**	10%	18%	72%	150
*gtr-1* [n/a] RNAi; unc-40(e271)**	10%	19%	71%	150
*gsa-1* [GNAS] RNAi; unc-40(e271)**	11%	12%	77%	150
*pat-4* [Integrin-linked kinase] RNAi; unc-40(e271)*	12%	17%	71%	150
*Y70D2A*.*1* [GPR139] RNAi; unc-40(e271)*	12%	20%	68%	150
*lin-3* [n/a] RNAi; *unc-40(e271)*	no vulval precursor cells			
**Tissue-specific RNAi experiments**				
Uterine-specific RNAi control	92%	6%	2%	100
Uterine-specific *wsp-1* RNAi^a^	41%	27%	32%	100
Uterine-specific *gdi-1* RNAi^a^	44%	23%	33%	100
Vulval-specific RNAi control	98%	2%	0%	50
Vulval-specific *gdi-1* RNAi^b^	96%	4%	0%	45
**Rescue and Genetic interaction experiments**				
Wild type	100%	0%	0%	>100
*cdc-42(gk388)*^c^	66%	10%	24%	50
*cdc-42* RNAi^c^^,^^d^	72%	15%	14%	74
*cdc-42(gk388); cdc-42* RNAi^c^^,^^d^	68%	8%	24%	25
*cdc-42(gk388)*; *qyIs410[cdh-3>GFP*:*cdc-42]*^e^	92%	4%	4%	25
*wsp-1(gm324)*^c^	18%	28%	54%	50
*gdi-1* RNAi^c^	57%	13%	30%	30
*qyEx533 [cdh3>GFP*::*Cbrgdi-1]*; *gdi-1* RNAi^f^	91%	5%	4%	57
*gdi-1* RNAi; *cdc-42* RNAi^ff^	35%	35%	29%	31
*cdc-42(gk388)*; *gdi-1* RNAi^dd^	5%	18%	78%	40
*lin-3* RNAi	18%	13%	69%	100
*lin-3* RNAi; *cdc-42(gk388)*^g^	18%	12%	70%	100
*lin-3(n378)/lin-3(1059)*	21%	10%	69%	100
*gdi-1* RNAi; *lin-3(n378)/lin-3(1059)*^h^	20%	13%	67%	100

^±^Full invasion, partial invasion, and no invasion were defined by the degree to which the BM beneath the AC was cleared, as previously described [21].

Statistical comparisons of scoring (Fisher's exact test, normal invasion versus defective invasion):

*Compared with *unc-40(e271)*; p<0.001

**Compared with *unc-40(e271);* p<0.0001

^a^Compared with uterine-specific RNAi control; p<0.0001

^b^Compared with vulval-specific RNAi control; not significant

^c^Compared with wild type; p<0.0001

^d^Compared with *cdc-42(gk388);* not significant

^e^Compared with wild type; not significant; heterozygous *qyIs410* rescue

^dd^Compared with *cdc-42(gk388);* p<0.0001

^f^Compared with *gdi-1* RNAi; p<0.001

^ff^Compared with *gdi-1* RNAi; not significant

^g^Compared with *lin-3* RNAi; not significant

^h^Compared with *lin-3(n378)/lin-3(1059);* not significant

### CDC-42 localizes to the invasive membrane and promotes invadopodia formation

RNAi knockdown of the Rho GTPase *cdc-42* showed one of the strongest enhancements of the *unc-40(e271)* AC invasion defect (Table 1). Further, vertebrate Cdc42 has been implicated in stimulating invadopodia in cancer cell lines, but its function in regulating invadopodia in vivo is unknown [31,32]. We thus decided to examine the role of CDC-42 during AC invasion. We confirmed that homozygous *cdc-42(gk388)* mutants showed a penetrant disruption in AC invasion using DIC optics (34% defect; see Table 1). Further, we examined a functional GFP fusion of the major BM component laminin (laminin::GFP), which revealed that many *cdc-42(gk388)* mutants failed to breach the BM at the normal time (Fig 1B; 6/25 *cdc-42(gk388)* animals had no BM breach at the mid P6.p four-cell stage whereas 25/25 of wild type animals breached the BM; p < 0.001, Fisher’s exact test). We have recently shown that selective removal of the CDC-42 protein from the AC disrupts invasion to a degree similar to *cdc-42(gk388)* mutant animals [33]. In addition, RNAi targeting of *cdc-42* blocked AC invasion and did not further enhance the *cdc-42(gk388)* mutant invasion phenotype (Table 1). These observations offer compelling evidence that the *cdc-42(gk388)* mutant has a significant, and likely complete loss of *cdc-42* function in the AC. Based on experiments using a cell-specific protein degradation system, we previously proposed that CDC-42 functions in the AC to promote invasion [33]. Confirming that CDC-42 promotes invasion cell-autonomously, we found that AC-specific expression of a GFP-tagged CDC-42 (*cdh-3>GFP*::*cdc-42*) in *cdc-42* null animals fully rescued AC invasion in homozygous *cdc-42(gk388)* worms (Table 1).

As we previously reported, a rescuing *cdc-42* translational reporter (*cdc-42>GFP*::*cdc-42*) is expressed broadly in the developing uterine and vulval cells, including the AC (Fig 1C) [33]. When GFP::CDC-42 was expressed under its endogenous promoter, the signal from the neighboring uterine and vulval cells made it difficult to assess the subcellular distribution of GFP::CDC-42 in the AC (see Fig 1C). Therefore we examined AC-expressed GFP::CDC-42 (*cdh3>GFP*::*cdc-42*) at the same development stage. Ventral views revealed that GFP::CDC-42 was localized in punctate structures at the AC-BM interface prior to invasion (Fig 1D). Indicating that CDC-42 localizes to AC invadopodia, the GFP::CDC-42 puncta colocalized with F-actin, a marker for invadopodia (Fig 1D) [22].

We next wanted to determine if CDC-42 regulates invadopodia formation, dynamics, or function. As *cdc-42* mutants are sterile and lethal [34], we knocked down *cdc-42* using RNAi beginning at the early L1 larval stage, which resulted in an invasion defect similar to *cdc-42* null mutants from heterozygous mothers (Table 1). Quantitative live-cell imaging revealed that invadopodia size and lifetime were not affected by loss of *cdc-42*; however, the rate of invadopodia formation was nearly cut in half (1.72 per minute compared to 2.75 per minute in wild type) and there were fewer invadopodia (Table 2; Fig 1E and 1F; S1 and S2 Movies). The F-actin puncta that did form after *cdc-42* knockdown colocalized with the invadopodia components UNC-34 (Ena/VASP orthologue) and the phospholipid (PI(4,5)P_2_) [22] (S1 Fig). These observations suggest that CDC-42 stimulates invadopodia construction and that loss of *cdc-42* significantly reduces, but does not appear to eliminate invadopodia formation.

**Table 2:** Invadopodia dynamics after loss of *cdc-42*, *gdi-1* and vulval cells.

	Wild type	*cdc-42* RNAi	*gdi-1* RNAi	*cdc-42; gdi-1* RNAi	Vulvaless
**Number**	9.73 ± 0.27	5.73 ± 0.14^a^^,^^aa^	4.19 ± 0.18^a^^,^^b^^,^^c^	5.81 ± 0.24	5.23 ± 0.19
**Volume** (μm^3^)	0.76 ± 0.02	0.69 ± 0.02^d^^,^^dd^^,^^f^	1.07 ± 0.04^d^^,^^e^^,^^f^	0.66 ± 0.03	1.25 ± 0.06
**Rate of formation** (number/minute)	2.75 ± 0.36	1.72 ± 0.18^g^^,^^h^	1.60 ± 0.27^g^	1.46 ± 0.31	0.86 ± 0.17

^a^Tukey’s multiple comparison test including wild type, *cdc-42* RNAi, and *gdi-1* RNAi; compared to wild type p < 0.001

^aa^Tukey’s multiple comparison test including wild type, *cdc-42* RNAi, and *gdi-1* RNAi; compared to *gdi-1* RNAi p < 0.001

^b^Tukey’s multiple comparison test including *cdc-42* RNAi, *gdi-1* RNAi, and *cdc-42;gdi-1* RNAi; compared to *cdc-42;gdi-1* RNAi p < 0.001

^c^Tukey’s multiple comparison test including *cdc-42* RNAi, *gdi-1* RNAi, and vulvaless; compared to vulvaless RNAi p < 0.001

^d^Bonferroni’s’s multiple comparison test including wild type, *cdc-42* RNAi, and *gdi-1* RNAi; compared to wild type RNAi p < 0.001

^dd^Bonferroni’s multiple comparison test including wild type, *cdc-42* RNAi, and *gdi-1* RNAi; compared to *gdi-1* RNAi p < 0.001

^e^Bonferroni’s multiple comparison test including *cdc-42* RNAi, *gdi-1* RNAi, and *cdc-42;gdi-1* RNAi; compared to *cdc-42;gdi-1* RNAi p < 0.001

^f^Bonferroni’s multiple comparison test including *cdc-42* RNAi, *gdi-1* RNAi, and vulvaless; compared to vulvaless RNAi p < 0.01

^g^Tukey’s multiple comparison test including wild type, *cdc-42* RNAi, and *gdi-1* RNAi; compared to wild type p < 0.05

^h^Tukey’s multiple comparison test including *cdc-42* RNAi, *gdi-1* RNAi, and vulvaless; compared to vulvaless RNAi p < 0.01

### Loss of CDC-42 results in delayed and fewer BM breaches

We next wanted to determine the effects of decreased invadopodia due to loss of *cdc-42* on the ability of the AC to breach BM. AC invasion is tightly coordinated with the divisions of the underlying vulval precursor cell P6.p. The P6.p initiates division in the early-to-mid L3 larval stage (P6.p two-cell stage), the P6.p daughters divide again in the mid-L3 larval stage (P6.p four-cell stage), followed by a last division cycle in early L4 stage (P6.p eight-cell stage) [21]. AC invadopodia first breach the BM in a 15-minute window during the mid-L3 stage coinciding with the late P6.p two-cell stage and early P6.p four-cell stage [22]. We carefully examined BM breaching using ventral views of the AC-BM interface and found that loss of *cdc-42* delayed BM breaching by approximately one hour (Fig 1H). Furthermore, we observed fewer invadopodia mediated BM breaching events after loss of *cdc-42*. In wild type animals over 65% of animals had two or more BM breaches, whereas less than 25% of animals had multiple BM breaches after loss of *cdc-42* (Fig 1G). Taken together, these data suggest that loss of *cdc-42* reduces the number of invadopodia formed, resulting in fewer, and delayed invadopodia-mediated BM breaching events.

### WSP-1(N-WASP) functions downstream of CDC-42 in the AC

The molecular mechanism by which vertebrate Cdc42 stimulates invadopodia formation in cancer cell lines is poorly understood [35]. Depletion of the actin regulator N-WASP, a direct effector of Cdc42 [36], and depletion of Cdc42 have similar effects on invadopodia formation in cultured rat mammary MTln3 adenocarcinoma cells [32,37]. These results suggest that N-WASP may act downstream of Cdc42 to direct invadopodia formation in mammary carcinoma cells. The gene *wsp-1* encodes the sole *C*. *elegans* WASP-like protein [38]. A deletion mutant of *wsp-1* (*wsp-1(gm324)*) results in a loss of detectable protein and is thought to be a null or strong loss of function allele. Despite the severe disruption in *wsp-1*, *wsp-1(gm324)* animals are homozygous viable [38].

**Fig 2:**
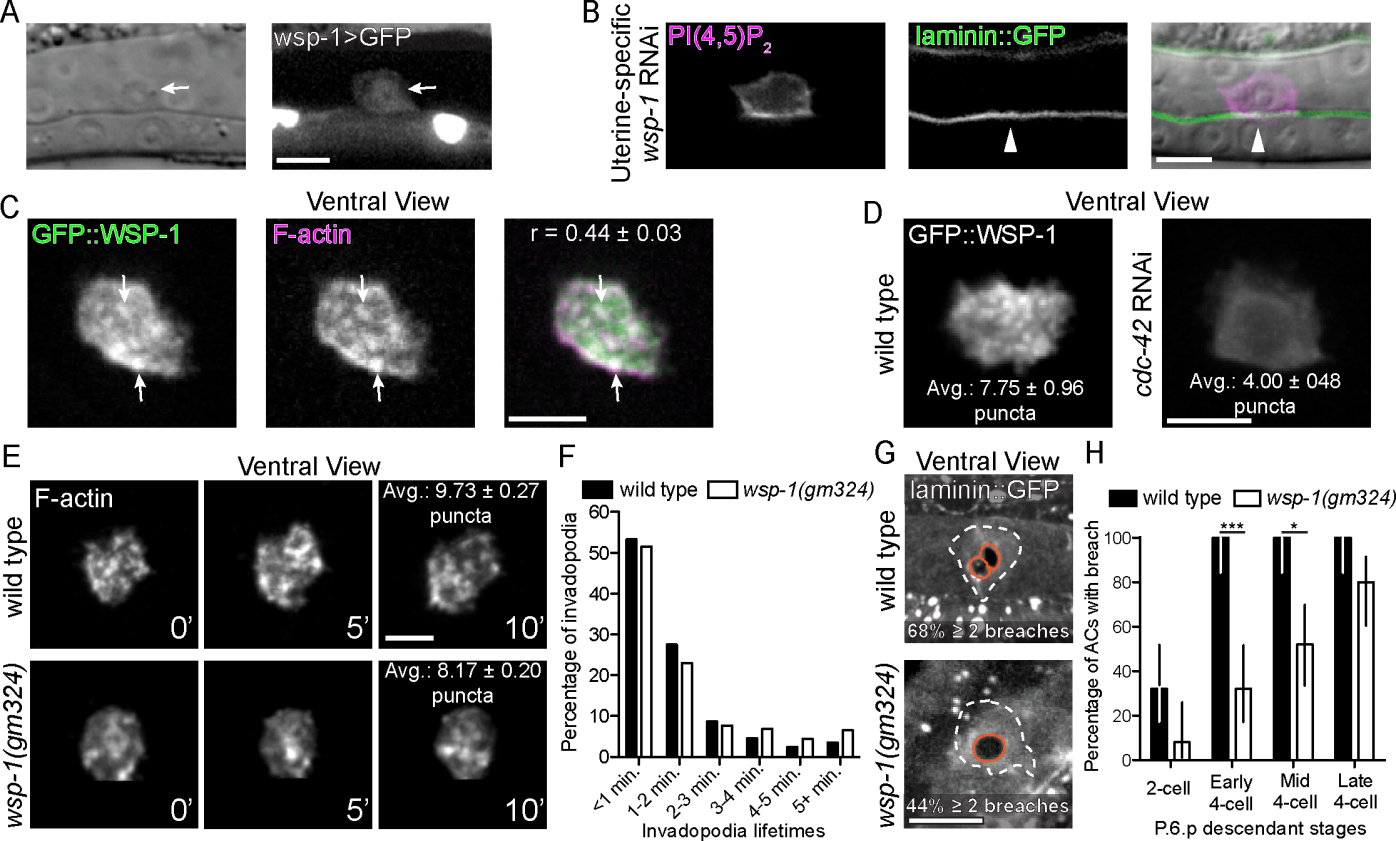
WSP-1 acts downstream of CDC-42 during invadopodia. (A) A transcriptional reporter (*wsp-1>GFP*) revealed *wsp-1* expression in the AC (arrow). (B) Markers for the phospholipid PI(4,5)P_2_ (left; *cdh3> mCherry*::*PLCδ*^*PH*^) and laminin (center; *lam-1>lam-1*::*GFP*) are overlaid on DIC image (right). Uterine-specific RNAi against *wsp-1* blocked AC invasion (arrowhead marks intact BM). (C) Ventral views show that prior to invasion GFP::WSP-1 (*cdh3>GFP*::*wsp-1*) is distributed in a punctate manner within the AC and colocalizes with F-actin (*cdh3>mCherry*:*moeABD*) (r = Pearson’s correlation coefficient calculated from 12 animals). (D) Knockdown of *cdc-42* (RNAi) decreased the number of GFP::WSP-1 puncta at the invasive membrane (overlaid text reports average number of puncta from 9 animals; p < 0.01, Student’s t-test). (E) A ventral time series shows fewer invadopodia (visualized with F-actin) formed in animals lacking *wsp-1* (*wsp-1(gm324)*; bottom) relative to wild type (top) (overlaid text reports averages number of invadopodia number per timepoint from 6 wild type animals and 6 *wsp-1(gm324)* animals; p < 0.0001, Student’s t-test). (F) The distribution of invadopodia lifetimes did not change in animals treated with *wsp-1* RNAi (n = 6 wild type and 6 *wsp-1* RNAi treated animals; p = 0.252, Chi-square test) (G) Ventral views showing BM breaches (white dashes outline AC footprint and orange dashes outline breaches). *wsp-1(gm324)* mutants (bottom) show a single BM breach more frequently relative to wild type (overlaid text reporting percentage of animals with two or more holes; n = 17/25 wild type animals with > 2 breaches versus 11/25 *wsp-1(gm324)* animals; p<0.01, Fisher’s exact test). (H) BM breaching was delayed in *wsp-1(gm324)* mutants (P6.p vulval precursor cell descendant development was used to calibrate timing; n = 25 animals per stage, per genotype; *** p < 0.0001, * p < 0.05, Fisher’s exact test; bars represent 95% confidence intervals). Scale bars = 5 μm.

We examined AC invasion by DIC in *wsp-1(gm324)* mutants and found a highly penetrant delay or block in AC invasion at the mid P6.p four-cell stage (Table 1). Suggesting a possible cell autonomous role in AC invasion, a transcriptional reporter for *wsp-1* (*wsp-1>GFP*) was expressed in the AC (Fig 2A). Uterine-specific RNAi-mediated knockdown of *wsp-1* resulted in AC invasion defects where the BM (viewed with laminin::GFP) was not breached (n = 12/25 animals with no BM breach at the mid P6.p four-cell stage; Fig 2B, Table 1). Because uterine cells neighboring the AC do not contribute to invasion [21], an AC invasion defect in this background indicates that *wsp-1* functions in the AC to promote invasion.

We next determined the localization of an AC-specific, functional GFP-tagged WSP-1 translational fusion protein (*cdh-3>GFP*::*wsp-1*). Similar to GFP::CDC-42, GFP::WSP-1 was found in punctate structures at the ACs invasive cell membrane that colocalized with F-actin puncta, indicating that WSP-1 localizes to AC invadopodia (Fig 2C). As N-WASP is localized in part by directly binding active Cdc42 (GTP-bound) [39], we next examined if loss of CDC-42 altered WSP-1 localization. The number of GFP::WSP-1 puncta at the invasive cell membrane was significantly reduced after RNAi-mediated knockdown of *cdc-42* (Fig 2D), indicating that WSP-1 localization is CDC-42 dependent. Furthermore, we found that similar to loss of *cdc-42*, *wsp-1* mutants had normal invadopodia lifetimes but significantly fewer invadopodia, delayed BM breaching, and fewer BM breaching events relative to wild type animals (Fig 2E–2H). Together, these results suggest that *wsp-1* functions downstream of *cdc-42* to promote invadopodia formation in the AC.

### The vulval cells are required to activate CDC-42 at the invasive membrane

**Fig 3:**
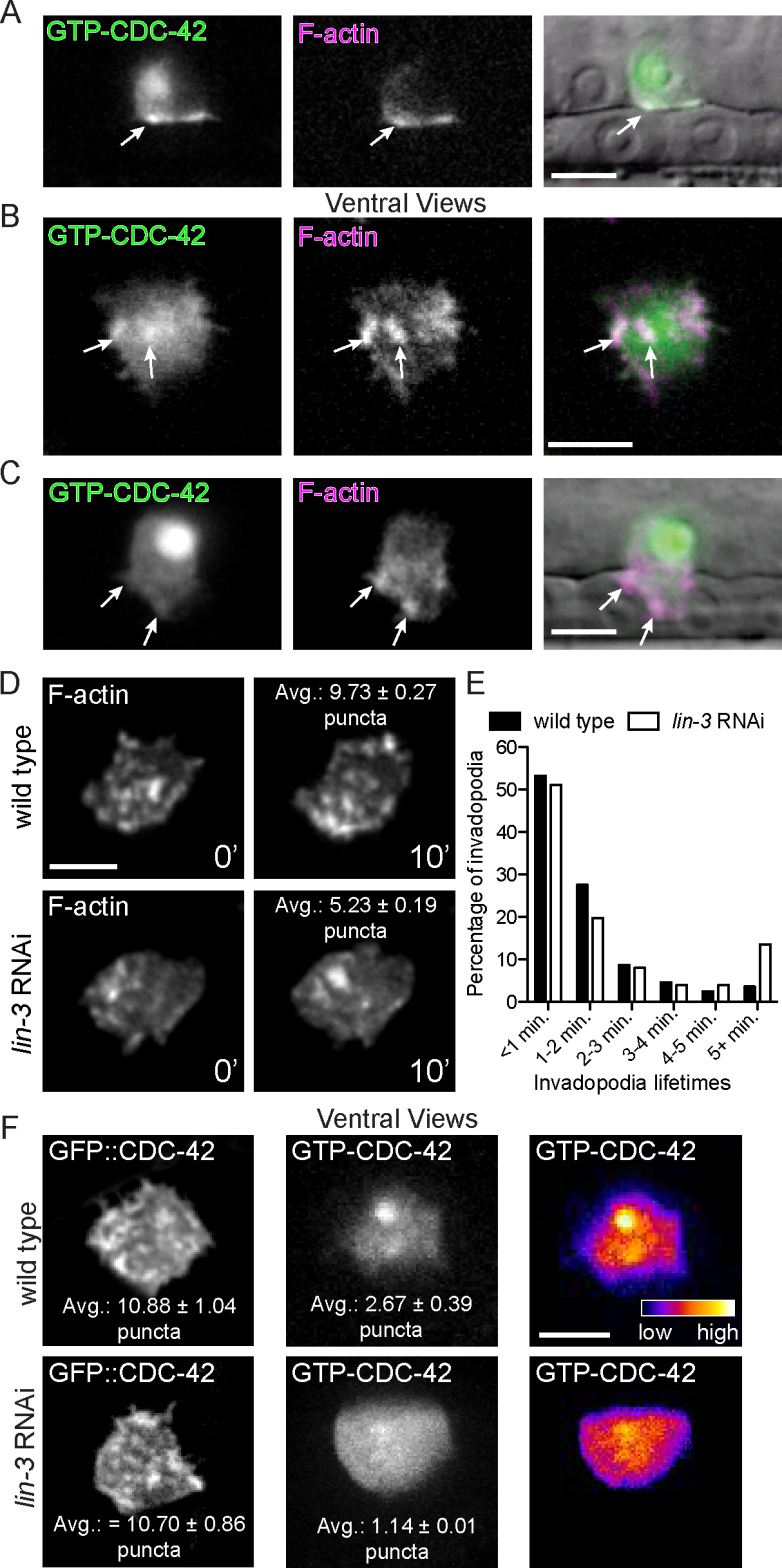
CDC-42 activation at the invasive membrane is dependent on vulval precursor cells. (A) Fluorescence images of a biosensor that reports active, GTP-bound CDC-42 (left; *cdh3>GFP*::*GBD*_*wsp-1*_) and F-actin (middle; *cdh3>mCherry*::*moeABD*) overlaid on DIC image (right). Prior to BM breach, GTP-CDC-42 and F-actin show a similar, punctate distribution at the AC invasive membrane (arrows). Biosensor signal was also observed in the nucleus. (B) Ventral views show that GTP-CDC-42 (left) concentrates in a punctate pattern (arrows) that overlaps with invadopodial F-actin (middle; right). (C) After BM breach, GTP-CDC-42 (left) did not accumulate in the invasive protrusion (arrows), which is enriched with F-actin (middle; merged images right). (D) A ventral time series shows fewer invadopodia (visualized with F-actin) in animals lacking vulval cells (*lin-3* RNAi; bottom) relative to wild type (top) (overlaid text reports average number of invadopodia per timepoint from 6 wild type animals and 7 *lin-3* RNAi animals; p < 0.0001, Student’s t-test). (E) Animals treated with *lin-3* RNAi have longer-lived invadopodia (n = 6 wild type and 7 *lin-3* RNAi treated animals; p < 0.01, Chi-square test). (F) Ventral views showing that the wild type, punctate pattern of GFP::CDC-42 (top left) was not affected by loss of the vulval precursor cells (*lin-3* RNAi; bottom left; overlaid text reports average number of puncta from 8 wild type animals and 10 *lin-3* RNAi treated animals; p = 0.90, Student’s t-test). GTP-CDC-42 puncta (middle; right), however, were decreased with loss of the vulval cells (n = 14 wild type animals and 18 *lin-3* RNAi treated animals; p < 0.01, Student’s t-test). Scale bars = 5 μm.

The Cdc42 GTPase switches between an active GTP-bound form and an inactive GDP-bound state. Cdc42 interacts with and activates effectors such as N-WASP when bound to GTP [36,40]. To determine the subcellular site of CDC-42 activation, we created transgenic animals that express an AC-specific genetically encoded sensor for active, GTP-bound CDC-42 (*cdh-3>GFP*::*GDBwsp-1*) ([41]; see Methods). Lateral imaging revealed that GTP-CDC-42 was polarized to the invasive membrane and the probe also showed signal in the nucleus (Fig 3A). We suspect that the nuclear concentration does not represent active GTP-CDC-42, as rescuing GFP::CDC-42 was not localized in the nucleus (see Fig 1C). We thus focused on GTP-CDC-42 at the invasive membrane. Examining ventral views of the AC, we found that GTP-CDC-42 localized to puncta that overlapped with invadopodial F-actin (Fig 3B). Furthermore, we found that GTP-CDC-42 was enriched at 50% of small BM breaches (< 4 um^2^; present in 20/40 breaches), indicating that CDC-42 is often active at the site of initial BM penetration. When the total area of BM cleared was larger than 4 um^2^, however, GTP-CDC-42 was no longer enriched at breaches (present in 4/18 breaches). In addition, GTP-CDC-42 was not localized to the large invasive protrusion that forms after BM breaching (Fig 3C; detected in 1/7 protrusions). Most ACs rapidly formed an invasive protrusion after BM breach in *cdc-42* mutants (n = 6/8 animals), consistent with invasive protrusion generation being independent of CDC-42 activity. Taken together, these observations suggest that CDC-42 is activated at the invasive membrane where it promotes invadopodia formation, but that it is not required for invasive protrusion formation.

A number of extracellular signals have been implicated in stimulating invadopodia in cell culture, mouse, and zebrafish cancer models [42–46]. It has been proposed that these signals activate Cdc42 [31]; however, direct confirmation of this notion is lacking for most invadopodia induction events. We have previously shown that a diffusible chemotactic cue(s) from the primary fated vulval precursor cells (the tissue that the AC invades) stimulates and targets invasion [21]. To determine if the vulval cue regulates invadopodia formation and CDC-42 activation, we examined animals lacking the vulval precursor cells (vulvaless animals; *lin-3* RNAi treatment; see Methods) and observed fewer invadopodia (Fig 3D; S3 Movie; Table 2). Notably, loss of the vulval precursor cells did not affect the distribution of GFP::CDC-42 at the invasive cell membrane (Fig 3F), but the number of activated GTP-CDC-42 puncta was reduced nearly three fold (1.14 ± 0.36 versus 2.67 ± 0.39 in wild-type; Fig 3F). To genetically test if CDC-42 functions in the vulval cue pathway, we performed epistasis analysis. If CDC-42 functioned solely in the vulval cue pathway, loss of *cdc-42* activity in vulvaless animals should not enhance the invasion defect of animals lacking vulval precursor cells. Supporting this idea, loss of *cdc-42* did not augment the AC invasion defect of vulvaless animals (Table 1). However, unlike loss of *cdc-42*, the invadopodia in animals lacking vulval cells were larger and longer-lived (Table 2; Fig 3E), suggesting that in addition to promoting CDC-42 activation, the vulval cells also regulate other aspects of invadopodia. Together, these data offer compelling evidence that CDC-42 is activated downstream of a pro-invasive cue from the primary vulval precursor cells to promote invadopodia formation.

### Rab GDI-1 functions in the AC to promote invadopodia formation

We were next interested in determining whether our sensitized screen identified new regulators of invadopodia formation, dynamics, or function. A gene whose loss strongly enhanced the *unc-40* invasion defect was *gdi-1*, which encodes a Rab GDP-dissociation inhibitor (GDI) (Table 1). Rab GDIs have high affinity for GDP-bound Rab proteins and are thought to deliver Rabs to specific membrane compartments to direct membrane trafficking [47].

**Fig 4:**
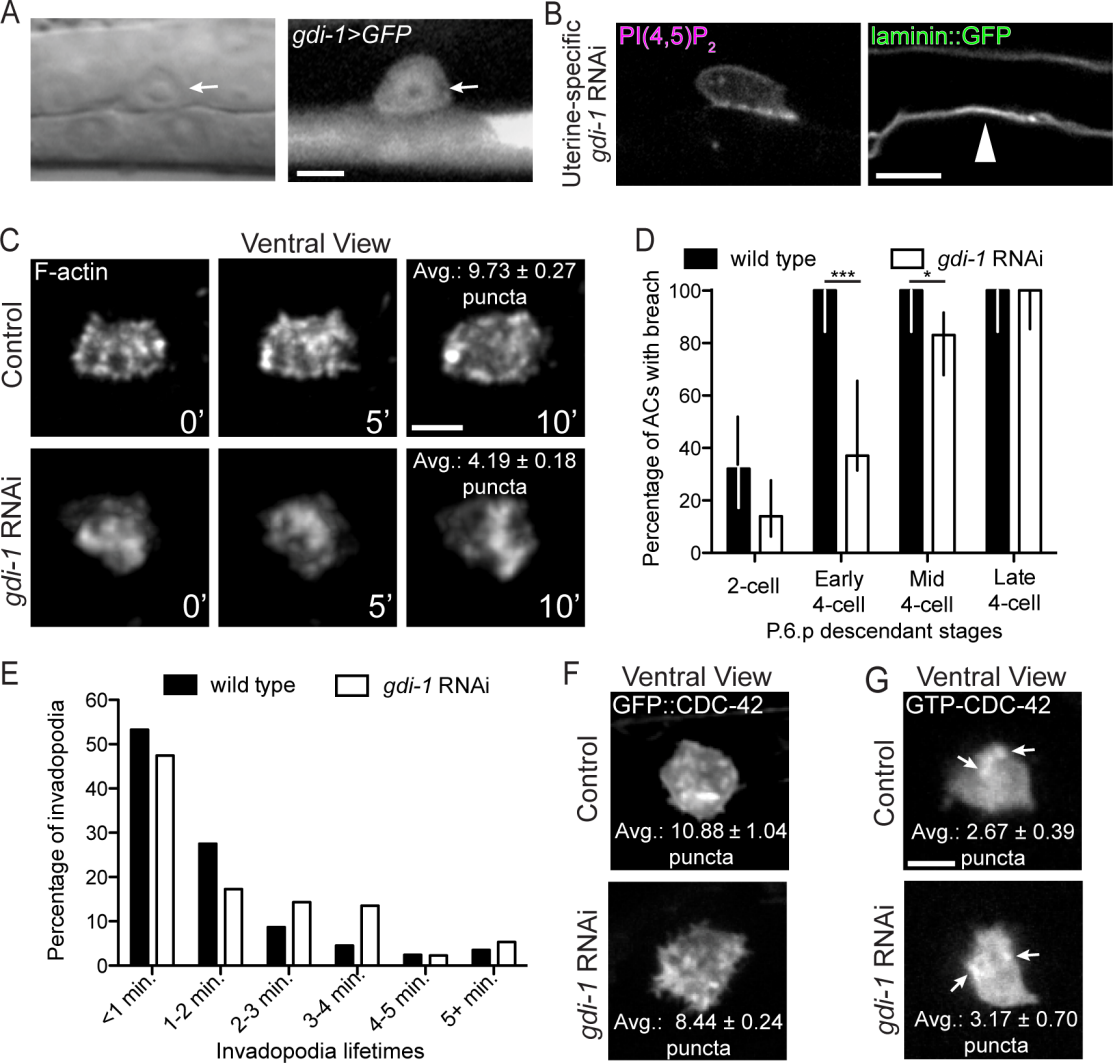
GDI-1 regulates invadopodia formation. (A) A transcriptional reporter (*gdi-1>GFP*) revealed *gdi-1* expression in the AC (arrow). (B) Uterine-specific RNAi against *gdi-1* blocked AC invasion (arrowhead marks intact BM). (C) A ventral time series shows decreased invadopodia (marked with F-actin) formation in animals with reduced *gdi-1* (RNAi; bottom) relative to wild type (top; overlaid text reports average number of invadopodia per timepoint from 6 wild type animals and 5 *gdi-1* RNAi treated animals; p < 0.0001, Tukey’s multiple comparisons test). (D) BM breaching was delayed in *gdi-1* RNAi treated animals (n ≥ 25 animals per stage, per genotype; *** p < 0.0001, * p < 0.05, Fisher’s exact test; bars represent 95% confidence intervals). (E) Animals treated with *gdi-1* RNAi have longer-lived invadopodia (n = 6 wild type and 7 *gdi-1* RNAi treated animals; p < 0.01, Chi-square test). (F) Ventral views showing that the wild type, punctate pattern of GFP::CDC-42 (top) was not affected by loss of *gdi-1* (bottom; overlaid text reports average number of puncta from 8 wild type animals and 9 *gdi-1* RNAi treated animals; p = 0.05, Student’s t-test). (G) CDC-42 was still activated normally (as visualized with the GTP-CDC-42 sensor) after loss *gdi-1* (overlaid text reports average number of puncta from 18 wild type animals and 6 *gdi-1* RNAi treated animals; p = 0.53, Student’s t-test). Scale bars = 5 μm.

To determine where *gdi-1* is expressed we built a transcriptional reporter for *gdi-1* (*gdi-1>GFP*) and found that *gdi-1* is upregulated in the AC and the vulval precursor cells prior to and throughout invasion (Figs 4A and S2). RNAi targeting *gdi-1* results in embryonic lethality [48]. Consistent with this observation, we found that a putative null deletion allele of *gdi-1(tm660)* was embryonic lethal (see Methods). Thus, to avoid embryonic lethality, we used RNAi targeting *gdi-1* beginning at the L1 stage, which resulted in a highly penetrant AC invasion defect in the L3 stage (Table 1). Further, uterine-specific RNAi knockdown of *gdi-1* also disrupted AC invasion (n = 33/100 AC failed to breach BM at the mid P6.p four-cell stage; Fig 4B; Table 1), while vulval specific RNAi did not alter invasion (Table 1). Given that neighboring uterine cells do not regulate invasion, these observations strongly suggest that GDI-1 functions in the AC. We next generated an AC-specific N-terminal GFP fusion to GDI-1 (*cdh-3>GFP*::*gdi-1*). Full length GFP::GDI-1 localized to the cytosol, similar to vertebrate GDIs (S2 Fig) [49,50]. Demonstrating that the RNAi construct efficiently targets *gdi-1*, the level of AC-specific GFP::GDI-1 was reduced by 73.6% in *gdi-1* RNAi treated animals relative to controls (S2 Fig). To further confirm the specificity of *gdi-1* RNAi, we generated a construct in which *gdi-1* from the related nematode *C*. *briggsae* was specifically expressed in the AC (*cdh-3>GFP*::*Cbrgdi-1*). The *C*. *briggsae* and *C*. *elegans* genes share 86% identity, which is below the 95% threshold required for RNAi targeting [51]. Expression of *GFP*::*Cbrgdi-1* in the AC restored invasion in animals treated with *C*. *elegans gdi-1* RNAi (*n* = 52/57 (91%) normal invasion, 3/57 (5%) partial invasion, 2/57 (4%) no invasion; Table 1). Taken together, these data confirm the specificity of the *C*. *elegans gdi-1* RNAi and indicate that *gdi-1* functions within the AC cytosol to promote invasion.

We next examined whether GDI-1 was required for invadopodia formation or activity. Similar to *cdc-42*, we found that loss of *gdi-1* decreased the rate of invadopodia formation by nearly 50% (1.60 per minute compared with 2.75 per minute in wild type; Table 2), reduced the number of invadopodia, and delayed BM breaching (Table 2; Fig 4C and 4D; S4 Movie). However, unlike loss of *cdc-42*, RNAi-mediated reduction of *gdi-1* resulted in larger invadopodia that had longer lifetimes (Table 2; Fig 4E). We conclude that GDI-1 is necessary for proper invadopodia formation.

### Rab GDI-1 appears to function independently of CDC-42 to regulate invasion

**Fig 5:**
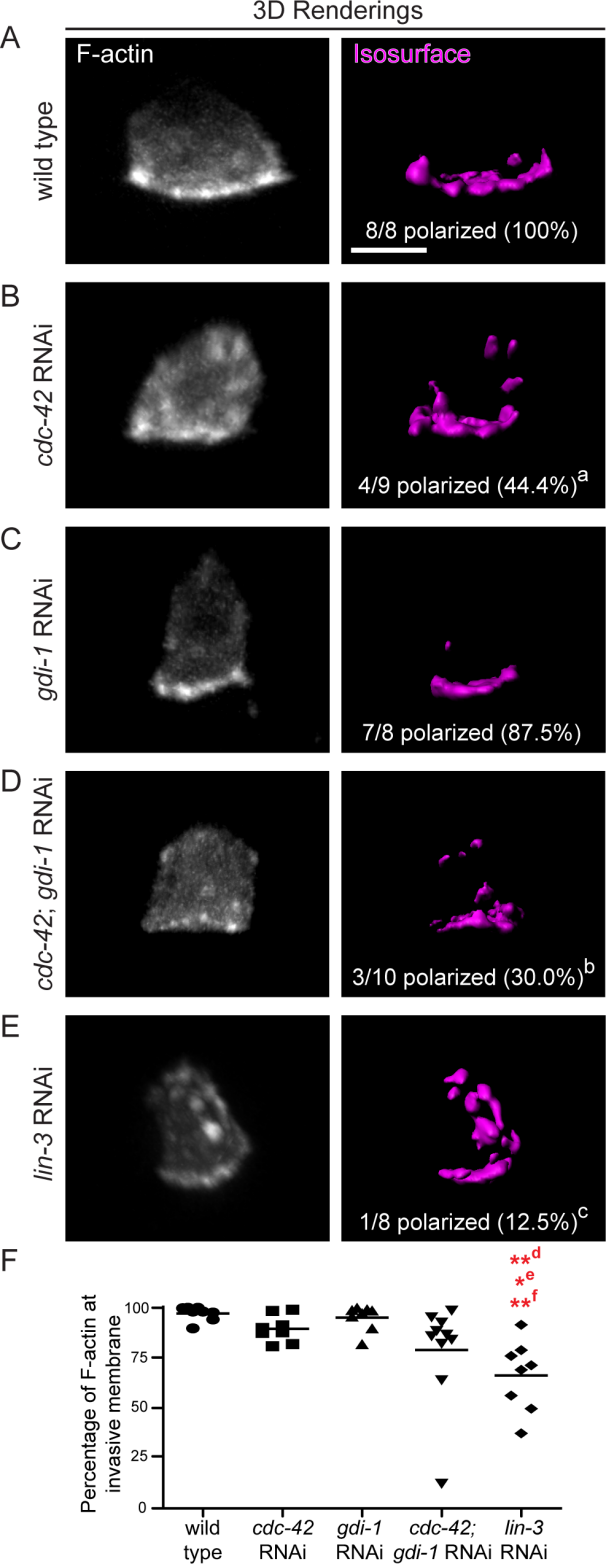
F-actin polarization is dependent on the vulval precursor cells and CDC-42. (A-E) 3D projections (left) and isosurface renderings of a fluorescent F-actin probe (*cdh3>mCherry*::*moeABD*) in wild type animals (A), animals treated with RNAi against *cdc-42* (B), RNAi agianst *gdi-1* (C), double RNAi targeting *cdc-42* and *gdi-1* (D, *cdc-42; gdi-1* RNAi), and animals lacking the vulval precursor cells (E, *lin-3* RNAi). F-actin was mispolarized after targeting *cdc-42* (^a^ p < 0.05), *cdc-42* and *gdi-1* in combination (^b^, p = 0.004), and the vulval precursor cells (*lin-3* RNAi; ^c^ p = 0.001). Comparisons were made using Fisher’s exact tests. (F) Scatter plot showing the percentage of F-actin polarized to the invasive membrane (line shows mean). The most severe of F-actin mispolarization defect was observed after loss of the vulval precursor cells (*lin-3* RNAi; n > 8 animals per group; **^d^ p < 0.01 vs. wild type, *^e^ p < 0.05 vs. *cdc-42* RNAi, **^f^ p < 0.01 vs. *gdi-1* RNAi). Comparisons were made using a Tukey’s multiple comparisons test. Scale bars = 5 μm.

We next tested the interaction between *gdi-1* and *cdc-42* in regulating invadopodia and invasion. Loss of *gdi-1* did not affect the punctate localization of total GFP::CDC-42 or activated GTP-CDC-42 at the invasive membrane (Fig 4F and 4G), suggesting that *gdi-1* does not regulate CDC-42. Furthermore, RNAi mediated knockdown of *gdi-1* in homozygous *cdc-42(gk388)* mutants significantly enhanced the *cdc-42(gk388)* invasion defect (Table 1), indicating that *gdi-1* has functions outside of *cdc-42* that promote invasion. Due to the difficulty of expressing probes to view invadopodia in *cdc-42* mutants, which were unhealthy from pleiotropic effects due to loss of *cdc-42*, we performed double RNAi targeting both *cdc-42* and *gdi-1 (cdc-42; gdi-1* RNAi) to examine invasion and invadopodia dynamics. This treatment slightly enhanced the invasion defect associated with loss of either *gdi-1* or *cdc-42* alone (Table 1), but it was not significant, indicating we did not achieve efficient knockdown of each gene. Notably, however, the combined *cdc-42; gdi-1* RNAi treated worms showed a trend of further reducing invadopodia formation rate (Table 2) and mispolarization of F-actin compared to loss of *cdc-42* or *gdi-1* alone (Fig 5A–5D and 5F). Although our data do not formally rule out that *cdc-42* and *gdi-1* act together in some aspects of invadopodia regulation, the genetic interaction and cell biological observations support the idea that *gdi-1* and *cdc-42* control distinct aspects of invadopodia formation.

### Rab GDI-1 acts downstream of the vulval cue

Loss of the vulval precursor cells lead to a more severe invasion defect than loss of *cdc-42* alone (Table 1), suggesting the vulval cue(s) controls another pathway(s) that promotes invasion. Thus, we wanted to next determine if *gdi-1* is a component of the vulval cue pathway with functions separate from *cdc-42*. Loss of *gdi-1* did not enhance the AC invasion defect of vulvaless animals (Table 1), indicating that GDI-1 promotes invasion through a pathway regulated by the vulval cue.

We next examined invadopodia formation in vulvaless animals. If the vulval cue controls separate pathways (such as one controlling CDC-42 and an independent pathway involving GDI-1) that converge to promote invadopodia formation, loss of the vulval precursor cells should more severely perturb invadopodia formation relative to loss of *cdc-42* or *gdi-1* alone. Consistent with this notion, vulvaless animals showed a greater reduction in rate of invadopodia formation than loss of either of *gdi-1* or *cdc-42* (0.86 per minute versus 1.60 per minute and 1.72 per minute, respectively; Table 2). Further, vulvaless animals showed an increase in the proportion of invadopodia with lifetimes greater than five minutes relative to loss of *gdi-1* or *cdc-42* (see Figs 1F, 3E and 4E). Finally, loss of the vulval cells resulted in a more severe mispolarization of F-actin throughout the cell compared to loss of *cdc-42* or *gdi-1* (Fig 5B–5F). These data are consistent with the vulval cue controlling the CDC-42 and GDI-1 pathways (and possibly other pathways) to promote invadopodia formation at the invasive cell membrane.

### GDI-1 regulates invadopodial membrane trafficking to the invasive cell membrane

**Fig 6:**
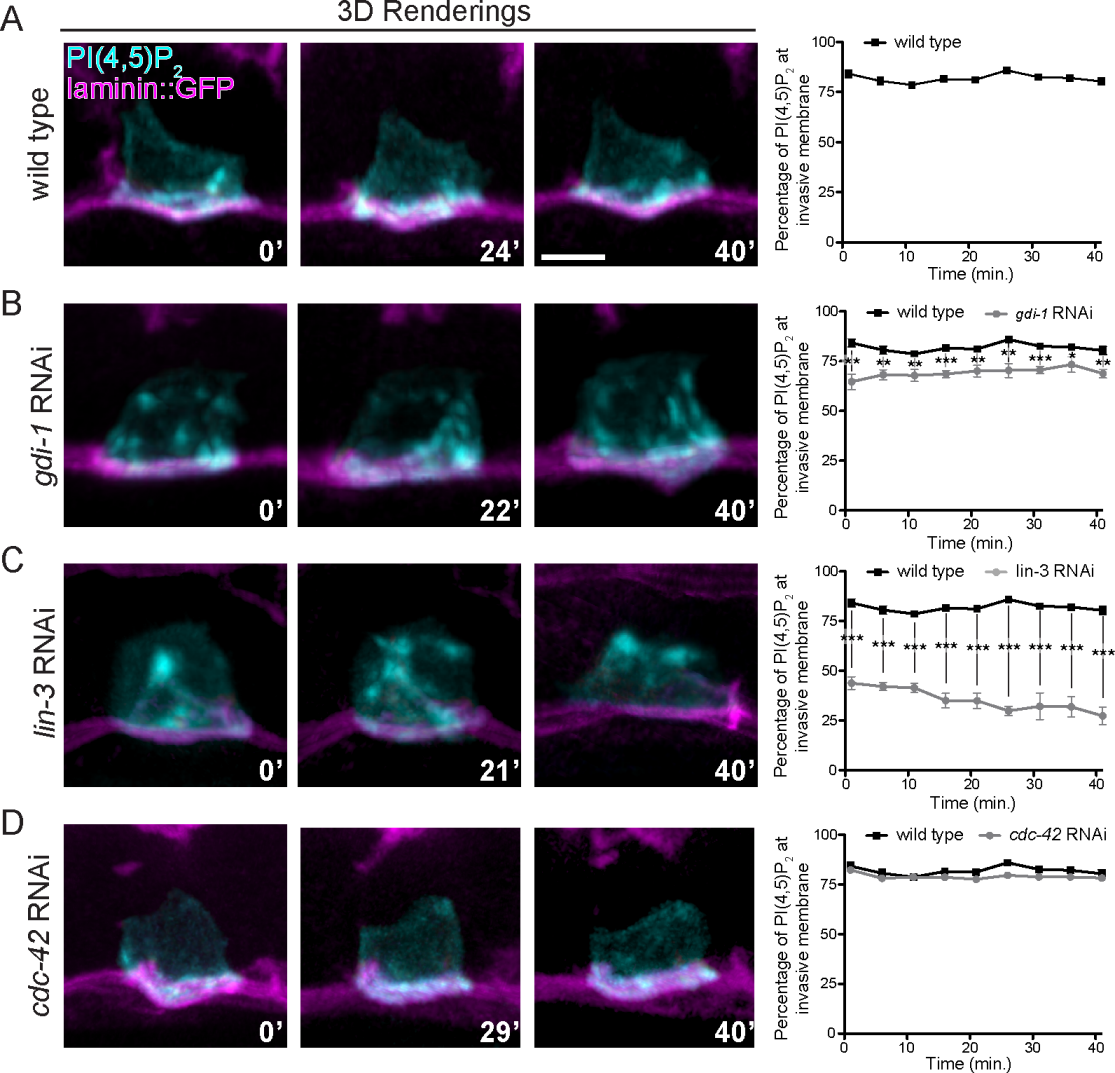
GDI-1 and the vulval precursor cells regulate invadopodial membrane trafficking. (A-D) Laterally viewed time series showing dynamic trafficking of the invadopodial membrane (visualized with marker for PI(4,5)P_2,_ *cdh3>mCherry*::*PLC*δ^*PH*^). Graphs show the average percentage of total PI(4,5)P_2_ fluorescence present at or near the basal invasive cell membrane of the AC plotted over time (n = 5 animals per group). (A) In wild type animals PI(4,5)P_2_ was trafficked at the ACs invasive cell membrane and remained strongly polarized over time. (B, C) In animals with reduced *gdi-1* function (B) and animals lacking vulval precursor cells (C; *lin-3* RNAi), PI(4,5)P_2_ trafficking was not restricted to the basal invasive membrane and PI(4,5)P_2_ was found in apical and lateral membranes. (D) Animals with reduced *cdc-42* function (RNAi) trafficked PI(4,5)P_2_ normally. * p < 0.05, ** p < 0.01, *** p < 0.001, Student’s t-test. Scale bars = 5 μm.

We have recently shown that a unique invadopodial membrane is dynamically recycled through the endolysosome to the invasive cell membrane to form invadopodia [23]. Given the role of Rab GDIs in membrane trafficking, we examined whether GDI-1 is required for proper trafficking of the invadopodial membrane. Examination of the invadopodial membrane components PI(4,5)P_2_ (mCherry::PLCδ^PH^) and the RAC GTPases MIG-2 and CED-10 revealed that RNAi-mediated knockdown of *gdi-1* led to inappropriate localization of the invadopodial membrane in apical and lateral plasma membrane domains (S3 Fig). In addition, the endolysosome markers CUP-5 and LMP-1, which normally polarize to the invasive membrane and colocalize with invadopodial membrane components [23,52,53], were less polarized following loss of *gdi-1* (S3 Fig). Time-lapse imaging of the invadopodial membrane marker PI(4,5)P_2_ in wild type animals revealed that the invadopodial membrane was dynamically trafficked to invadopodia at the invasive membrane and remained polarized over time (Fig 6A; S5 Movie). Following loss of *gdi-1*, the invadopodial membrane was still actively trafficked, but it was dynamically mis-targeted to lateral and apical plasma membrane domains (Fig 6B; S6 Movie). Notably, the invadopodial membrane was severely mis-targeted to apical and lateral plasma membrane domains in vulvaless animals (Fig 6C; S7 Movie), consistent with the idea that GDI-1 is a component of a pathway regulated by the vulval cue. In contrast to loss of *gdi-1* and vulvaless animals, loss of *cdc-42* did not alter the polarization or targeting of the invadopodial membrane (Figs 6D and S3; S8 Movie), further supporting the notion that CDC-42 and GDI-1 regulate distinct aspects of invadopodia formation. Taken together, these observations suggest that GDI-1 acts downstream of the vulval cue to target trafficking of the invadopodial membrane to the site of invadopodia formation at AC’s invasive cell membrane.

### Loss of GDI-1 does not disrupt global membrane trafficking and polarity in the AC

**Fig 7:**
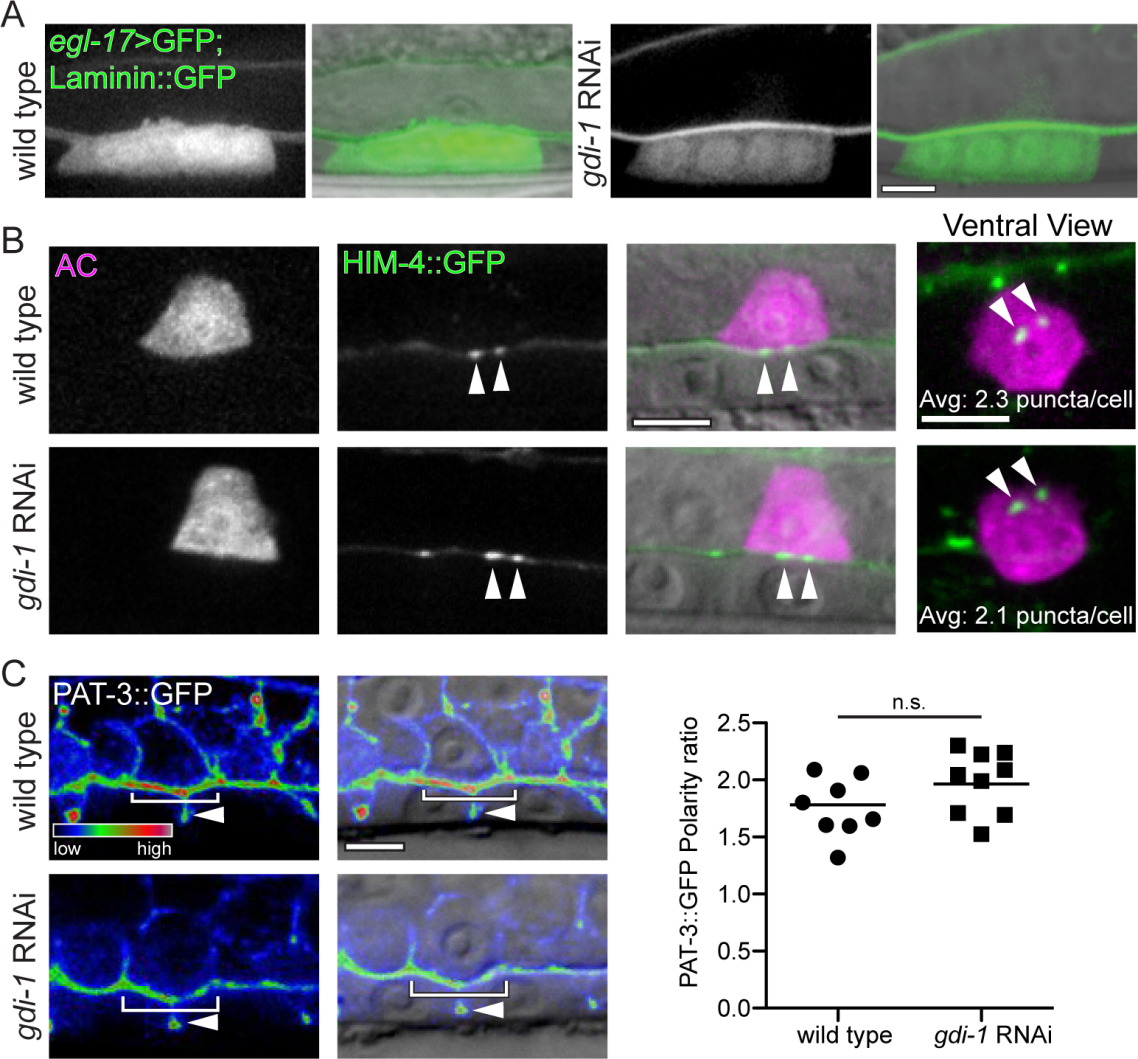
Loss of *gdi-1* does not affect other AC trafficking and polarization processes. (A) In wild type animals (left) the primary vulval cells (expressing *egl-17>GFP*) are specified in response to the EGF-like ligand LIN-3 secreted by the AC. Primary vulval cell specification was normal after loss of *gdi-1* (RNAi; n > 10 in each group) indicating proper AC trafficking and secretion of LIN-3. (B) The extracellular matrix component HIM-4 (*cdh3>him-4*::*GFP*) is secreted by the AC (visualized with *cdh3>mCherry*) and forms puncta within the BM of wild type animals (top; arrowheads). HIM-4::GFP deposition into the BM was unaffected by loss of *gdi-1* (bottom; arrowheads). In ventrally viewed images, the number of puncta was normal (overlaid text reports average number of puncta from 8 wild type and 9 *gdi-1* RNAi treated animals; p = 0.80; Student’s t-test). (C) The β-integrin subunit PAT-3::GFP (left; *pat-3>pat-3*::*GFP*) overlaid on a DIC image (right). PAT-3::GFP is polarized along the AC invasive membrane (top panels; brackets) and is also found between the vulval precursor cells (arrowheads). Loss of *gdi-1* (RNAi; bottom panels) did not alter PAT-3::GFP enrichment at the invasive membrane (brackets). Scatter plot shows the ratio of PAT-3::GFP at the AC invasive membrane relative to the apicolateral membrane (line shows the mean; n = 8 wild type and 9 *gdi-1* RNAi treated animals; n.s. = not significant, p = 0.11, Student’s t-test). Scale bars = 5 μm.

Lastly, we wanted to determine if the disruption of invadopodial membrane trafficking was a specific defect of the invadopodial membrane compartment or a more general perturbation of intracellular membrane trafficking and cell polarization. We thus examined other markers of AC secretion and polarization. The AC secretes the EGF-like ligand LIN-3, which induces vulval formation and expression of *egl-17* [54]. Induction of *egl-17* expression in the vulval cells was normal after loss of *gdi-1*, indicating that the AC properly traffics and secretes LIN-3 (Fig 7A). In addition, loss of *gdi-1* did not affect AC secretion and deposition of the extracellular matrix protein hemicentin into the BM (Fig 7B) [55]. Finally, RNAi targeting of *gdi-1* did not alter the polarization of the integrin receptor INA-1/PAT-3 to the AC invasive cell membrane (Fig 7C) [24]. These results strongly suggest that loss of *gdi-1* does not globally disrupt membrane transport or cell polarity, but instead specifically perturbs invadopodial membrane trafficking.

## Discussion

**Fig 8:**
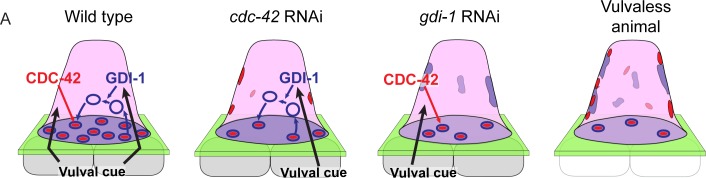
A Model of CDC-42 and GDI-1 function in promoting invadopodia formation. (A) A secreted cue or cues from the vulval precursor cells coordinates the activities of CDC-42 and F-actin formation (red puncta) at the invasive membrane by activating CDC-42 and through an unknown mechanism also regulates a GDI-1-dependent pathway that directs invadopodial membrane trafficking (blue) to invadopodia. Loss of *cdc-42* leads to a decrease in the number of invadopodia and some mispolarized F-actin, while loss of GDI-1 results in mistargeting of the invadopodial membrane to apical and lateral plasma membranes and also a reduction in the number of invadopodia. Absence of the vulval cells leads to severe disruptions in invadopodia formation, invadopodial membrane trafficking, and F-actin formation and polarization.

Invadopodia are protrusive subcellular structures that mediate BM breaching during normal and pathological cell invasion events and are promising targets for anticancer therapies [11–13,56]. Despite their fundamental biological and medical importance, the mechanisms regulating these dynamic structures in vivo are poorly understood [19,20,57]. Using AC invasion into the vulval epithelium in *C*. *elegans*, we conducted the first large-scale screen for genes regulating invadopodia in vivo. We characterized two of 13 genes identified: the Rho GTPase *cdc-42* and the Rab GDP dissociation inhibitor *gdi-1*. We show that these genes control distinct aspects of invadopodia formation. *cdc-42* controls F-actin generation at invadopodia, while *gdi-1* mediates trafficking of the specialized invadopodial membrane to the invasive cell membrane where invadopodia form. Further, we find that the separate aspects of invadopodia formation regulated by *cdc-42* and *gdi-1* are both controlled by a cue from the vulval cells that coordinates their activities to form invadopodia (see summary in Fig 8). Together our findings identify new genes regulating invadopodia in vivo and reveal the mechanisms by which an extrinsic pro-invasive signal coordinates distinct cellular processes to construct invadopodia during cell invasion.

### CDC-42 induces invadopodia formation in the AC

The Rho GTPase Cdc42 has been implicated as a central molecule in stimulating invadopodia in numerous cancer cell lines, including melanoma, glioblastoma, breast, and pancreatic tumor derived cells [31,58]. Through our screen we independently confirmed that the *C*. *elegans* ortholog of the Rho GTPase Cdc42 is a key molecule in promoting invadopodia formation in vivo. CDC-42 is expressed in the AC and is localized to invadopodia along the invasive cell membrane. Furthermore, we found that CDC-42 was activated at the invasive membrane and that CDC-42 activation was dependent on the vulval tissue that the AC invades. We have previously shown that the primary vulval precursor cells stimulate and target AC invasion with a diffusible cue(s) [21]. Our new observations support the idea that the vulval cue induces invadopodia formation through activation of CDC-42. This is consistent with observations in cancer cell lines, where external cues such as the EGF growth factor and collagen I fibers are thought to stimulate activation of Cdc42 and invadopodia formation [32,45].

We also find that in the AC, similar to mammary adenocarcinoma cells, the actin regulator and CDC-42 effector WSP-1 (N-WASP), functions downstream of CDC-42 to promote F-actin formation [32]. WSP-1 was localized to invadopodia and this localization was dependent on CDC-42. It is likely that this interaction is direct, as vertebrate Cdc42 activates and localizes N-WASP [36,39]. Notably, loss of *cdc-42* led to a greater reduction in invadopodia than loss of *wsp-1*, suggesting that CDC-42 acts through other downstream effectors in addition to WSP-1. A similar result was seen in mammary adenocarcinoma cells [37], suggesting that WASP proteins are not the sole effector of Cdc42 responsible for invadopodia formation.

In breast and pancreatic tumor cell lines, depletion of Cdc42 function results in a complete or near complete loss of invadopodia formation and matrix degradation [32,37,45,58]. In contrast, the loss of CDC-42 in the AC resulted in only an approximate 50% reduction in the rate and number of invadopodia formed and a one-hour invasion delay. Although we cannot rule out that there was some limited CDC-42 activity in the AC in our studies, these observations suggest that invadopodia are also stimulated through a CDC-42 independent mechanism. The apparent redundancies in the mechanisms that induce invadopodia in *C*. *elegans* might reflect the robustness of the genetic networks underlying AC invasion. The process of AC invasion is highly conserved across nematode species and is under intense evolutionary selective pressure, as defects in invasion perturb the egg-laying apparatus and decrease fecundity [28,59,60]. Transformed cancer cell lines may not possess these robust networks. Tumors in vivo, however, are heterogeneous and robust cell populations, and have many alternative molecular pathways to promote, growth, survival, and dispersal [61]. Thus, while our results support the notion that the Rho GTPase Cdc42 is a central initiator of invadopodia formation, they also suggest that multiple mechanisms for invadopodia induction exist and that these could be used in other contexts, including cancer cell invasion.

### Rab GDI-1 promotes invadopodial membrane trafficking

A combination of ultrastructural and live-cell imaging studies have revealed that the invadopodial membrane is dynamic and has unique structural and lipid properties [15,17,62]. We recently found that the invadopodial membrane of the AC is a unique membrane compartment, harboring lipid anchored Rac GTPases and the phospholipid PI(4,5)P_2_, that is actively recycled through the endolysosome during invadopodia formation and turnover [23]. The membrane associated matrix metalloproteinase MT1-MMP is actively recycled through the endolysosome to invadopodia in several tumor cell lines, suggesting this recycling pathway is a shared feature of invadopodia [63,64]. The invadopodial membrane may be crucial for targeted delivering of proteases such as MT1-MMP. In addition, the invadopodial membrane might be important in providing new membrane to allow for the rapid growth of protrusions through extracellular matrices.

Here we show that the Rab GDP dissociation inhibitor GDI-1 is a key regulator of invadopodial membrane trafficking and invadopodia formation. Using live-cell imaging we found that loss of GDI-1 resulted in invadopodial membrane that was still actively trafficked; however, it was not properly targeted to the invasive cell membrane where invadopodia form. This is in contrast to loss of UNC-60 (ADF/Cofilin), which leads to a severe reduction in invadopodial membrane trafficking to the cell surface and accumulation of static invadopodial membrane vesicles within the cell [23]. These results suggest that the correct targeting of invadopodial membrane depends on GDI-1, while UNC-60 (ADF/cofilin) is necessary for active trafficking or docking of invadopodial membrane to the plasma membrane. Importantly, neither loss of UNC-60 or GDI-1 altered other trafficking and polarization events in the AC [23], suggesting both are specific regulators of the invadopodial membrane.

Rab GTPases are crucial mediators of membrane trafficking that direct vesicle budding, vesicle movement, and vesicle fusion [65]. Rab GDIs are thought to regulate membrane trafficking by facilitating recycling of Rab proteins between target and donor membrane compartments [50]. Work in *Saccharomyces cerevisiae* has shown that amino acid residues required for Rab recognition in vitro are also required for the function of GDI in vivo, highlighting the dedication of GDI function to Rab regulation [66]. Rab GDIs show preferential interaction with different Rabs, signifying an ability to regulate specific membrane trafficking events [65,67]. However, we have not yet identified a Rab protein that promotes AC invasion in our screens [25,28], suggesting that GDI-1 interacts with multiple Rabs or a Rab essential for viability. Notably, the human ortholog of *gdi-1*, GDI-1β, is upregulated in numerous carcinomas, including pancreatic, esophageal, gastric, thyroid and gallbladder [68–72]. Further, GDI-1β is highly expressed in human medulloblastomas and stimulates invasion in a medulloblastoma cell line in vitro [73]. Thus, the function of GDI-1 in promoting invadopodial membrane trafficking and invasion may be conserved.

### Building invadopodia requires the coordination of multiple cellular pathways

Work from cell culture indicates that invadopodia are highly dynamic superstructures that require the coordinated activities of signaling proteins, F-actin generation, membrane trafficking, proteases, and cell adhesion [15,16]. Further, invadopodia form in response to numerous cues including growth factors, the extracellular matrix, and metabolic and hypoxia-induced factors [42]. How independent pathways within the cell are controlled and coordinated to form invadopodia in response to extracellular cues is poorly understood.

We have previously shown that the vulval precursor cells secrete an unidentified, diffusible cue(s) that promotes invasion [21]. Our genetic epistasis and cell biological observations indicate that this cue(s) from the vulval precursor cells regulates both CDC-42 directed F-actin generation and GDI-1 mediated invadopodial membrane trafficking to coordinate invadopodia formation. Other local interactions may link these independent cellular activities to build functional invadopodia. For example, the actin regulators Cdc42 and N-WASP, which promote F-actin formation, also have functions in directing vesicle fusion and concentration of MT1-MMP at invadopodia [64,74]. Our findings that combined loss of *gdi-1* and *cdc-42* enhanced the loss of invadopodia and the mislocalization of F-actin throughout the cell, suggests that CDC-42 and GDI-1 pathways do not simply combine one-to-one to form invadopodia. Rather, they may each link with additional unidentified pathways that promote invadopodia formation. For example, GDI-1 likely promotes invadopodial membrane trafficking to AC invadopodia that are initiated by CDC-42 as well as those formed independently of CDC-42 activity. As loss of the vulval cells led to dramatic defects in invadopodia formation and mislocalization of invadopodial components, we suspect that the vulval cue might regulate other aspects of invadopodia formation and that extracellular cues may generally function to coordinate distinct aspects of invadopodia formation to spatiotemporally control invadopodia construction and cell invasion.

## Methods

### Strains and culture conditions

Culturing and handling of *C*. *elegans* was done as previously described [75]. Wild type animals were strain N2. In the text and figures, we designated linkage to a promoter with a greater than symbol (>) and used a double colon (::) for linkages that fuse open reading frames. The alleles and transgenes used in this study were as follows: *qyIs8[lam-1>lam-1*::*GFP]; qyIs17[cdh-3>mCherry]; qyIs43[pat-3>pat-3*::*GFP; ina-1>ina-1]; qyEx45[cdc-42>GFP*::*cdc-42]; qyIs46[emb-9>emb-9*::*mCherry]; qyIs57[cdh-3>mCherry*::*moeABD]; qyIs61[cdh-3>GFP*::*unc-34]; qyIs204[wsp-1>GFP]; qyIs211[cdh-3>lmp-1*::*GFP]; qyIs212[cdh-3>GFP*::*wsp-1]; qyIs219[cdh-3>GFP*::*PLC*δ^*PH*^*]; qyIs220[cdh-3>mig-2*::*GFP]; qyIs221[cdh-3>ced-10*::*GFP]; qyIs409[cdh-3>GFP*::*cup-5]; qyIs410[cdh-3>GFP*::*cdc-42]; qyIs412[cdh-3>GFP*::*GBDwsp-1]; qyIs427[lam-1>lam-1*::*mCherry]; qyEx507[cdh-3>GFP*::*gdi-1]; qyEx515[gdi-1>GFP]; qyEx533[cdh3>GFP*::*Cbrgdi-1]; urIs[rol-6(1006); lam-1>lam-1*::*GFP];* LGI, *unc-40(e271); ayIs4[egl-17>GFP];* LGII, *cdc-42(gk388); mIn1;* LGIII, *unc-119(ed4); rhIs23[him-4>him-4*::*GFP];* LGIV, *wsp-1(ng324); qyIs10[lam-1>lam-1*:*GFP]; gdi-1(tm660); nT1;* LGV, *qyIs50[cdh-3>mCherry*::*moeABD];* LGX, *qyIs7[lam-1>lam-1*::*GFP]; qyIs24[cdh-3>mCherry*:: *PLC*δ^*PH*^*]*.

### Analysis of a putative null deletion allele of *gdi-1*

The deletion allele *gdi-1(tm660)* was obtained from the National Bioresource Project (NBRP; http://www.shigen.nig.ac.jp/c.elegans). The *gdi-1(tm660)* allele is a 541 bp deletion in the first exon that shifts the frame of the open reading such that only the first 37 amino acids of the protein are predicted to be made (out of 549 amino acids in the full protein). The *gdi-1(tm660)* allele is thus likely a null for *gdi-1* activity. The *gdi-1(tm660)* allele was balanced using the nT1 translocation by creating the strain, *gdi-1(tm660)* IV/nT1[*qIs51*] (IV;V). The *qIs51* transgene, which is linked to nT1, expresses GFP in the pharynx. The presence of *gdi-1* in the balanced strains was confirmed using the published NBRP primers: ExtRev:TCAAGGAGTGCATCATCTCG, ExtFwd:CCTGATCATTCAACGACAAG, IntFwd:GTGAGTGATGTTGGTGAAGT, IntRev:CTCGGGAATGCTGTCGGTTT. Balanced *gdi-1*/nT1 mothers never segregated non-fluorescent *gdi-1* homozyogous progeny (n = 0/54 examined) in timed egg-lays. Dead embryos were observed on the plates, but dead larvae were not seen. These results suggest that *gdi-1* is required for embryonic viability.

### Transgenes and molecular biology

Reporter constructs were generated by PCR fusion [76]. AC-specific promoter fusions were generated with the *cdh-3*(mk62-62) AC-specific regulatory element [76,77]. Transgenic worms were created by transformation with co-injection markers of pPDMM016B (*unc-119+*) into the germline of *unc-119(ed4)* worms. These expression constructs were injected with EcoRI-digested salmon sperm DNA and pBSSK DNA, along with serial dilutions of the fusion construct to optimize expression levels and avoid toxicity. Stably expressed extrachromosomal lines were established and selected lines were integrated by gamma irradiation. See S3 Table for primers used in generation of new transgenic strains. The GTP-CDC-42 biosensor was built by fusing the GTP-CDC-42 binding domain of WSP-1 to GFP under the AC-specific *cdh-3* promoter [41]. The *wsp-1>GFP* transcriptional reporter construct was built by fusing 7.3 kb of the 5’ cis-regulatory element of *wsp-1* to GFP. The AC-specific regulatory of the *cdh-3* promoter and GFP were fused to the open reading from of *wsp-1* to create the AC-specific GFP:WSP-1 construct. 2.5 kb of the putative 5’ cis-regulatory for *gdi-1* was fused to GFP to generate the transcriptional reporter strain. The AC-specific *C*. *elegans* and *C*. *briggsae gdi-1* translational reporters were built by fusing the *cdh-3>* AC-specific regulatory element to the species-specific full-length *gdi-1* genes and their respective 3’ UTRs.

### Scoring of AC invasion

Anchor cell invasion was scored as previously described using DIC microscopy [21,76]. Briefly, animals were scored for invasion at the primary vulval precursor P6.p four-cell stage when BM invasion is completed in wild type animals. Anchor cells were scored as “normal” invasion if there was a visible breach in the phase dense line at the mid-P6.p four-cell stage, “partial” if the breach in the phase dense line was smaller than the nucleus, and “blocked” invasion if the phase dense line remained intact. For timing of breach experiments, the vulval precursor cells divisions, the distal tip cell migrations, and the divisions of the ventral uterine cells were used to determine “early” and “late” two-cell and “early,” “mid,” and “late” four-cell stages.

### Creation of vulvaless animals

RNA interference (RNAi) targeting *lin-3* or *lin-3* mutants (*lin-3(n378)*, *let-59(s49)*, *unc-22(s7)*, *unc-31(e169)/lin-3(n1059)*, *unc-24(138)*, *dpy-20(e128); qyIs24[cdh-3>mCherry*:: *PLC*δ^*PH*^*]*) result in vulvaless worms that lack specification of primary vulval precursor cells and, therefore, do not generate the vulval cue(s) [21,28]. Staging of these worms was based on the distal tip cell migration and divisions of the ventral uterine cells.

### RNAi treatment

RNAi targeting open reading frames of genes or using an empty vector control (L4440) was delivered by feeding synchronized L1-arrested worms *E*. *coli* expressing double stranded RNA, as previously described [28]. Synchronized L1-arrested larvae were plated on fields of the RNAi-expressing bacteria, except worms homozygous for *cdc-42(gk388)* were plated on RNAi as freshly harvested eggs because homozygous *cdc-42* mutants do not survive L1 arrest well. All RNAi clones after the initial genome-wide screen were sequenced prior to use.

Tissue-specific RNAi experiments were preformed as previously described using *rrf-3(pk1426); qyIs102[fos-1a>rde-1; myo-2>GFP]; qyIs10[lam-1>lam-1*:*GFP]; rde-1(ne219); qyIs24[cdh-3>mCherry*:: *PLC*δ^*PH*^*]* worms for uterine-specific RNAi and *rrf-3(pk1426); qyIs138[unc-62>rde-1]; rde-1(ne219)* worms for vulval-specific RNAi [24,27,28]. Briefly, those experiments use animals possessing a null mutation of rde-1, a gene required for RNAi sensitivity [78]. A functional copy of RDE-1 is expressed specifically in a tissue of interest, thus restoring RNAi in those cells and allowing for determination of site-of-action.

### Sensitized genome-wide RNAi screen for genes regulating AC invadopodia

In the primary genome-wide RNAi screen in the *unc-40(e271)* mutant background, 50–100 synchronized L1-arrested worms were plated on RNAi for 70 hours at 20°C. Egg production was assayed by counting the number of laid eggs at this time point. Worms on control plates laid an average of 100 eggs and experimental worms were scored as (-) when no eggs were laid, (+) when there was a moderate reduction in the number of eggs laid, and (++) when the number of eggs laid was decreased by more than 50%. Worms were also scored for the protruding vulval phenotype in three categories: (#) when 10% of the worms had protruding vulvas, (##) when 20% of worms had protruding vulvas, and (###) when more than 50% of worms exhibited this phenotype. Candidate genes for the secondary screen for AC invasion defects were identified using AmiGO v1.8 [79]. These genes were selected using the GO terms “extracellular space” (GOID:0005615), “integral component of membrane” (GO:0016021), “GPCR signaling” (GOID:0007186), and “GTPase regulator activity” (GOID:0030695).

### Microscopy, image acquisition, processing, and analysis

Images were acquired using an EM-CCD camera (Hamamatsu Photonics) and a spinning disk confocal (CSU-10; Yokogawa) mounted on a microscope (AxioImager; Carl Zeiss) with a Plan-APOCHROMAT 100x/1.4 oil differential objected controlled by MicroManager software [80]. Acquired images were processed using ImageJ 1.40g and Photoshop (CS3 Extended, Adobe) and smoothened using a 0.5 pixel radius Gaussian blur filer. 3D reconstructions were built from confocal z-stacks, analyzed, and exported.mov files using IMARIS 7.4 (Bitplane, Inc.). Figures were constructed using Illustrator (CS3 Extended, Adobe).

Quantitative analyses of AC invadopodia and BM breach were done using ImageJ, Imaris, or both. For time-lapse microscopy, worms were anesthetized in 0.2% tricaine and 0.02% levamosile in M9 and then transferred to 5% noble agar pads, sealed with VALAP, and imaged at 23°C at one minute time intervals [22]. For consistent measurements, isosurface renderings, built in place of polymerized F-actin at the invasive membrane, were used to determine a threshold for assigning the spots that were used to quantify AC invadopodia number, size, and dynamics on blinded time-lapses [22]. Average invadopodia number and size were blindly calculated from more than five 24-minute time-lapses taken at one-minute intervals for each group. Rate of invadopodia formation was calculated by determining the number of new invadopodia appearing in each frame during the first five minutes of each time-lapse. Invadopodia lifetimes were measured by determining the number of frames in which each invadopodium was present during a ten-minute portion of each time-lapse.

Quantification of GFP::CDC-42, GTP-CDC-42, and GFP::WSP-1 foci was performed in a blinded manner using the manual threshold and particle counting with the watershed filter functions in ImageJ on max projections of the two confocal z-planes that comprised the invasive membrane. The size of BM breach was calculated by using the threshold and measurement functions in ImageJ. Colocalization measurements were performed using the Imaris coloclaization module on blinded images. The enrichment of invadopodial membrane components and PAT-3::GFP at the invasive membrane were performed using ImageJ. For markers of the invadopodial membrane compartment, polarity measurements were calculated from sum projections that were thresholded manually. The proportion of thresholded signal in a region of interest (ROI) containing only the invasive membrane relative to the entire AC was then calculated. PAT-3::GFP calculations were performed as previously described [23,24]. Briefly, the average intensity from a five pixel wide line scan of the AC invasive membrane was divided by the average intensity from a line scan of the apicolateral AC membrane. The fluorescence intensities of GFP::GDI-1 and GFP::CbrGDI-1 were measured from sum projections of confocal z-stacks acquired with the same exposure times using ImageJ. The integrated density was measured in an ROI containing the AC and the average was calculated from at least 13 animals in each group.

### Statistical analysis

Statistical analysis was preformed using JMP version 9.0 (SAS Institute) or Graphpad PRISM v. 5, using two-tailed Fisher’s Exact test, a two-tailed unpaired Student’s t-test, or nonparametric Wilcoxon rank-sum test. Figure legends specify when each test was used.

## Supporting Information

S1 Table Genome-wide RNAi screen for enhancement of "egl" and "pvl" phenotypes in *unc-40(e271)*. (DOCX)

S2 Table Genes screened for AC invasion defects in *unc-40(e271)*. (DOCX)

S3 Table List of transgenes created and primers used. (DOCX)

S1 Fig *cdc-42* knockdown does not affect the composition of the reduced number of invadopodia that form. (A, B) Ventral views showing the composition of invadopodia. Top panels: the invadopodial markers GFP::UNC-34 (A; *cdh3>GFP*::*unc-34*) and PI(4,5)P_2_ (B, *cdh3> mCherry*::*PLCδ*^*PH*^) colocalize with F-actin (*cdh3>mCherry*::*moeABD*; arrowheads; overlaid text report Pearson’s colocalization coefficients, r). Bottom panels: Knockdown of *cdc-42* by RNAi reduces the number of invadopodia, but GFP::UNC-34 (A) and PI(4,5)P_2_ (B) still colocalize with F-actin (arrowheads). The correlation coefficients are not different between wild type animals and animals treated with *cdc-42* RNAi (n > 10 animals for each condition; p = 0.56 (GFP::UNC-34) and 0.26 (PI(4,5)P_2_, Student’s t-tests). Scale bar, 5 μm.
(TIF)

S2 Fig *gdi-1* is upregulated in the AC throughout invasion. (A) A transcriptional reporter (*gdi-1>GFP*) revealed *gdi-1* expression in the AC and vulval precursor cells throughout invasion. Expression of *gdi-1* is specifically upregulated in the AC. (B) An AC-specific full-length fusion of GFP to GDI-1 (*cdh3>GFP*::*gdi-1*) showed cytosolic distribution with no specific subcellular enrichment. (C, D) The *gdi-1* RNAi construct reduced levels of an AC expressed GDI-1 reporter (*cdh3>GFP*::*gdi-1*; n = 15 wild type and 14 *gdi-1* RNAi treated animals; *** p < 0.0001, Student’ t-test). (E, F) The *gdi-1* RNAi construct from *C*. *elegans* did not alter the fluorescence intensity of a GDI-1 reporter made from the related nematode *C*. *briggsae* (*cdh3>GFP*::*Cbrgdi-1*; n = 15 wild type and 13 *gdi-1* RNAi treated animals; p = 0.586, Student’s t-test; n.s = not significant). AU = arbitrary units; scale bar, 5 μm.
(TIF)

S3 Fig *gdi-1* is required for proper trafficking of the invadopodial membrane. (A-E) 3D renderings showing the distribution of PI(4,5)P_2_ (A, *cdh3> mCherry*::*PLCδ*^*PH*^), GFP::MIG-2 (B, *cdh3>GFP*::*MIG-2*), GFP::CUP-5 (C, *cdh3>GFP*::*cup-5*), GFP::CED-10 (D, *cdh3>GFP*::*ced-10*), and LMP-1::GFP (E, *cdh3>lmp-1*::*GFP*). RNAi mediated knockdown of *gdi-1* (middle panels) resulted in mis-trafficking of the invadopodial membrane components PI(4,5)_2_, GFP::MIG-2, and GFP::CED-10, as well as GFP::CUP-5 and LMP-1::GFP (which are found both in the invadopodial membrane and the endolysosome) relative to wild type (left panels). RNAi targeting of *cdc-42* did not affect the distribution of PI(4,5)P_2_, GFP::MIG-2, or GFP::CUP-5 (right panels). Box plots (line shows median, boxes cover the interquartile range, and bars show minimum and maximum) display the percentage of the total fluorescent signal at or near the basal invasive cell membrane of the AC. For all conditions a minimum of 9 animals were analyzed (n is noted on each graph). In (A-C) comparisons were made using Tukey’s multiple comparisons tests, ** p < 0.01, *** p < 0.001. In (D-E) comparisons were made using a Student’s t-test, * p < 0.05. Scale bar, 5 μm.
(TIF)

S1 Movie Wild type AC invadopodia dynamics. Ventral view time-lapse showing spot tracking analysis of a wild type animal prior to BM breach. Invadopodia are marked by F-actin (*mCherry*::*moeABD*; left) and with spots (magenta; right) that are overlaid on fluorescence. Isosurface renderings built in place of F-actin were used to determine a threshold for assigning the spots that mark AC invadopodia. Frames were acquired for 24 minutes in one-minute intervals using a spinning disc confocal microscope (CSU-10; Yokogawa Electric Corporation). The frames are played at 10 frames per second. This video corresponds to Figs 1E, 2E, 3D and 4C and Table 2. Scale bar, 5 μm.
(MOV)

S2 Movie AC invadopodia dynamics after loss of *cdc-42*. Ventral view time-lapse showing an AC of a *cdc-42* RNAi treated animal prior to BM breach. Invadopodia are marked by F-actin (*mCherry*::*moeABD*; left) and with spots (magenta; right) that are overlaid on fluorescence. Isosurface renderings built in place of F-actin were used to determine a threshold for assigning the spots that mark AC invadopodia. Loss of *cdc-42* reduced the number invadopodia and decreased the rate of invadopodia formation but did not affect invadopodia lifetimes. Frames were acquired for 24 minutes in one-minute intervals using a spinning disc confocal microscope (CSU-10; Yokogawa Electric Corporation). The frames are played at 10 frames per second. This video corresponds to Fig 1E and 1F and Table 2. Scale bar, 5 μm.
(MOV)

S3 Movie AC invadopodia dynamics after loss of the vulval precursor cells. Ventral view time-lapse showing an AC of a *lin-3* RNAi treated animal prior to BM breach. Invadopodia are marked by F-actin (*mCherry*::*moeABD*; left) and with spots (magenta; right) that are overlaid on fluorescence. Isosurface renderings built in place of F-actin were used to determine a threshold for assigning the spots that mark AC invadopodia. Loss of the vulval precursors cells (via *lin-3* RNAi) and thus loss of a cue(s) generated by the vulval precursor cells reduced the number invadopodia, decreased the rate of invadopodia formation, and increased invadopodia lifetimes. Frames were acquired for 24 minutes in one-minute intervals using a spinning disc confocal microscope (CSU-10; Yokogawa Electric Corporation). The frames are played at 10 frames per second. This video corresponds to Fig 3D and 3E and Table 2. Scale bar, 5 μm.
(MOV)

S4 Movie AC invadopodia dynamics after loss of *gdi-1*. Ventral view time-lapse showing an AC of a *gdi-1* RNAi treated animal prior to BM breach. Invadopodia are marked by F-actin (*mCherry*::*moeABD*; left) and with spots (magenta; right) that are overlaid on fluorescence. Isosurface renderings built in place of F-actin were used to determine a threshold for assigning the spots that mark AC invadopodia. Reduction of *gdi-1* by RNAi reduced the number invadopodia, decreased the rate of invadopodia formation, and increased invadopodia lifetimes. Frames were acquired for 24 minutes in one-minute intervals using a spinning disc confocal microscope (CSU-10; Yokogawa Electric Corporation). The frames are played at 10 frames per second. This video corresponds to Fig 4C and 4E and Table 2. Scale bar, 5 μm.
(MOV)

S5 Movie The AC invadopodial membrane dynamically traffics to the invasive cell membrane. A 3D reconstruction of a lateral view time-lapse of a wild type AC visualizing the invadopodia membrane with a probe for PI(4,5)P2 (cyan; *cdh-3>mCherry*::*PLC*δ^*PH*^) and the basement membrane (magenta; *lam-1>lam-1*::*GFP*). The invadopodial membrane is trafficked dynamically to the invasive membrane throughout the 40-minute time-lapse. The images were acquired in one-minute intervals using a spinning disc confocal microscope (CSU-10; Yokogawa Electric Corporation). The frames are played at 10 frames per second. This video corresponds to Fig 6A. Scale bar, 5 μm.
(MOV)

S6 Movie The invadopodial membrane is mis-trafficked to apical and lateral membranes after loss of *gdi-1*. A 3D reconstruction of a lateral view time-lapse of a *gdi-1* RNAi treated AC visualizing the invadopodial membrane component PI(4,5)P2 (cyan; *cdh-3>mCherry*::*PLC*δ^*PH*^) and the basement membrane (magenta; *lam-1>lam-1*::*GFP*). Loss of *gdi-1* resulted in mis-trafficking of the invadopodial membrane to lateral and apical plasma membrane. The time-lapse takes place over a 40-minute period. The images were acquired in one-minute intervals using a spinning disc confocal microscope (CSU-10; Yokogawa Electric Corporation). The frames are played at 10 frames per second. This video corresponds to Fig 6B. Scale bar, 5 μm.
(MOV)

S7 Movie The invadopodial membrane is mis-trafficked after loss of the vulval precursor cells. A 3D reconstruction of a lateral view time-lapse of a *lin-3* RNAi treated AC visualizing the invadopodial membrane component PI(4,5)P2 (cyan; *cdh-3>mCherry*::*PLC*δ^*PH*^) and the basement membrane (magenta; *lam-1>lam-1*::*GFP*).). *lin-3* RNAi treatment results in loss of the vulval precursor cells, which inhibits generation of the vulval cue. Loss of the vulval precursor cells resulted in mis-trafficking of the invadopodial membrane to the lateral and apical plasma membrane. The time-lapse takes place over a 40-minute period. The images were acquired in one-minute intervals using a spinning disc confocal microscope (CSU-10; Yokogawa Electric Corporation). The frames are played at 10 frames per second. This video corresponds to Fig 6C. Sclae bar, 5 μm.
(MOV)

S8 Movie The invadopodial membrane traffics normally after loss of *cdc-42*. A 3D reconstruction of a lateral view time-lapse of a *cdc-42* RNAi treated AC visualizing the invadopodial membrane component PI(4,5)P2 (cyan; *cdh-3>mCherry*::*PLC*δ^*PH*^) and the basement membrane (magenta; *lam-1>lam-1*::*GFP*). The invadopodial membrane traffics normally to the invasive membrane throughout the 40-minute time-lapse. The images were acquired in one-minute intervals using a spinning disc confocal microscope (CSU-10; Yokogawa Electric Corporation). The frames are played at 10 frames per second. This video corresponds to Fig 6D. Scale bar, 5 μm.
(MOV)
