# Supplementary material for: A Sensitized Screen for Genes Promoting Invadopodia Function In Vivo: CDC-42 and Rab GDI-1 Direct Distinct Aspects of Invadopodia Formation
Source: PLoS Genet. 2016 Jan 14;12(1):e1005786. doi: 10.1371/journal.pgen.1005786 (PMC4713207; doi:10.1371/journal.pgen.1005786)
Supplement: S1 Table — (DOCX) [file pgen.1005786.s001.docx]

**S1 Table. Genome-wide RNAi screen for enhancement of "egl" and "pvl" phenotypes in *unc-40(e271).***

| **Sequence ID** | **Gene Name** | **NCBI KOGs** | **Egg production^a^** | **Protruding vulva^b^** |
| --- | --- | --- | --- | --- |
| Y71G12B.11 | *AC3.5* | Puromycin-sensitive aminopeptidase and related aminopeptidases | (-) | (###) |
| AC8.6 | *AC8.6* | none available | (-) |  |
| B0035.12 | *B0035.12* | RNA-binding protein SART3 | (+) |  |
| B0205.6 | *B0205.6* | Cysteine desulfurase NFS1 | (+) |  |
| B0205.7 | *kin-3* | Casein kinase II, alpha subunit | (+) |  |
| B0207.4 | *air-2* | Serine/threonine protein kinase/chromosome segregation | (-) |  |
| B0222.6 | *col-144* | Collagens (type IV and type XIII), and related proteins | (+) |  |
| B0238.11 | *B0238.11* | HMG box-containing protein /transcriptional regulation | (+) |  |
| B0261.1 | *B0261.1* | Transcription initiation factor TFIIIB, Bdp1 subunit | (-) |  |
| B0261.4 | *B0261.4* | none available | (+) |  |
| B0272.1 | *tbb-4* | Beta tubulin | (-) |  |
| B0273.1 | *B0273.1* | Unnamed protein | (-) |  |
| B0285.1 | *cdtl-7* | Cdc2-related protein kinase | (-) |  |
| B0286.4 | *ntl-2* | Predicted transcriptional regulator | (-) |  |
| B0286.5 | *fkh-6* | Transcription factor of the Forkhead/HNF3 family | (-) |  |
| B0336.6 | *abi-1* | Abl interactor ABI-1, contains SH3 domain | (-) |  |
| B0403.4 | *tag-320* | Thioredoxin/protein disulfide isomerase | (+) |  |
| B0414.5 | *cpb-3* | CPEB orthologous to Drosophila ORB, zebrafish ZORBA, and human CPEB1, and paralogous to FOG-1 and CPB-1/2; CPB-3 inhibits physiological | (-) |  |
| B0464.5 | *spk-1* | Serine/threonine protein kinase/ likekly involved in splicing | (+) |  |
| B0464.7 | *baf-1* | DNA-bridging protein BAF | (-) |  |
| B0491.5 | *B0491.5* | none available | (-) |  |
| B0495.6 | *B0495.6* | Uncharacterized conserved protein | (+) |  |
| B0507.4 | *B0507.4* | Unnamed protein | (+) |  |
| B0511.8 | *tag-264* | Mitochondrial 28S ribosomal protein S30 | (-) |  |
| B0513.7 | *B0513.7* | none available | (-) |  |
| B0554.6 | *dod-20* | Uncharacterized protein(Downstream Of DAF-16 (regulated by DAF-16)) | (+) |  |
| B0563.7 | *B0563.7* | Calmodulin and related proteins (EF-Hand superfamily) | (+) |  |
| B0564.1 | *exos-4.1* | Exosomal 3'-5' exoribonuclease complex, subunit Rrp41 | (-) |  |
| C01B4.9 | *C01B4.9* | Monocarboxylate transporter | (+) |  |
| C01G5.9 | *C01G5.9* | Extracellular protein with conserved cysteines | (+) | (#) |
| C01G8.5 | *erm-1* | Radixin, moesin and related proteins of the ERM family | (-) |  |
| C01G8.9 | *let-526* | SWI-SNF chromatin-remodeling complex protein | (-) | (#) |
| C02B10.5 | *C02B10.5* | none available | (+) |  |
| C02F12.8 | *C02F12.8* | none available | (-) |  |
| C02F5.9 | *pbs-6* | 20S proteasome, regulatory subunit beta type PSMB1/PRE7 | (-) |  |
| C03C10.3 | *rnr-2* | Ribonucleotide reductase, beta subunit | (-) |  |
| C03D6.3 | *cel-1* | mRNA capping enzyme, guanylyltransferase | (-) |  |
| C03G6.15 | *cyp-35A2* | Cytochrome P450 CYP2 subfamily | (+) |  |
| C04C3.3 | *C04C3.3* | Pyruvate dehydrogenase E1, beta subunit | (+) |  |
| C04F5.1 | *sid-1* | sid-1 encodes a transmembrane protein with conserved human (OMIM:606816) and mouse homologs | (-) |  |
| C05C8.3 | *fkb-3* | FKBP-type peptidyl-prolyl cis-trans isomerase | (+) |  |
| C05D11.3 | *tag-170* | ATP binding protein | (+) |  |
| C05E11.3 | *C05E11.3* | none available | (+) |  |
| C05G5.1 | *C05G5.1* | Permease of the major facilitator superfamily | (+) |  |
| C06A1.5 | *rpb-6* | RNA polymerase subunit K | (-) |  |
| C06A8.2 | *C06A8.2* | Small nuclear RNA activating complex | (-) |  |
| C06B3.7 | *C06B3.7* | Unnamed protein | (+) |  |
| C06B8.8 | *rpl-38* | 60S ribosomal protein L38 | (-) |  |
| **C06C3.1** | ***mel-11*** | **Myosin phosphatase, regulatory subunit** | **(+)** |  |
| C06E2.7 | *ubc-22* | Ubiquitin-protein ligase | (+) |  |
| C06E4.6 | *C06E4.6* | Reductases with broad range of substrate specificities | (-) | (##) |
| C06E7.1 | *sams-3* | S-adenosylmethionine synthetase | (-) | (#) |
| C06E7.3 | *sams-4* | S-adenosylmethionine synthetase | (-) | (#) |
| C06G3.10 | *cogc-2* | Low density lipoprotein receptor | (+) |  |
| C07A9.2 | *C07A9.2* | G10 protein/predicted nuclear transcription regulator | (+) |  |
| C07E3.2 | *pro-2* | Predicted protein involved in nuclear export of pre-ribosomes | (+) |  |
| C07H6.1 | *lig-4* | ATP-dependent DNA ligase IV | (-) | (##) |
| C07H6.5 | *cgh-1* | ATP-dependent RNA helicase | (-) |  |
| C08B11.4 | *nrf-6* | Predicted acyltransferase | (+) |  |
| C08B6.9 | *aos-1* | SMT3/SUMO-activating complex, AOS1/RAD31 component | (+) |  |
| C08H9.12 | *C08H9.12* | Chitinase | (+) |  |
| C09G12.8 | *ced-10* | Ras-related small GTPase, Rho type | (+) |  |
| C09H10.7 | *C09H10.7* | none available | (+) |  |
| C10C5.1 | *C10C5.1* | none available | (-) | (##) |
| C10C5.3 | *C10C5.3* | Aminoacylase ACY1 and related metalloexopeptidases | (+) |  |
| C10C6.6 | *C10C6.6* | P-type ATPase | (+) |  |
| C14A11.3 | *cgef-1* | Guanine nucleotide exchange factor for Rho and Rac GTPases | (+) |  |
| C14A4.11 | *C14A4.11* | Putative apoptosis related protein | (-) |  |
| C14A4.5 | *crn-5* | Exosomal 3'-5' exoribonuclease complex, subunit Rrp46 | (+) |  |
| C14B9.4 | *plk-1* | Polo-like serine/threonine protein kinase | (-) |  |
| C14C10.4 | *C14C10.4* | none available | (-) |  |
| C15C8.7 | *C15C8.7* | none available | (+) |  |
| C15F1.4 | *ppp-1* | Translation initiation factor 2B, gamma subunit | (-) |  |
| C15H11.7 | *pas-1* | 20S proteasome, regulatory subunit alpha type PSMA6/SCL1 | (+) |  |
| C15H11.8 | *C15H11.8* | RNA polymerase I transcription factor TFIIS, subunit A12.2/RPA12 | (-) |  |
| C15H11.9 | *rrbs-1* | Regulator of ribosome synthesis | (-) |  |
| C16A11.8 | *clec-135* | C-type lectin | (+) |  |
| C16A3.4 | *C16A3.4* | C2H2-type Zn-finger protein | (+) | (##) |
| C16A3.5 | *C16A3.5* | NADH:ubiquinone oxidoreductase, NDUFB9/B22 subunit | (-) |  |
| C16A3.6 | *C16A3.6* | RNA-binding nuclear protein | (-) |  |
| C17E4.5 | *pabp-2* | Splicing factor RNPS1, SR protein superfamily | (+) |  |
| C17E4.9 | *nkb-1* | Na+/K+ ATPase, beta subunit | (+) |  |
| C17E7.8 | *nhr-124* | Nuclear hormone receptor | (+) |  |
| C17G10.2 | *C17G10.2* | Hsp90 co-chaperone CNS1 | (+) |  |
| C18D11.4 | *rsp-8* | RRM domain/ SR Protein (splicing factor) | (-) |  |
| C23G10.8 | *C23G10.8* | Unnamed protein | (+) | (##) |
| C23H3.2 | *C23H3.2* | none available | (+) |  |
| C24D10.4 | *C24D10.4* | none available | (+) | (#) |
| C24G6.6 | *C24G6.6* | Flavin-containing amine oxidase | (+) |  |
| C25D7.6 | *mcm-3* | DNA replication licensing factor | (+) |  |
| C25F9.4 | *C25F9.4* | Helicase-like transcription factor HLTF/DNA helicase RAD5, DEAD-box superfamily | (+) |  |
| C26C6.5 | *dcp-66* | Uncharacterized conserved protein | (-) | (#) |
| C26D10.1 | *ran-3* | RCC1 domain | (-) | (###) |
| C27B7.6 | *C27B7.6* | Serine/threonine specific protein phosphatase PP1, catalytic subunit | (+) |  |
| C27H6.2 | *ruvb-1* | DNA helicase, TBP-interacting protein | (-) |  |
| C28A5.4 | *ceh-43* | Transcription factor DLX and related proteins with LIM Zn-binding and HOX | (+) |  |
| C29E6.3 | *pph-2* | Protein tyrosine phosphatase | (+) |  |
| C29F3.4 | *clec-231* | C-type lectin | (+) |  |
| C29F7.2 | *C29F7.2* | Predicted small molecule kinase | (+) |  |
| **C29F9.7** | ***pat-4*** | **Integrin-linked kinase** | **(-)** |  |
| C30C11.4 | *C30C11.4* | Molecular chaperones HSP105/HSP110/SSE1, HSP70 superfamily | (-) |  |
| C30H6.2 | *tag-141* | Putative zinc transporter | (+) |  |
| C30H6.7 | *C30H6.7* | Dihydrolipoamide acetyltransferase | (-) |  |
| C31B8.12 | *C31B8.12* | Predicted secreted small molecules methylase | (+) |  |
| C31H2.2 | *dpy-8* | Collagens (type IV and type XIII), and related proteins | (-) |  |
| C32A3.1 | *sel-8* | Unnamed protein | (+) |  |
| C33A12.14 | *sru-1* | Serpentine Receptor, class U | (+) |  |
| C33E10.1 | *C33E10.1* | protein-protein interactions either with homologs of yeast Skp-1p or with other proteins | (+) |  |
| C33G8.4 | *C33G8.4* | Uncharacterized protein | (+) |  |
| C33H5.15 | *sgo-1* | none available | (+) |  |
| C33H5.7 | *swd-2.2* | Histone H3 (Lys4) methyltransferase complex and RNA cleavage factor II | (-) | (#) |
| C34B2.10 | *C34B2.10* | Signal peptidase subunit | (-) |  |
| C34B2.8 | *C34B2.8* | NADH:ubiquinone oxidoreductase | (-) |  |
| C34D4.8 | *str-48* | 7-transmembrane olfactory receptor | (+) | (#) |
| C34F6.7 | *C34F6.7* | Uncharacterized conserved protein | (+) |  |
| C35A5.7 | *C35A5.7* | Unnamed protein (probable g-protein coupled receptor) | (+) |  |
| C35A5.8 | *C35A5.8* | none available | (-) |  |
| C36B1.5 | *prp-4* | U4/U6 small nuclear ribonucleoprotein Prp4 | (-) |  |
| C36E6.5 | *mlc-2* | Myosin regulatory light chain, EF-Hand protein superfamily | (+) |  |
| C36E8.1 | *C36E8.1* | RNA polymerase I transcription factor | (+) |  |
| C37A2.4 | *cye-1* | G1/S-specific cyclin E | (+) |  |
| C37C3.10 | *C37C3.10* | Unnamed protein | (+) |  |
| **C37C3.6** | ***mig-6*** | **Serine proteinase inhibitor (KU family) with thrombospondin repeats** | **(-)** | **(##)** |
| C38C10.3 | *C38C10.3* | Unnamed protein | (+) |  |
| **C38C10.4** | ***gpr-2*** | **exchange factors specific for G-alpha GTPases** | **(-)** |  |
| C38C3.5 | *unc-60* | Actin depolymerizing factor | (-) | (###) |
| C39E9.10 | *C39E9.10* | Sugar transporter/spinster transmembrane protein | (+) |  |
| C39E9.14 | *dli-1* | Dynein light intermediate chain | (-) | (#) |
| C41G6.1 | *cyp-34A3* | Cytochrome P450 CYP2 subfamily | (+) |  |
| C41G7.1 | *smn-1* | mRNA splicing protein SMN | (+) |  |
| C42C1.5 | *tag-335* | Temporarily Assigned Gene name) | (-) | (#) |
| C43E11.9 | *C43E11.9* | Ribosome biogenesis protein NIP7 | (-) | (##) |
| C44C1.4 | *vps-45* | Vacuolar sorting protein VPS45/Stt10 | (+) |  |
| C44C10.3 | *C44C10.3* | Synaptic vesicle transporter SVOP and related transporters | (+) |  |
| C47A4.2 | *C47A4.2* | Nucleoside transporter | (+) |  |
| C47B2.5 | *eif-6* | Translation initiation factor 6 | (-) |  |
| C47D12.2 | *C47D12.2* | Proteins containing regions of low-complexity | (+) |  |
| C47E12.2 | *C47E12.2* | Mitochondrial ADP/ATP carrier proteins | (-) |  |
| C47E12.7 | *C47E12.7* | Nucleolar protein NOP52/RRP1 | (+) |  |
| C47E8.7 | *unc-112* | Mitogen inducible gene product (Integrin pathway) | (-) |  |
| C48B6.2 | *C48B6.2* | U3 small nucleolar ribonucleoprotein | (+) |  |
| C48E7.2 | *C48E7.2* | RNA polymerase III (C) subunit | (-) |  |
| C49F5.1 | *sams-1* | S-adenosylmethionine synthetase | (+) |  |
| C49G7.6 | *C49G7.6* | none available | (-) |  |
| C50E3.10 | *scl-26* | Defense-related protein containing SCP domain | (+) |  |
| C52E4.6 | *cyl-1* | CYclin L) | (-) | (#) |
| C53A5.3 | *hda-1* | Histone deacetylase complex, catalytic component RPD3 | (-) |  |
| C53B7.1 | *rig-3* | Immunoglobulin C-2 Type/fibronectin type III domains | (+) |  |
| C53B7.4 | *asg-2* | Mitochondrial F1F0-ATP synthase, subunit g/ATP20 | (-) |  |
| C54C6.1 | *rpl-37* | 60S ribosomal protein L37 | (+) |  |
| C54G10.3 | *pmp-3* | Peroxisomal Membrane Protein related | (-) | (##) |
| C55A6.9 | *C55A6.9* | Putative RNA polymerase II regulator | (-) |  |
| C55B6.2 | *dnj-7* | dsRNA-activated protein kinase inhibitor P58, contains TPR and DnaJ domains | (+) |  |
| C55B7.5 | *uri-1* | Uncharacterized conserved protein | (-) |  |
| C56C10.8 | *icd-1* | RNA polymerase II general transcription factor BTF3 and related proteins | (-) |  |
| C56G2.3 | *C56G2.3* | Uncharacterized conserved protein | (+) |  |
| CD4.3 | *CD4.3* | Uncharacterized conserved protein | (+) |  |
| D1007.7 | *nrd-1* | RNA polymerase II C-terminal domain-binding protein | (-) |  |
| D1009.3 | *D1009.3* | Ficolin and related extracellular proteins | (+) |  |
| D1043.1 | *D1043.1* | Uncharacterized conserved protein | (-) | (###) |
| D1054.13 | *secs-1* | Predicted serine hydroxymethyltransferase SLA/LP | (+) |  |
| D1054.3 | *D1054.3* | Suppressor of G2 allele of skp1 | (+) |  |
| D2013.6 | *D2013.6* | WW domain binding protein WBP-2, contains GRAM domain | (-) |  |
| D2024.3 | *elo-3* | Long chain fatty acid elongase | (+) |  |
| D2030.3 | *D2030.3* | none available | (-) | (#) |
| DY3.2 | *lmn-1* | Nuclear envelope protein lamin | (+) |  |
| E01A2.7 | *E01A2.7* | Unnamed protein | (+) |  |
| E02A10.1 | *mrps-5* | Ribosomal protein S5 | (-) |  |
| E02H1.1 | *E02H1.1* | Ribosomal RNA adenine dimethylase | (+) | (#) |
| E03E2.1 | *cyp-43A1* | Cytochrome P450 CYP3/CYP5/CYP6/CYP9 subfamilies | (+) |  |
| E03G2.4 | *col-186* | Collagens (type IV and type XIII), and related proteins | (+) |  |
| E04A4.7 | *cyc-2.1* | Cytochrome c | (+) |  |
| EEED8.1 | *mel-47* | none available | (+) |  |
| EEED8.5 | *mog-5* | DEAH-box RNA helicase | (+) |  |
| F01E11.1 | *ugt-57* | UDP-glucuronosyl and UDP-glucosyl transferase | (+) |  |
| F01G10.1 | *tkt-1* | Transketolase | (+) |  |
| F01G12.6 | *F01G12.6* | none available | (+) |  |
| F01G4.6 | *F01G4.6* | Mitochondrial phosphate carrier protein | (-) |  |
| F02C12.5 | *cyp-13B1* | Cytochrome P450 CYP3/CYP5/CYP6/CYP9 subfamilies | (+) |  |
| F07A11.2 | *F07A11.2* | Glucosamine 6-phosphate synthetases, contain amidotransferase | (-) |  |
| F08B4.1 | *dic-1* | human DICE1 (Deleted In Cancer) homolog) | (+) |  |
| F08B6.4 | *unc-87* | unc-87 encodes, through alternative splicing, two proteins that are required to maintain the structure of myofilaments in body wall muscle cells | (-) |  |
| F08G12.2 | *F08G12.2* | U5 snRNP-specific protein-like factor and related proteins | (+) |  |
| **F09A5.4** | ***F09A5.4*** | **Cell cycle-associated protein Mob1-1** | **(+)** |  |
| F09C8.1 | *F09C8.1* | Phospholipase | (+) |  |
| F09D1.1 | *F09D1.1* | Spindle pole body protein | (-) | (#) |
| F09E5.1 | *pkc-3* | Serine/threonine protein kinase | (+) |  |
| F09F7.2 | *mlc-3* | Myosin essential light chain, EF-Hand protein | (-) |  |
| F09F9.4 | *F09F9.4* | Unnamed protein | (-) |  |
| F09G8.3 | *F09G8.3* | Mitochondrial/chloroplast ribosomal protein S9 | (-) |  |
| F10B5.3 | *F10B5.3* | Zn-finger | (+) |  |
| F10B5.6 | *emb-27* | Anaphase-promoting complex | (-) | (##) |
| F10C2.6 | *dars-2* | Aspartyl-tRNA synthetase, mitochondrial | (-) | (#) |
| F10C5.1 | *mat-3* | Anaphase-promoting complex (APC), Cdc23 subunit | (+) |  |
| F10D2.9 | *fat-7* | Fatty acid desaturase | (+) |  |
| F10D7.2 | *F10D7.2* | Permease of the major facilitator superfamily | (+) |  |
| F10G7.4 | *scc-1* | Sister chromatid cohesion complex Cohesin | (-) | (##) |
| F10G8.3 | *npp-17* | mRNA export protein (contains WD40 repeats) | (-) |  |
| F11A3.2 | *F11A3.2* | Translation initiation factor 2B, delta subunit | (+) |  |
| F11G11.8 | *nspd-5* | Unnamed protein | (+) |  |
| F12E12.7 | *sdz-11* | sdz-11 - (SKN-1 Dependent Zygotic transcript) | (+) | (##) |
| F12F6.7 | *F12F6.7* | DNA polymerase delta, regulatory subunit 55 | (-) |  |
| F13E6.2 | *F13E6.2* | Uridylate kinase/adenylate kinase | (-) |  |
| F14B4.3 | *F14B4.3* | RNA polymerase I, second largest subunit | (-) | (#) |
| F15A4.5 | *F15A4.5* | no/ 7 TM Receptor | (-) |  |
| F15D3.7 | *F15D3.7* | Mitochondrial import inner membrane translocase, subunit TIM23 | (-) |  |
| F16B4.7 | *F16B4.7* | Unnamed protein | (+) |  |
| F16B4.8 | *cdc-25.2* | M-phase inducer phosphatase | (-) |  |
| F17C11.2 | *F17C11.2* | Unnamed protein | (+) |  |
| F17H10.1 | *F17H10.1* | Uncharacterized conserved protein | (+) |  |
| F18E2.3 | *scc-3* | Sister chromatid cohesion complex Cohesin, subunit STAG/IRR1/SCC3 | (-) | (#) |
| F18G5.3 | *gpa-12* | G-protein alpha subunit | (+) |  |
| F19B6.1 | *F19B6.1* | Armadillo/beta-Catenin/plakoglobin | (-) | (##) |
| F19C7.7 | *col-110* | Collagens (type IV and type XIII), and related proteins | (-) |  |
| F20D12.1 | *csr-1* | Translation initiation factor 2C (eIF-2C) and related proteins | (+) |  |
| F20D12.2 | *F20D12.2* | Nuclear protein export factor | (-) | (#) |
| F20H11.3 | *mdh-1* | NAD-dependent malate dehydrogenase | (-) |  |
| F20H11.6 | *F20H11.6* | none available | (+) |  |
| F21C3.5 | *pfd-6* | Prefoldin subunit 6 | (+) |  |
| **F22B7.13** | ***gpr-1*** | **tetratricopeptide-like motifs and a C-terminal GoLoco/GPR (G protein regulatory) motif** | **(+)** | **(#)** |
| F22D6.5 | *prpf-4* | U4/U6-associated splicing factor PRP4 | (-) | (#) |
| F23B12.5 | *F23B12.5* | Dihydrolipoamide acetyltransferase | (-) |  |
| F23B12.6 | *fntb-1* | Beta subunit of farnesyltransferase | (+) |  |
| F23B12.7 | *F23B12.7* | CAATT-binding transcription factor/60S ribosomal subunit biogenesis protein | (-) | (#) |
| F23B2.13 | *rpb-12* | DNA-directed RNA polymerase, subunit RPB7.0 | (+) | (#) |
| F23B2.3 | *F23B2.3* | Non voltage-gated ion channels | (+) | (#) |
| F23F1.5 | *F23F1.5* | m3G-cap-specific nuclear import receptor | (+) |  |
| F23H12.2 | *tomm-20* | Translocase of outer mitochondrial membrane complex, subunit TOM20 | (-) |  |
| F25B3.6 | *F25B3.6* | Paf1/RNA polymerase II complex, RTF1 component | (-) | (#) |
| F25B4.9 | *clec-1* | C-type lectin | (+) |  |
| **F25E2.1** | ***gtr-1*** | **none available** | **(+)** |  |
| F25H5.6 | *F25H5.6* | Mitochondrial/chloroplast ribosomal protein L54/L37 | (+) |  |
| F25H8.1 | *F25H8.1* | Uncharacterized conserved protein | (+) | (#) |
| F25H8.2 | *F25H8.2* | Uncharacterized conserved protein | (+) |  |
| F25H8.6 | *bed-3* | Zinc finger protein,Tam3-transposase | (+) | (#) |
| F26A10.2 | *F26A10.2* | Zn-finger | (-) | (#) |
| F26A3.2 | *ncbp-2* | Nuclear cap-binding protein complex, subunit | (-) |  |
| F26E4.1 | *sur-6* | Serine/threonine protein phosphatase 2A, regulatory subunit | (+) |  |
| F26E4.9 | *cco-1* | Cytochrome c oxidase, subunit Vb | (-) |  |
| F26F4.11 | *rpb-8* | RNA polymerase subunit 8 | (-) |  |
| F27C1.6 | *F27C1.6* | Uncharacterized conserved protein | (-) | (#) |
| F27D4.2 | *lsy-22* | none available | (+) |  |
| F27D9.6 | *dhs-29* | Hydroxysteroid 17-beta dehydrogenase 11 | (+) |  |
| F27E5.2 | *pax-3* | Transcription factor PRD and related proteins, contain PAX and HOX domains | (+) |  |
| F28C6.6 | *suf-1* | mRNA cleavage and polyadenylation factor | (-) |  |
| F28D1.1 | *F28D1.1* | WD40-repeat-containing subunit of the 18S rRNA processing complex | (-) |  |
| F29C12.4 | *F29C12.4* | Mitochondrial elongation factor | (+) |  |
| F29F11.6 | *gsp-1* | Serine/threonine specific protein phosphatase PP1, catalytic subunit | (-) | (##) |
| F29G6.2 | *F29G6.2* | Uncharacterized coiled-coil protein | (+) |  |
| F30F8.2 | *glna-3* | Glutaminase (contains ankyrin repeat | (+) |  |
| F30F8.8 | *taf-5* | none available | (-) | (###) |
| F31D4.3 | *fkb-6* | FKBP-type peptidyl-prolyl cis-trans isomerase | (+) |  |
| F31F6.6 | *nac-1* | Na+/dicarboxylate, Na+/tricarboxylate and phosphate transporters | (-) |  |
| F32D1.10 | *mcm-7* | DNA replication licensing factor | (-) |  |
| F32D1.8 | *F32D1.8* | none available | (-) |  |
| F32D8.5 | *F32D8.5* | Uncharacterized membrane protein | (-) |  |
| F32E10.1 | *nol-10* | WD40 repeat protein /nucleolar | (-) |  |
| F32H2.1 | *gei-11* | Transcription factor, Myb superfamily | (-) |  |
| F32H2.3 | *spd-2* | essential for centrosome maturation and duplication, | (-) |  |
| F33D4.1 | *nhr-8* | Hormone receptors | (+) |  |
| F35A5.3 | *abu-10* | abu-10 - (Activated in Blocked Unfolded protein response | (+) |  |
| F35E2.9 | *F35E2.9* | none available | (+) |  |
| F35F11.1 | *F35F11.1* | RNA polymerase II assessory factor Cdc73p | (+) |  |
| F35H12.4 | *F35H12.4* | (PI 4-Kinase) | (+) |  |
| F36A4.10 | *col-34* | Collagens (type IV and type XIII), and related proteins | (+) |  |
| F36A4.6 | *col-33* | Collagens (type IV and type XIII), and related proteins | (+) |  |
| F36F12.8 | *ztf-28* | Zn-finger | (+) |  |
| F36G3.2 | *F36G3.2* | Uncharacterized protein | (-) |  |
| **F36H1.4** | ***lin-3*** | **lin-3 encodes a member of the EGF family of peptide growth factors** | **(-)** |  |
| F37E3.1 | *ncbp-1* | Nuclear cap-binding complex | (-) | (#) |
| F38H4.4 | *F38H4.4* | Protein tyrosine phosphatase | (+) |  |
| F39B2.10 | *dnj-12* | Molecular chaperone | (-) |  |
| F39C12.3 | *tsp-14* | Tetraspanin family integral membrane protein | (+) |  |
| F39H2.2 | *sig-7* | Predicted peptidyl prolyl cis-trans isomerase | (+) |  |
| F40C5.1 | *F40C5.1* | none available | (+) |  |
| F40F4.1 | *fbxb-71* | F-box B protein,Predicted transposase | (+) |  |
| F40F4.6 | *F40F4.6* | (EGF Repeats) | (-) |  |
| F40G9.3 | *ubc-20* | Ubiquitin-protein ligase | (+) |  |
| F41B4.4 | *glr-6* | Glutamate-gated kainate-type ion channel receptor subunit | (+) |  |
| F41B5.2 | *cyp-33C7* | CYtochrome P450 family | (+) |  |
| F41C3.4 | *F41C3.4* | contains similarity to Pfam domain PF04178 Got1-like family | (+) |  |
| F41E6.4 | *smk-1* | Protein required for actin cytoskeleton organization and cell cycle progression | (+) |  |
| F42A6.7 | *hrp-1* | RRM domain | (+) | (##) |
| F42A8.2 | *sdhb-1* | Succinate dehydrogenase, Fe-S protein subunit | (-) |  |
| F42C5.2 | *F42C5.2* | 7 transmembrane receptor | (+) |  |
| F42D1.2 | *tatn-1* | Tyrosine aminotransferase | (-) |  |
| F42G8.11 | *sph-1* | MARVEL domain, a membrane-associating domain found in lipid-associating proteins, and contains a transmembrane domain | (-) |  |
| F43D2.3 | *F43D2.3* | Unnamed protein | (+) |  |
| F43G9.1 | *F43G9.1* | Isocitrate dehydrogenase, alpha subunit | (-) |  |
| F43G9.10 | *mfap-1* | Microfibrillar-associated protein MFAP1 | (+) |  |
| F43G9.12 | *F43G9.12* | Transcriptional regulators binding to the GC-rich sequences [ | (-) |  |
| F44A2.2 | *F44A2.2* | Voltage-gated K+ channel KCNB/KCNC | (+) |  |
| F44A6.2 | *sex-1* | Nuclear receptor | (+) |  |
| F44C4.4 | *gon-14* | abnormal GONad development | (+) |  |
| F44F4.2 | *egg-3* | Alternative splicing factor SRp55/B52/SRp75 | (-) |  |
| F44G4.2 | *F44G4.2* | contains similarity to Homo sapiens NADH-ubiquinone oxidoreductase | (-) |  |
| F45E10.2 | *F45E10.2* | Synaptic vesicle transporter SVOP and related transporters | (-) | (#) |
| F45E4.9 | *hmg-5* | HMG box-containing protein | (+) | (#) |
| F45H10.3 | *F45H10.3* | NADH:ubiquinone oxidoreductase | (+) |  |
| F46A9.5 | *skr-1* | SCF ubiquitin ligase, Skp1 component | (+) |  |
| F46C8.5 | *ceh-14* | Transcription factor LIM3, contains LIM and HOX domains | (+) |  |
| F46C8.6 | *dpy-7* | Collagens (type IV and type XIII), and related proteins | (+) |  |
| F47D12.4 | *hmg-1.2* | HMG box-containing protein | (-) |  |
| F48G7.12 | *F48G7.12* | Predicted small molecule kinase | (+) |  |
| F49C12.2 | *F49C12.2* | Predicted secreted small molecules methylase | (+) |  |
| F49C12.4 | *F49C12.4* | Predicted secreted small molecules methylase | (+) |  |
| F49E11.7 | *F49E11.7* | no/ Serine/threonine-protein phosphatase | (+) |  |
| F52C12.5 | *elt-6* | GATA-4/5/6 transcription factors | (-) |  |
| F52E4.6 | *wrt-2* | wrt-2 encodes a hedgehog-like protein | (+) |  |
| F52F10.1 | *F52F10.1* | none available | (+) |  |
| F52H2.2 | *aat-3* | Amino acid transporters | (+) |  |
| F52H3.1 | *let-268* | Lysyl hydroxylase | (+) |  |
| F52H3.2 | *F52H3.2* | NAD/FAD-utilizing protein possibly involved in translation | (+) |  |
| F53A9.6 | *F53A9.6* | Unnamed protein | (+) |  |
| F53B7.3 | *F53B7.3* | mRNA splicing factor | (+) |  |
| F53E4.1 | *F53E4.1* | Uncharacterized conserved protein, contains kelch repeat | (-) |  |
| F53F4.11 | *F53F4.11* | Uncharacterized conserved protein | (-) | (##) |
| F53G2.7 | *mnat-1* | Predicted E3 ubiquitin ligase containing RING finger, subunit of transcription/repair factor | (-) |  |
| F54C4.2 | *spt-4* | Transcription elongation factor SPT4 | (-) |  |
| F54C8.4 | *F54C8.4* | mRNA capping enzyme, guanylyltransferase (alpha) subunit | (+) |  |
| F54C9.1 | *iff-2* | Translation initiation factor 5A | (-) | (##) |
| F54C9.2 | *stc-1* | Molecular chaperones HSP70/HSC70, HSP70 superfamily | (-) |  |
| F54C9.6 | *bcs-1* | AAA+-type ATPase | (-) |  |
| F54C9.9 | *F54C9.9* | KRR1-interacting protein involved in 40S ribosome biogenesis | (+) |  |
| F54D10.3 | *F54D10.3* | Uncharacterized protein | (+) | (#) |
| F54D5.5 | *F54D5.5* | Unnamed protein | (-) |  |
| F55B12.4 | *F55B12.4* | tRNA nucleotidyltransferase/poly(A) polymerase | (-) | (##) |
| F55C5.5 | *tsfm-1* | Mitochondrial translation elongation factor EF-Tsmt, catalyzes nucleotide | (+) |  |
| F55D10.3 | *glit-1* | Carboxylesterase and related proteins | (+) |  |
| F55F3.3 | *nkb-3* | Na+/K+ ATPase, beta subunit | (+) |  |
| F55F8.2 | *F55F8.2* | RNA helicase | (-) |  |
| F55F8.5 | *tag-345* | Microtubule binding protein YTM1 | (-) |  |
| F56A3.4 | *spd-5* | none available | (+) |  |
| F56A8.6 | *cpf-2* | mRNA cleavage and polyadenylation factor I complex, subunit RNA15 | (-) |  |
| F56B3.8 | *F56B3.8* | Mitochondrial/chloroplast ribosomal protein L2 | (-) |  |
| F56B6.4 | *gyg-1* | Glycosyl transferase, family 8 | (-) |  |
| F56D2.6 | *F56D2.6* | mRNA splicing factor ATP-dependent RNA helicase | (+) | (#) |
| F56D5.10 | *srxa-2* | Serpentine Receptor, class XA | (+) |  |
| F56F3.1 | *pqn-45* | none available | (+) |  |
| F56G4.4 | *F56G4.4* | Spliceosomal protein FBP21 | (+) | (#) |
| F57B1.2 | *sun-1* | Spindle pole body protein, contains UNC-84 domain | (+) |  |
| F57B9.5 | *byn-1* | Cell adhesion complex protein bystin | (-) |  |
| F57G4.4 | *fbxa-191* | protein-protein interactions either with homologs of yeast Skp-1p or with other proteins; | (+) |  |
| F57H12.3 | *ora-1* | Onchocerca Related Antigen family | (+) |  |
| F58A4.3 | *hcp-3* | Histones H3 and H4 | (+) |  |
| F58A4.4 | *pri-1* | Eukaryotic-type DNA primase, catalytic (small) subunit | (-) |  |
| F58A4.8 | *tbg-1* | Gamma tubulin | (-) |  |
| F58B3.5 | *mars-1* | Methionyl-tRNA synthetase | (+) |  |
| F58B3.9 | *ttr-50* | Uncharacterized protein with conserved cysteine | (+) |  |
| F58D5.1 | *hrp-2* | Heterogeneous nuclear ribonucleoprotein R | (-) | (##) |
| F58E10.2 | *end-1* | GATA-4/5/6 transcription factors | (-) |  |
| F58G11.1 | *letm-1* | Ca2+-binding transmembrane protein/ mitochondrial | (+) |  |
| F58G6.4 | *acc-1* | Ligand-gated ion channel | (+) |  |
| F59A2.1 | *npp-9* | Ran-binding protein RANBP1 and related RanBD domain proteins | (-) | (##) |
| F59A2.4 | *clpf-1* | mRNA cleavage and polyadenylation factor IA/II complex, subunit CLP1 | (+) |  |
| F59A3.3 | *F59A3.3* | Mitochondrial/chloroplast ribosomal protein L24 | (+) |  |
| F59C6.5 | *F59C6.5* | NADH-ubiquinone oxidoreductase, subunit NDUFB10/PDSW | (-) |  |
| F59E12.11 | *F59E12.11* | Uncharacterized conserved protein | (+) |  |
| F59F5.1 | *F59F5.1* | none available | (-) | (#) |
| H01G02.2 | *H01G02.2* | Serine/Threonine protein kinases,catalytic domain | (+) |  |
| H06H21.3 | *eif-1.A* | Translation initiation factor 1A (eIF-1A) | (-) |  |
| H08M01.1 | *H08M01.1* | Unnamed protein | (+) |  |
| H12C20.2 | *pms-2* | DNA mismatch repair protein | (+) |  |
| H14N18.3 | *ttr-47* | Uncharacterized protein with conserved cysteine | (+) |  |
| H18N23.2 | *H18N23.2* | Protein phosphatase, regulatory subunit PPP1R3C/D | (-) |  |
| H20J04.4 | *H20J04.4* | ATP-dependent RNA helicase | (+) |  |
| H22K11.1 | *asp-3* | Aspartyl protease | (+) |  |
| H24O09.1 | *srbc-40* | Serpentine Receptor, class BC (class B-like) | (+) |  |
| H26D21.2 | *msh-2* | Mismatch repair ATPase | (+) |  |
| H35B03.2 | *H35B03.2* | Protein subunit of nuclear ribonuclease P (RNase P) | (-) |  |
| K01C8.6 | *K01C8.6* | Mitochondrial ribosomal protein L10 | (-) |  |
| K01C8.9 | *nst-1* | GTPase | (+) |  |
| K01H12.3 | *nhr-38* | C4-type Zn-finger | (-) |  |
| K02A11.1 | *gfi-2* | Myosin phosphatase, regulatory subunit | (+) |  |
| K02B2.4 | *inx-7* | Innexin-type channels | (+) | (#) |
| K02E11.3 | *K02E11.3* | Unnamed protein | (-) |  |
| K02F2.3 | *teg-4* | Splicing factor 3b, subunit 3 | (+) |  |
| K03A1.2 | *K03A1.2* | Leucine rich repeat | (+) |  |
| K04C2.2 | *K04C2.2* | Predicted regulator of rRNA gene transcription | (+) |  |
| K04D7.1 | *rack-1* | G protein beta subunit-like protein/rack-1: Receptor of Activated C Kinase | (+) |  |
| K04D7.5 | *gon-4* | nuclear protein directly required for gonadogenesis in both sexes | (+) |  |
| K04G7.11 | *K04G7.11* | none available | (-) |  |
| K06C4.6 | *mod-1* | Ligand-gated ion channe | (+) |  |
| K06H7.6 | *apc-2* | a cyclin-specific E3 RING ubiquitin ligase | (+) |  |
| K07A1.11 | *rba-1* | Nucleosome remodeling factor, subunit | (-) |  |
| K07A1.2 | *dut-1* | dUTPase | (-) |  |
| K07A12.3 | *asg-1* | Mitochondrial F1F0-ATP synthase, subunit g/ATP20 | (-) |  |
| K07B1.3 | *ucp-4* | Mitochondrial fatty acid anion carrier protein/Uncoupling protein | (+) |  |
| K07C11.2 | *air-1* | Serine/threonine protein kinase/AIR-1 centrosomal gamma-tubulin | (-) |  |
| K07C11.3 | *K07C11.3* | Metalloproteinase inhibitor TIMP and related proteins | (+) |  |
| K07C5.4 | *K07C5.4* | Ribosome biogenesis protein - Nop56p/Sik1p | (-) |  |
| K07C5.6 | *K07C5.6* | RNA splicing factor - Slu7p | (+) |  |
| K07H8.1 | *K07H8.1* | Beta-tubulin folding cofactor E | (-) |  |
| K07H8.3 | *K07H8.3* | Subunit of the major N alpha-acetyltransferase | (-) |  |
| K08A8.2 | *sox-2* | HMG-box transcription factor | (-) | (###) |
| K08B4.1 | *lag-1* | lag-1/CSL Recombination signal binding protein-J kappa | (-) | (##) |
| K08B5.1 | *K08B5.1* | none available | (+) |  |
| K08E3.6 | *cyk-4* | GTPase-activating protein | (-) |  |
| K08F9.4 | *K08F9.4* | none available | (-) |  |
| K09A9.5 | *gas-1* | NADH:ubiquinone oxidoreductase, NDUFS2/49 kDa subunit | (+) |  |
| K09B11.2 | *nol-9* | Uncharacterized conserved protein similar to ATP/GTP-binding protein | (-) |  |
| K09E10.2 | *oac-58* | Integral membrane O-acyltransferase | (+) |  |
| K09H9.6 | *lpd-6* | RNA-binding protein required for 60S ribosomal | (-) | (#) |
| K10C2.4 | *fah-1* | Fumarylacetoacetase | (+) |  |
| K10D3.2 | *unc-14* | RUN domain containing protein required for axonal guidance | (-) |  |
| K10G6.1 | *lin-31* | Forkhead/HNF-3-related transcription factor | (+) |  |
| K12B6.3 | *fil-2* | Triacylglycerol lipase | (+) |  |
| K12B6.4 | *K12B6.4* | none available | (+) |  |
| K12C11.2 | *smo-1* | Ubiquitin-like proteins | (+) |  |
| M01D7.7 | *egl-30* | G protein subunit Galphaq/Galphay, small G protein superfamily (egl-30) | (-) |  |
| M03D4.1 |  | Kinesin-like protein/zen-4 completion of cytokinesis | (-) |  |
| M03F4.6 | zen-4 | Proteins containing Ca2+-binding EGF-like domains | (+) |  |
| M03F4.7 | M03F4.6 | Reticulocalbin, calumenin, DNA supercoiling factor, and related Ca2+-binding proteins | (+) |  |
| M05B5.5 | calu-1 | Helix loop helix transcription factor | (-) |  |
| M106.1 | hlh-2 | Structural maintenance of chromosome protein 2 | (-) | (##) |
| M106.5 | mix-1 | F-actin capping protein, beta subunit | (-) |  |
| M116.5 | cap-2 | Dystonin, GAS (Growth-arrest-specific protein), and related proteins (MT-Actin linker) | (+) |  |
| M142.4 | M116.5 | Transcription factor EVX1, contains HOX domain | (+) |  |
| M18.5 | vab-7 | Damage-specific DNA binding complex, subunit DDB1 | (-) |  |
| M60.2 | ddb-1 | Placental protein 11 | (+) |  |
| M7.1 | M60.2 | Ubiquitin-protein ligase, | (-) | (##) |
| M7.5 | let-70 | Ubiquitin activating E1 enzyme-like protein | (+) |  |
| R02D3.3 | atg-7 | RNA polymerase II transcription initiation/nucleotide excision repair factor | (+) | (#) |
| R02F2.7 | R02D3.3 | none available | (-) |  |
| R03C1.3 | R02F2.7 | Transcription factor, contains HOX domain | (-) | (##) |
| R03G8.6 | cog-1 | Puromycin-sensitive aminopeptidase and related aminopeptidases | (-) |  |
| R04B3.2 | R03G8.6 | Asparaginase | (-) |  |
| R04F11.2 | R04B3.2 | Mitochondrial F1F0-ATP synthase, subunit e | (-) |  |
| R05A10.5 | R04F11.2 | Zinc metalloprotein | (+) |  |
| R05D3.11 | R05A10.5 | none available | (+) |  |
| R05G6.7 | met-2 | Porin/voltage-dependent anion-selective channel protein | (-) |  |
| R05H10.2 | R05G6.7 | Nucleolar protein fibrillarin NOP77 (RRM superfamily) | (-) |  |
| **R06A10.2** | **gsa-1** | **G protein subunit Galphas** | **(+)** |  |
| R06A4.4 | imb-2 | Nuclear transport receptor Karyopherin-beta2/Transportin | (+) |  |
| R06C1.2 | R06C1.2 | Polyprenyl synthetase | (+) | (##) |
| R06C7.5 | R06C7.5 | Adenylosuccinate lyase | (+) | (#) |
| R06C7.8 | bub-1 | Mitotic checkpoint serine/threonine protein kinase | (-) | (###) |
| R06F6.1 | cdl-1 | Histone mRNA stem-loop binding protein | (-) | (###) |
| R07E4.4 | mig-23 | Nucleoside phosphatase | (+) |  |
| R07E5.3 | snfc-5 | SWI-SNF chromatin remodeling complex, Snf5 subunit | (-) |  |
| **R07G3.1** | **cdc-42** | **Ras-related small GTPase, Rho type** | **(-)** |  |
| R07G3.5 | pgam-5 | Predicted phosphoglycerate mutase | (+) |  |
| R08C7.2 | R08C7.2 | Cell cycle control protein | (+) |  |
| R08C7.4 | R08C7.4 | Extracellular protein with conserved cysteines | (-) | (#) |
| R08D7.1 | R08D7.1 | Uncharacterized conserved protein | (+) |  |
| R09B3.5 | mag-1 | Exon-exon junction complex, Magoh component | (-) |  |
| R09G11.2 | nhr-1 | nhr-1 encodes a member of the nuclear receptor superfamily | (-) |  |
| R10H10.1 | lpd-8 | NifU-like domain-containing proteins | (+) | (#) |
| R119.4 | pqn-59 | this gene is predicted to contain a glutamine/asparagine (Q/N)-rich ('prion') domain | (+) |  |
| R11A8.2 | R11A8.2 | G-patch nucleic acid binding protein | (-) |  |
| R11E3.6 | eor-1 | eor-1 encodes an ortholog of the human BTB/zinc-finger transcription factor PLZF | (-) |  |
| R12B2.4 | him-10 | no description | (+) |  |
| R12B2.5 | mdt-15 | Positive cofactor 2 (PC2), subunit of a multiprotein coactivator of RNA polymerase II | (-) |  |
| R13H4.5 | R13H4.5 | Unnamed protein | (+) |  |
| R144.9 | mdt-11 | Uncharacterized conserved protein | (+) |  |
| R151.2 | R151.2 | Ribose-phosphate pyrophosphokinase | (+) |  |
| R53.3 | egl-43 | Zn-finger (egl-43) | (-) | (###) |
| T01D1.2 | etr-1 | RNA-binding protein CUGBP1/BRUNO | (+) |  |
| T01G9.6 | kin-10 | Casein kinase II, beta subunit | (-) |  |
| T02C12.3 | T02C12.3 | RNA polymerase III transcription factor (TF)IIIC subunit | (-) |  |
| T02D1.3 | sru-15 | Serpentine Receptor, class U) | (+) |  |
| T02E9.2 | grl-7 | GROUND domains (extracellular cysteine-containing domain sometimes associated with HOG domains | (-) | (##) |
| T03D8.2 | T03D8.2 | Mitochondrial/chloroplast ribosomal protein S12 | (+) |  |
| T04A11.7 | sru-20 | Serpentine Receptor, class U | (+) |  |
| T04A8.6 | T04A8.6 | Nucleolar RNA-binding protein NIFK | (-) |  |
| T04B2.1 | T04B2.1 | none available | (-) | (##) |
| T05A10.3 | ttr-14 | Uncharacterized protein with conserved cysteine(TransThyretin-Related family domain) | (-) |  |
| T05A6.1 | cki-1 | Cyclin-dependent kinase inhibitor | (+) |  |
| T05E11.3 | T05E11.3 | Endoplasmic reticulum glucose-regulated protein | (+) |  |
| T05E11.5 | imp-2 | IntraMembrane Protease (IMPAS) family) | (+) |  |
| T05G5.3 | cdk-1 | Protein kinase PCTAIRE and related kinases | (-) |  |
| T05H10.6 | T05H10.6 | Pyruvate dehydrogenase E1, alpha subunit | (-) |  |
| T05H4.14 | gad-1 | Uncharacterized conserved protein, contains WD40 repeat | (+) |  |
| T06D8.8 | rpn-9 | 26S proteasome regulatory complex, subunit RPN9/PSMD13 | (+) |  |
| T06E6.2 | cyb-3 | Cyclin B and related kinase-activating proteins | (-) |  |
| T07A9.2 | ears-2 | Glutamyl-tRNA synthetase | (-) |  |
| T07A9.8 | T07A9.8 | Predicted RNA methylase involved in rRNA processing | (-) |  |
| T07C4.7 | mev-1 | Succinate dehydrogenase, cytochrome b subunit | (-) |  |
| T07E3.6 | pdf-1 | PDF (arthropod Pigment Dispersing Factor) | (+) |  |
| T07H6.4 | T07H6.4 | C-type lectin | (+) |  |
| T08B1.1 | T08B1.1 | Synaptic vesicle transporter SVOP and related transporters | (-) |  |
| T08B2.4 | T08B2.4 | S-M checkpoint control protein CID1 and related nucleotidyltransferases | (+) |  |
| T08B2.8 | T08B2.8 | Predicted mitochondrial ribosomal protein L23 | (+) |  |
| T09A5.11 | ostb-1 | Oligosaccharyltransferase, beta subunit | (+) |  |
| T09A5.5 | T09A5.5 | none available | (-) |  |
| T10B5.5 | cct-7 | Chaperonin complex component, TCP-1 eta subunit (CCT7 | (+) | (#) |
| T10C6.7 | T10C6.7 | This gene encodes a protein containing an F-box | (-) |  |
| T10E10.3 | T10E10.3 | Unnamed protein | (+) |  |
| T10H9.4 | snb-1 | Synaptobrevin/VAMP-like protein | (-) |  |
| T11F8.3 | rme-2 | Low-density lipoprotein receptors containing Ca2+-binding EGF-like domains | (+) |  |
| T11G6.3 | T11G6.3 | Permease of the major facilitator superfamily | (+) |  |
| T13A10.11 | sams-5 | S-adenosylmethionine synthetase | (-) | (#) |
| T13F2.7 | sna-2 | snRNP-binding protein | (-) |  |
| T13H5.5 | T13H5.5 | Mitochondrial ribosomal protein S18b | (-) |  |
| T16G1.5 | T16G1.5 | Predicted small molecule kinase | (+) |  |
| T17E9.2 | nmt-1 | N-myristoyl transferase | (-) |  |
| T19A6.2 | ngp-1 | Nucleolar GTPase | (-) | (#) |
| T19B4.4 | dnj-21 | Molecular chaperone | (-) |  |
| T19D7.3 | lpr-7 | no | (+) |  |
| T19E10.1 | ect-2 | Predicted guanine nucleotide exchange factor | (-) |  |
| T20B12.2 | tbp-1 | TATA-box binding protein | (-) |  |
| T20B12.3 | T20B12.3 | Predicted nucleolar protein involved in ribosome biogenesis | (-) |  |
| T20B5.3 | oga-1 | O-GlcNAc selective N-Acetyl-beta-D-glucosaminidase | (+) |  |
| T20D3.7 | vps-26 | Membrane coat complex Retromer, subunit VPS26 | (+) |  |
| T20G5.2 | cts-1 | Citrate synthase | (+) |  |
| T20H4.5 | T20H4.5 | NADH:ubiquinone oxidoreductase, NDUFS8/23 kDa subunit | (+) |  |
| T21B10.1 | T21B10.1 | Uncharacterized conserved protein | (-) |  |
| T21C9.1 | T21C9.1 | PDZ domain | (+) |  |
| T21C9.12 | scpl-4 | TFIIF-interacting CTD phosphatase, including NLI-interacting factor | (+) |  |
| T22B11.2 | T22B11.2 | Galactosyltransferases | (+) |  |
| T22B11.5 | T22B11.5 | 2-oxoglutarate dehydrogenase, E1 subunit | (-) |  |
| T22B7.4 | T22B7.4 | Histone acetyltransferase SAGA associated factor SGF29 | (+) |  |
| T22D1.10 | ruvb-2 | DNA helicase TIP49, TBP-interacting protein | (+) |  |
| T22D1.4 | T22D1.4 | Oligosaccharyltransferase, alpha subunit | (-) |  |
| T22H9.1 | T22H9.1 | Uncharacterized conserved protein | (+) |  |
| T23B12.2 | T23B12.2 | Mitochondrial/chloroplast ribosomal protein L4 | (-) | (#) |
| T23B12.3 | T23B12.3 | Mitochondrial/chloroplast ribosomal protein S2 | (+) |  |
| T23D8.3 | T23D8.3 | Uncharacterized conserved protein | (+) | (#) |
| T23F6.4 | rbd-1 | RNA-binding protein | (+) |  |
| T23G11.2 | gna-2 | Glucosamine-phosphate N-acetyltransferase | (+) |  |
| T23G11.3 | gld-1 | RNA-binding protein | (-) |  |
| T23G5.1 | rnr-1 | Ribonucleotide reductase, alpha subunit | (-) |  |
| T23H2.5 | rab-10 | GTP-binding protein SEC4, small G protein superfamily | (+) |  |
| T24D1.1 | sqv-5 | sqv-5 encodes a chondroitin synthase that both initiates and elongates chondroitin chains | (+) |  |
| T24D8.5 | nlp-2 | nlp-2 - (Neuropeptide-Like Protein) | (+) |  |
| T24H7.1 | phb-2 | called Band 7 protein/required for mitochondria biogenesis | (-) |  |
| T25B9.10 | T25B9.10 | Inositol polyphosphate 5-phosphatase and related proteins | (+) |  |
| T25D3.2 | T25D3.2 | Uncharacterized conserved protein | (+) |  |
| T26A8.4 | T26A8.4 | Polyadenylation factor I complex, subunit, Yth1 | (+) |  |
| T26C11.6 | ceh-21 | ceh-21 encodes a a ONECUT class CUT homeobox protein | (+) |  |
| T26C11.7 | ceh-39 | CCAAT displacement protein and related homeoproteins | (+) |  |
| T26E3.3 | par-6 | Cell polarity protein PAR6 | (-) |  |
| T26G10.1 | T26G10.1 | ATP-dependent RNA helicase | (-) |  |
| T27C5.5 | srh-132 | Predicted olfactory G-protein coupled receptor | (+) |  |
| T27F6.5 | pars-2 | Prolyl-tRNA synthetase | (+) |  |
| T28A11.16 | T28A11.16 | Predicted secreted cysteine rich protein found only in C.elegans | (+) |  |
| T28A11.2 | T28A11.2 | Predicted secreted cysteine rich protein found only in C.elegans | (+) |  |
| T28D6.6 | T28D6.6 | GTP-binding protein DRG1 | (+) |  |
| VC5.4 | mys-1 | Histone acetyltransferase | (+) |  |
| W01B11.3 | nol-5 | Ribosome biogenesis protein - Nop58p/Nop5p | (-) |  |
| W01B6.9 | ndc-80 | Centromere-associated protein HEC1 | (-) |  |
| W01G7.3 | rpb-11 | RNA polymerase, subunit L | (-) |  |
| W02A2.3 | pqn-74 | Prion-like-(Q/N-rich)-domain-bearing protein | (+) |  |
| W02B12.3 | rsp-1 | Alternative splicing factor SRp55/B52/SRp75 | (+) |  |
| W02D3.9 | unc-37 | Transducin-like enhancer of split protein | (-) |  |
| W02D9.1 | pri-2 | Eukaryotic-type DNA primase, large subunit | (-) |  |
| W02F12.6 | sna-1 | Predicted secreted cysteine rich protein found only in C.elegans | (-) | (#) |
| W02H5.8 | W02H5.8 | Dihydroxyacetone kinase/glycerone kinase | (+) |  |
| W03B1.4 | sars-2 | Seryl-tRNA synthetase | (+) |  |
| W03C9.1 | W03C9.1 | no | (+) |  |
| W03F11.6 | afd-1 | Actin filament-binding protein Afadin | (-) |  |
| W03F9.1 | W03F9.1 | C4-type Zn-finger protein | (-) |  |
| W03G11.2 | W03G11.2 | Cell cycle control protein | (+) |  |
| **W03H9.4** | **cacn-1** | **cacn-1 encodes an ortholog of Drosophila CACTIN and human C19orf29 that is required** | **(-)** | **(#)** |
| W04A8.7 | taf-1 | Transcription initiation factor TFIID | (-) |  |
| W04B5.4 | W04B5.4 | Mitochondrial ribosomal protein L30 | (-) |  |
| W04G5.2 | rab-11.2 | rab-11.2 - (RAB family) | (+) |  |
| W05B10.1 | his-74 | Histones H3 and H4 | (+) |  |
| W05E10.1 | W05E10.1 | Predicted transporter/transmembrane protein | (+) |  |
| W05E10.2 | W05E10.2 | no | (-) |  |
| W05E10.3 | ceh-32 | Transcription factor SIX and related HOX domain proteins | (-) |  |
| W06D12.6 | W06D12.6 | Unnamed protein | (+) |  |
| W06E11.2 | tag-267 | none available | (+) |  |
| W06F12.1 | lit-1 | Nemo-like MAPK-related serine/threonine protein kinase | (-) |  |
| **W07B3.2** | **gei-4** | **Unnamed protein (gei-4 encodes a protein with a coiled-coil domain)** | **(-)** | **(###)** |
| W07E6.2 | W07E6.2 | Notchless-like WD40 repeat-containing protein | (+) |  |
| W08G11.3 | W08G11.3 | no | (+) |  |
| W09C5.1 | W09C5.1 | Uncharacterized conserved protein | (-) | (##) |
| W09C5.2 | unc-59 | Septin family protein | (-) |  |
| W09D10.3 | W09D10.3 | Mitochondrial/chloroplast ribosomal protein L12 | (+) |  |
| W09G12.4 | dsl-1 | Proteins containing Ca2+-binding EGF-like domains | (+) | (#) |
| W10C8.2 | pop-1 | Transcription factor TCF-4 | (-) | (##) |
| W10D5.2 | nduf-7 | NADH-ubiquinone oxidoreductase | (+) |  |
| W10D9.5 | tomm-22 | Translocase of outer mitochondrial membrane complex, subunit TOM22 | (-) |  |
| W10G6.2 | sgk-1 | sgk-1 encodes a serine/threonine protein kinase | (-) |  |
| Y102A5C.9 | fbxa-148 | F-box A protein | (+) |  |
| Y105E8A.17 | ekl-4 | DNA methyltransferase 1-associated protein-1 | (-) |  |
| Y105E8A.23 | Y105E8A.23 | no | (-) |  |
| Y105E8A.6 | unc-95 | Adaptor protein Enigma and related PDZ-LIM proteins (paxillin/unc-95) | (+) |  |
| Y110A7A.1 | hcp-6 | Uncharacterized conserved protein related to condensin complex subunit 1 | (-) | (#) |
| Y110A7A.11 | use-1 | Predicted membrane protein | (-) |  |
| Y110A7A.14 | pas-3 | 20S proteasome, regulatory subunit alpha type PSMA4/PRE9 | (+) |  |
| Y110A7A.17 | mat-1 | DNA-binding cell division cycle control protein | (-) | (#) |
| Y111B2A.11 | epc-1 | Polycomb enhancer protein, EPC (Histone modification) | (-) | (#) |
| Y111B2A.18 | rsp-3 | protein family of nuclear phosphoproteins that are required for constitutive splicing and influence alternative splicing regulation | (+) |  |
| Y113G7B.23 | psa-1 | Chromatin remodeling factor subunit and related transcription factors | (-) | (##) |
| Y116A8A.9 | map-2 | Metallopeptidase | (+) |  |
| Y116A8C.34 | cyn-13 | Cyclophilin-type peptidyl-prolyl cis-trans isomerase | (+) |  |
| Y116A8C.36 | itsn-1 | encodes a homolog of human NCF1, which when mutated leads to chronic granulomatous disease | (+) |  |
| Y119C1B.8 | bet-1 | Transcription initiation factor TFIID, subunit BDF1 and related bromodomain proteins | (-) | (#) |
| Y17D7B.4 | Y17D7B.4 | no | (+) |  |
| Y18D10A.5 | gsk-3 | Glycogen synthase kinase-3 | (+) |  |
| Y2H9A.2 | srd-17 | Serpentine Receptor, class D | (+) |  |
| Y34B4A.7 | Y34B4A.7 | Predicted spermine/spermidine synthase | (-) |  |
| Y34D9A.1 | Y34D9A.1 | Phosphatidylethanolamine binding protein | (+) |  |
| Y37D8A.16 | Y37D8A.16 | none available | (-) |  |
| Y37D8A.9 | mrg-1 | Dosage compensation regulatory complex/histone acetyltransferase complex, subunit MSL-3/MRG15/EAF3 | (+) |  |
| Y37E11AM.3 | Y37E11AM.3 | protein subunit of the endoribonuclease RNAse P, which cleaves tRNA precursors to produce their mature 5' end | (-) | (#) |
| Y37H2C.3 | fbxa-213 | Uncharacterized protein. fbxa-213 - (F-box A protein) | (+) |  |
| Y38H6C.7 | Y38H6C.7 | no | (+) |  |
| Y39A1A.12 | Y39A1A.12 | Origin recognition complex | (-) | (##) |
| Y39B6A.3 | Y39B6A.3 | Fe-S cluster biosynthesis protein | (-) |  |
| Y39B6A.33 | Y39B6A.33 | Cellular protein (glioma tumor suppressor candidate region gene 2 | (-) | (#) |
| Y39B6A.39 | Y39B6A.39 | Mitochondrial ribosomal protein S28 | (-) |  |
| Y39B6A.41 | Y39B6A.41 | no | (+) |  |
| Y39G10AL.3 | cdk-7 | Cdk activating kinase (CAK)/RNA polymerase II transcription initiation | (-) |  |
| Y40B10A.9 | Y40B10A.9 | Phospholipase | (+) |  |
| Y40B1B.5 | eif-3.J | Translation initiation factor eIF3, p35 subunit | (-) |  |
| Y40H7A.10 | Y40H7A.10 | Cysteine proteinase Cathepsin L | (+) |  |
| Y41C4A.9 | Y41C4A.9 | Uncharacterized conserved protein | (+) |  |
| Y41E3.11 | Y41E3.11 | Scaffold/matrix specific factor hnRNP-U/SAF-A, contains SPRY domain | (-) | (##) |
| Y45F10D.9 | sas-6 | sas-6 encodes a protein that contains a coiled-coil region and a novel PISA | (+) | (#) |
| Y45G12C.9 | srd-72 | Chemoreceptor/7TM receptor | (+) |  |
| Y47D3A.26 | smc-3 | none available | (-) | (##) |
| Y47H9C.7 | Y47H9C.7 | Translation initiation factor 2B, beta subunit | (-) | (#) |
| Y48A6B.3 | Y48A6B.3 | Box H/ACA snoRNP component, involved in ribosomal RNA pseudouridinylation | (+) |  |
| Y48A6C.2 | Y48A6C.2 | no | (+) |  |
| Y48B6A.1 | Y48B6A.1 | WD40 repeat nucleolar protein Bop1, involved in ribosome biogenesis | (+) | (#) |
| Y48C3A.7 | mac-1 | Nuclear AAA ATPase (VCP subfamily) | (-) |  |
| Y48G1A.4 | Y48G1A.4 | Nucleolar protein involved in 40S ribosome biogenesis | (-) | (##) |
| Y48G1A.5 | xpo-2 | Nuclear export receptor CSE1/CAS (importin beta superfamily) | (-) | (##) |
| Y50D7A.11 | Y50D7A.11 | none available | (-) |  |
| Y50D7A.4 | Y50D7A.4 | N-terminal acetyltransferase | (-) |  |
| Y50D7A.7 | ads-1 | Alkyl-dihydroxyacetonephosphate synthase | (+) |  |
| Y51A2D.7 | Y51A2D.7 | no | (-) | (##) |
| Y51H1A.3 | Y51H1A.3 | NADH:ubiquinone oxidoreductase, NDUFB8/ASHI subunit | (-) |  |
| Y51H4A.3 | rho-1 | Ras-related small GTPase, Rho type | (-) |  |
| Y52B11A.10 | Y52B11A.10 | Protein involved in high osmolarity signaling pathway | (-) |  |
| Y52B11A.9 | Y52B11A.9 | Protein containing a U1-type Zn-finger and implicated in RNA splicing or processing | (+) |  |
| Y53C12A.1 | wee-1.3 | Cyclin-dependent kinase | (-) | (##) |
| Y53C12B.2 | Y53C12B.2 | Predicted RNA-binding protein | (-) | (#) |
| Y53F4B.13 | Y53F4B.13 | FtsJ-like RNA methyltransferase | (-) |  |
| Y53F4B.20 | Y53F4B.20 | Puromycin-sensitive aminopeptidase and related aminopeptidases | (-) |  |
| Y53F4B.22 | arp-1 | Actin and related proteins | (-) |  |
| Y53G8AL.2 | Y53G8AL.2 | NADH:ubiquinone oxidoreductase, NDUFA9/39kDa subunit | (-) |  |
| Y54E10A.15 | cdt-1 | activity is essential for DNA replication; CDT-1 levels are negatively regulated by a CUL-4-containing ubiquitin ligase complex | (+) |  |
| Y54E10BR.5 | Y54E10BR.5 | Signal peptidase I | (-) |  |
| Y54E5A.4 | npp-4 | Nuclear pore complex, Nup98 component | (+) | (#) |
| Y54G9A.7 | Y54G9A.7 | none available | (+) |  |
| Y54H5A.3 | tag-262 | RNA-binding proteins | (-) |  |
| Y55B1BM.1 | stim-1 | Cell surface glycoprotein STIM, contains SAM domain | (+) |  |
| Y55D9A.1 | efa-6 | Guanine nucleotide exchange factor EFA6 | (-) |  |
| Y55F3BR.1 | Y55F3BR.1 | Putative DEAD-box RNA helicase DDX1 | (-) | (#) |
| Y56A3A.1 | ntl-3 | CCR4-NOT transcriptional regulation complex | (+) |  |
| Y56A3A.18 | Y56A3A.18 | U1-like Zn-finger-containing protein, probabl erole in RNA processing | (-) |  |
| Y56A3A.20 | ccf-1 | mRNA deadenylase subunit | (-) |  |
| Y56A3A.32 | wah-1 | Programmed cell death 8 | (+) |  |
| Y57A10A.27 | Y57A10A.27 | no | (+) |  |
| **Y57G11C.10** | **gdi-1** | **RAB proteins geranylgeranyltransferase component A** | **(-)** |  |
| Y57G11C.12 | nuo-3 | NADH:ubiquinone oxidoreductase | (-) |  |
| Y57G11C.2 | lgc-7 | Acetylcholine receptor | (+) | (#) |
| Y57G11C.31 | Y57G11C.31 | Uncharacterized protein | (-) |  |
| Y59A8B.12 | Y59A8B.12 | no | (-) |  |
| Y60A3A.19 | Y60A3A.19 | Uncharacterized conserved protein | (-) |  |
| Y60A3A.9 | Y60A3A.9 | emp24/gp25L/p24 family of membrane trafficking proteins | (+) |  |
| Y61B8B.1 | sri-70 | Serpentine Receptor, class I | (+) |  |
| Y62E10A.13 | Y62E10A.13 | similarity to Pfam domain PF00702 Hydrolase; haloacid dehalogenase-like hydrolase. This family are structurally different from the alpha/ beta hydrolase family | (+) |  |
| Y62E10A.17 | Y62E10A.17 | Transcription factor AP-2 | (+) |  |
| Y62H9A.3 | Y62H9A.3 | Unnamed protein | (+) |  |
| Y65B4BR.5 | Y65B4BR.5 | Transcription factor containing NAC and TS-N domains | (-) |  |
| Y66D12A.2 |  | Y66D12A.2 has been superseded | (-) |  |
| Y66H1A.2 | dpm-1 | Dolichol-phosphate mannosyltransferase | (-) | (#) |
| Y66H1A.4 | Y66H1A.4 | H/ACA small nucleolar RNP component GAR1 | (+) |  |
| Y6B3B.9 | Y6B3B.9 | contains similarity to Pfam domain | (+) |  |
| **Y70D2A.1** | **Y70D2A.1** | **7 transmembrane receptor** | **(+)** |  |
| Y71F9AM.5 | nxt-1 | RNA export factor NXT1 | (-) |  |
| Y71H10B.1 | Y71H10B.1 | IMP-GMP specific 5'-nucleotidase | (+) |  |
| Y71H2AM.23 | tufm-1 | Mitochondrial translation elongation factor Tu | (-) |  |
| Y71H2B.3 | ppfr-4 | Protein phosphatase 2A-associated protein | (+) |  |
| Y71H2B.6 | mdt-19 | Uncharacterized conserved protein | (+) |  |
| letm-1 | lag-2 | lag-2/ligand for Notch Receptor | (+) |  |
| Y73F8A.24 | Y73F8A.24 | RNA polymerase II transcription initiation/nucleotide excision repair factor | (-) | (#) |
| Y74C10AR.1 | eif-3.I | Translation initiation factor 3 | (+) |  |
| Y75B8A.30 | pph-4.1 | Serine/threonine specific protein phosphatase involved in glycogen accumulation | (+) |  |
| Y75B8A.7 | Y75B8A.7 | U3 small nucleolar ribonucleoprotein | (-) |  |
| Y76B12C.7 | cpsf-1 | mRNA cleavage and polyadenylation factor II complex, subunit CFT1 | (-) | (##) |
| Y77E11A.5 | nhr-41 | Nuclear Hormone Receptor family | (+) |  |
| Y77E11A.7 | Y77E11A.7 | no | (-) | (#) |
| Y77E11A.9 | clec-171 | Cysteine rich domain (CW domain) probably distantly related to the C-type lectin | (-) | (#) |
| Y80D3A.4 | nhr-243 | Hormone receptors | (+) |  |
| ZC196.3 | ZC196.3 | Unnamed protein | (+) |  |
| ZC376.6 | ZC376.6 | no | (-) | (##) |
| ZC395.3 | toc-1 | Putative Zn2+ transporter MSC2 | (+) |  |
| ZC434.4 | ZC434.4 | rRNA processing protein RRP7 | (-) |  |
| ZC477.9 | deb-1 | Alpha-catenin | (-) |  |
| ZC513.4 | vars-1 | Valyl-tRNA synthetase | (+) |  |
| ZC64.3 | ceh-18 | Transcription factor OCT-1, contains POU and HOX domains | (+) |  |
| ZK1127.4 | ZK1127.4 | Isoamyl acetate-hydrolyzing esterase and related enzymes | (-) |  |
| ZK1127.5 | ZK1127.5 | RNA 3'-terminal phosphate cyclase | (-) |  |
| ZK1127.7 | cin-4 | cin-4 - (Chromosome Instability) | (-) |  |
| ZK1127.9 | tcer-1 | Transcription factor CA150 | (-) |  |
| ZK1128.2 | mett-10 | Predicted DNA methylase | (+) |  |
| ZK154.7 | adm-4 | Tumor necrosis factor-alpha-converting enzyme | (-) |  |
| ZK262.10 | srj-26 | 7-transmembrane olfactory receptor | (+) |  |
| ZK262.4 | ZK262.4 | WSN domain | (+) |  |
| ZK265.6 | ZK265.6 | Uncharacterized conserved protein | (+) |  |
| ZK287.5 | rbx-1 | SCF ubiquitin ligase, Rbx1 component | (+) |  |
| ZK430.1 | toe-1 | Uncharacterized conserved protein | (-) |  |
| ZK430.7 | ZK430.7 | Sof1-like rRNA processing protein | (-) |  |
| ZK455.4 | asm-2 | Acid sphingomyelinase and PHM5 phosphate metabolism protein | (+) |  |
| ZK512.2 | ZK512.2 | ATP-dependent RNA helicase | (+) |  |
| ZK546.14 | ZK546.14 | Uncharacterized conserved protein | (-) |  |
| ZK616.6 | ZK616.6 | Predicted membrane protein | (-) | (#) |
| ZK632.13 | lin-52 | Uncharacterized conserved protein | (-) |  |
| ZK637.7 | lin-9 | lin-9 - (abnormal cell LINeage) | (+) |  |
| ZK637.8 | unc-32 | Vacuolar H+-ATPase V0 sector, subunit a | (-) |  |
| ZK673.7 | tnc-2 | Calmodulin and related proteins | (-) |  |
| ZK686.3 | ZK686.3 | Oligosaccharyltransferase, gamma subunit | (-) |  |
| ZK721.1 | tag-130 | tag-130 encodes a predicted transmembrane protein; | (+) |  |
| ZK792.3 | inx-9 | Innexin-type channels | (+) |  |
| ZK792.6 | let-60 | Ras-related GTPase | (+) |  |
| ZK795.3 | ZK795.3 | U3 small nucleolar ribonucleoprotein | (+) |  |
| ZK809.3 | ZK809.3 | NADH:ubiquinone oxidoreductase, NDUFB6/B17 subunit | (-) |  |
| ZK829.4 | ZK829.4 | Glutamate/leucine/phenylalanine/valine dehydrogenases | (+) |  |
| ZK829.8 | srj-1 | 7-transmembrane olfactory receptor | (-) |  |
| ZK856.10 | ZK856.10 | DNA-directed RNA polymerase subunit E | (-) |  |
| ZK856.11 | ZK856.11 | Predicted translation initiation factor related to eIF-1A | (+) |  |
| ZK856.6 | ZK856.6 | Unnamed protein | (-) |  |
| ZK867.1 | syd-9 | Zn-finger | (-) |  |
| ZK896.3 | ZK896.3 | none available | (+) |  |
| ZK899.4 | tba-8 | Alpha tubulin | (+) |  |
| ZK970.3 | mdt-22 | none available | (-) |  |

Bolded genes are hits also included in Table 1.

^a^Egg production was scored relative to the number of eggs laid by *unc-40(e271)* worms after 70 hours at 20°.

(+): Indicates moderate reduction of laid eggs

(++): Indicates 50% reduction of laid eggs

(-): Indicates no eggs laid

^b^Protruding vulvals were scored as a percentage of worms in each well that exhibited this phenotype after 70 hours at 20°.

(#): Indicates 10% of worms had protruding vulvas

(##): Indicates 20% of worms had protruding vulvas

(###): Indicates more than 50% of worms had protruding vulvas
