# Supplementary material for: A Sensitized Screen for Genes Promoting Invadopodia Function In Vivo: CDC-42 and Rab GDI-1 Direct Distinct Aspects of Invadopodia Formation
Source: PLoS Genet. 2016 Jan 14;12(1):e1005786. doi: 10.1371/journal.pgen.1005786 (PMC4713207; doi:10.1371/journal.pgen.1005786)
Supplement: S2 Table — (DOCX) [file pgen.1005786.s002.docx]

**S2 Table. Genes screened for AC invasion defects in *unc-40(e271).***

| **Sequence ID** | **Gene name** | **GO Term** | **Full invasion^±^** | **Partial invasion** | **No invasion** | **n=** |
| --- | --- | --- | --- | --- | --- | --- |
|  | L4440 | Control | 29% | 17% | 54% | 150 |
| ZC477.9 | *deb-1* | Cell adhesion | 22% | 18% | 60% | 50 |
| **C06C3.1** | ***mel-11***** | **Cell-cell adherens junction** | **9%** | **24%** | **67%** | **150** |
| B0222.6 | *col-144* | Extracellular space | 14% | 16% | 70% | 50 |
| C31B8.12 | *C31B8.12* | Extracellular space | 28% | 20% | 52% | 50 |
| **C37C3.6** | ***mig-6***** | **Extracellular space** | **4%** | **7%** | **89%** | **150** |
| E03G2.4 | *col-186* | Extracellular space | 28% | 14% | 58% | 50 |
| F19C7.7 | *col-110* | Extracellular space | 26% | 20% | 54% | 50 |
| F36A4.10 | *col-34* | Extracellular space | 24% | 8% | 68% | 50 |
| F36A4.6 | *col-33* | Extracellular space | 26% | 16% | 58% | 50 |
| F43G9.10 | *mfap-1* | Extracellular space | 24% | 16% | 60% | 50 |
| F58B3.9 | *ttr-50* | Extracellular space | 30% | 16% | 54% | 50 |
| H14N18.3 | *ttr-47* | Extracellular space | 22% | 20% | 58% | 50 |
| K07C11.3 | *K07C11.3* | Extracellular space | 26% | 12% | 62% | 50 |
| T19D7.3 | *lpr-7* | Extracellular space | 21% | 19% | 60% | 150 |
| W02A2.3 | *pqn-74* | Extracellular space | 22% | 12% | 66% | 50 |
| ZK455.4 | *asm-2* | Extracellular space | 24% | 8% | 68% | 50 |
| F18G5.3 | *gpa-12* | G-protein coupled receptor signaling | 18% | 14% | 68% | 50 |
| M01D7.7 | *egl-30* | G-protein coupled receptor signaling | 32% | 14% | 54% | 50 |
| **R06A10.2** | ***gsa-1***** | **G-protein coupled receptor signaling** | **11%** | **12%** | **77%** | **150** |
| C14A11.3 | *cgef-1* | GTPase regulator activity | 18% | 14% | 68% | 50 |
| **C38C10.4** | ***gpr-2***** | **GTPase regulator activity** | **10%** | **18%** | **72%** | **150** |
| **F22B7.13** | ***gpr-1***** | **GTPase regulator activity** | **9%** | **13%** | **77%** | **150** |
| K08E3.6 | *cyk-4* | GTPase regulator activity | 18% | 14% | 68% | 50 |
| T19E10.1 | *ect-2* | GTPase regulator activity | 20% | 10% | 70% | 50 |
| Y55D9A.1 | *efa-6* | GTPase regulator activity | 26% | 18% | 56% | 50 |
| **Y57G11C.10** | ***gdi-1***** | **GTPase regulator activity** | **5%** | **15%** | **80%** | **150** |
| F21C3.5 | *pfd-6* | Inductive cell migration | 24% | 22% | 54% | 50 |
| **F36H1.4** | ***lin-3*** | **Integral compenent of membrane; Extracellular space** |  |  |  | **0** |
| C01B4.9 | *C01B4.9* | Integral component of membrane | 22% | 12% | 66% | 50 |
| C01G5.9 | *C01G5.9* | Integral component of membrane | 28% | 12% | 60% | 50 |
| C03C10.3 | *rnr-2* | Integral component of membrane | 20% | 18% | 62% | 50 |
| C04F5.1 | *sid-1* | Integral component of membrane | 16% | 12% | 72% | 50 |
| C05E11.3 | *C05E11.3* | Integral component of membrane | 30% | 10% | 60% | 50 |
| C05G5.1 | *C05G5.1* | Integral component of membrane | 32% | 14% | 54% | 50 |
| C06E7.1 | *sams-3* | Integral component of membrane | 16% | 14% | 70% | 50 |
| C08B11.4 | *nrf-6* | Integral component of membrane | 20% | 20% | 60% | 50 |
| C08H9.12 | *C08H9.12* | Integral component of membrane | 18% | 18% | 64% | 50 |
| C09G12.8 | *ced-10* | Integral component of membrane | 22% | 22% | 56% | 50 |
| C17E4.9 | *nkb-1* | Integral component of membrane | 16% | 14% | 70% | 50 |
| C23H3.2 | *C23H3.2* | Integral component of membrane | 30% | 14% | 56% | 50 |
| C24D10.4 | *C24D10.4* | Integral component of membrane | 18% | 16% | 66% | 50 |
| **C29F9.7** | ***pat-4**** | **Integral component of membrane** | **12%** | **17%** | **71%** | **150** |
| C30H6.2 | *tag-141* | Integral component of membrane | 24% | 14% | 62% | 50 |
| C31H2.2 | *dpy-8* | Integral component of membrane | 20% | 18% | 62% | 50 |
| C33A12.14 | *sru-1* | Integral component of membrane | 16% | 16% | 68% | 50 |
| C34B2.10 | *C34B2.10* | Integral component of membrane | 20% | 14% | 66% | 50 |
| C34B2.8 | *C34B2.8* | Integral component of membrane | 29% | 8% | 63% | 49 |
| C34D4.8 | *str-48* | Integral component of membrane | 26% | 18% | 56% | 50 |
| C34F6.7 | *C34F6.7* | Integral component of membrane | 20% | 14% | 66% | 50 |
| C44C10.3 | *C44C10.3* | Integral component of membrane | 26% | 20% | 54% | 50 |
| C47A4.2 | *C47A4.2* | Integral component of membrane | 24% | 16% | 60% | 50 |
| C47E12.2 | *C47E12.2* | Integral component of membrane | 20% | 18% | 62% | 50 |
| D1009.3 | *D1009.3* | Integral component of membrane | 30% | 16% | 54% | 50 |
| D2024.3 | *elo-3* | Integral component of membrane | 30% | 14% | 56% | 50 |
| E01A2.7 | *E01A2.7* | Integral component of membrane | 20% | 14% | 66% | 50 |
| F01E11.1 | *ugt-57* | Integral component of membrane | 24% | 12% | 64% | 50 |
| F01G4.6 | *F01G4.6* | Integral component of membrane | 28% | 14% | 58% | 50 |
| F02C12.5 | *cyp-13B1* | Integral component of membrane | 28% | 16% | 56% | 50 |
| **F09A5.4** | ***F09A5.4***** | **Integral component of membrane** | **9%** | **19%** | **71%** | **150** |
| F09C8.1 | *F09C8.1* | Integral component of membrane | 28% | 22% | 50% | 50 |
| F09F9.4 | *F09F9.4* | Integral component of membrane | 26% | 10% | 64% | 50 |
| F10D2.9 | *fat-7* | Integral component of membrane | 20% | 12% | 68% | 50 |
| F10D7.2 | *F10D7.2* | Integral component of membrane | 22% | 12% | 66% | 50 |
| F15A4.5 | *F15A4.5* | Integral component of membrane | 28% | 18% | 54% | 50 |
| F23B2.3 | *F23B2.3* | Integral component of membrane | 20% | 16% | 64% | 50 |
| F23H12.2 | *tomm-20* | Integral component of membrane | 18% | 12% | 70% | 50 |
| **F25E2.1** | ***F25E2.1***** | **Integral component of membrane** | **10%** | **19%** | **71%** | **150** |
| F27D9.6 | *dhs-29* | Integral component of membrane | 26% | 16% | 58% | 50 |
| F31F6.6 | *nac-1* | Integral component of membrane | 24% | 12% | 64% | 50 |
| F32D8.5 | *F32D8.5* | Integral component of membrane | 28% | 10% | 62% | 50 |
| F40C5.1 | *F40C5.1* | Integral component of membrane | 24% | 22% | 54% | 50 |
| F41B5.2 | *cyp-33C7* | Integral component of membrane | 20% | 20% | 60% | 50 |
| F41C3.4 | *F41C3.4* | Integral component of membrane | 20% | 20% | 60% | 50 |
| F42G8.11 | *sph-1* | Integral component of membrane | 14% | 24% | 62% | 50 |
| F44A2.2 | *F44A2.2* | Integral component of membrane | 20% | 10% | 70% | 50 |
| F44G4.2 | *F44G4.2* | Integral component of membrane | 20% | 26% | 54% | 50 |
| F45E10.2 | *F45E10.2* | Integral component of membrane | 28% | 10% | 62% | 50 |
| F52E4.6 | *wrt-2* | Integral component of membrane | 30% | 12% | 58% | 50 |
| F54C9.2 | *stc-1* | Integral component of membrane | 26% | 20% | 54% | 50 |
| F55D10.3 | *glit-1* | Integral component of membrane | 20% | 16% | 64% | 50 |
| F58B3.5 | *mars-1* | Integral component of membrane | 32% | 22% | 46% | 50 |
| F58G11.1 | *letm-1* | Integral component of membrane | 18% | 14% | 68% | 50 |
| F58G6.4 | *acc-1* | Integral component of membrane | 34% | 10% | 56% | 50 |
| F59A2.1 | *npp-9* | Integral component of membrane | 20% | 12% | 68% | 50 |
| F59F5.1 | *F59F5.1* | Integral component of membrane | 14% | 16% | 70% | 50 |
| K01C8.9 | *nst-1* | Integral component of membrane | 20% | 8% | 72% | 50 |
| K02B2.4 | *inx-7* | Integral component of membrane | 26% | 12% | 62% | 50 |
| K03A1.2 | *K03A1.2* | Integral component of membrane | 28% | 10% | 62% | 50 |
| K08B5.1 | *K08B5.1* | Integral component of membrane | 24% | 16% | 60% | 50 |
| K09E10.2 | *oac-58* | Integral component of membrane | 22% | 12% | 66% | 50 |
| K09H9.6 | *lpd-6* | Integral component of membrane | 18% | 20% | 62% | 50 |
| K12B6.3 | *fil-2* | Integral component of membrane | 20% | 16% | 64% | 50 |
| K12B6.4 | *K12B6.4* | Integral component of membrane | 28% | 14% | 58% | 50 |
| M03F4.6 | *zen-4* | Integral component of membrane | 28% | 10% | 62% | 50 |
| R07E4.4 | *mig-23* | Integral component of membrane | 20% | 18% | 62% | 50 |
| **R07G3.1** | ***cdc-42***** | **Integral component of membrane** | **7%** | **11%** | **83%** | **150** |
| R08C7.2 | *R08C7.2* | Integral component of membrane | 28% | 12% | 60% | 50 |
| T02D1.3 | *sru-15* | Integral component of membrane | 24% | 14% | 62% | 50 |
| T05E11.5 | *imp-2* | Integral component of membrane | 20% | 22% | 58% | 50 |
| T07C4.7 | *mev-1* | Integral component of membrane | 22% | 20% | 58% | 50 |
| T07E3.6 | *pdf-1* | Integral component of membrane | 22% | 18% | 60% | 50 |
| T09A5.11 | *ostb-1* | Integral component of membrane | 22% | 12% | 66% | 50 |
| T10H9.4 | *snb-1* | Integral component of membrane | 22% | 16% | 62% | 50 |
| T11F8.3 | *rme-2* | Integral component of membrane | 22% | 18% | 60% | 50 |
| T19B4.4 | *dnj-21* | Integral component of membrane | 22% | 8% | 70% | 50 |
| T21C9.1 | *T21C9.1* | Integral component of membrane | 26% | 12% | 62% | 50 |
| T21C9.12 | *scpl-4* | Integral component of membrane | 20% | 20% | 60% | 50 |
| T22D1.4 | *T22D1.4* | Integral component of membrane | 28% | 18% | 54% | 50 |
| T24D1.1 | *sqv-5* | Integral component of membrane | 18% | 14% | 68% | 50 |
| T24H7.1 | *phb-2* | Integral component of membrane | 21% | 14% | 65% | 150 |
| T27C5.5 | *srh-132* | Integral component of membrane | 20% | 14% | 66% | 50 |
| T28D6.6 | *T28D6.6* | Integral component of membrane | 28% | 12% | 60% | 50 |
| W03C9.1 | *W03C9.1* | Integral component of membrane | 22% | 16% | 62% | 50 |
| W03G11.2 | *W03G11.2* | Integral component of membrane | 22% | 10% | 68% | 50 |
| W05E10.1 | *W05E10.1* | Integral component of membrane | 32% | 10% | 58% | 50 |
| W05E10.2 | *W05E10.2* | Integral component of membrane | 20% | 14% | 66% | 50 |
| **W07B3.2** | ***gei-4**** | **Integral component of membrane** | **6%** | **8%** | **86%** | **50** |
| Y110A7A.11 | *use-1* | Integral component of membrane | 34% | 14% | 52% | 50 |
| Y2H9A.2 | *srd-17* | Integral component of membrane | 26% | 18% | 56% | 50 |
| Y34B4A.7 | *Y34B4A.7* | Integral component of membrane | 34% | 10% | 56% | 50 |
| Y37D8A.16 | *Y37D8A.16* | Integral component of membrane | 26% | 16% | 58% | 50 |
| Y39B6A.41 | *Y39B6A.41* | Integral component of membrane | 22% | 24% | 54% | 50 |
| Y40B10A.9 | *Y40B10A.9* | Integral component of membrane | 24% | 16% | 60% | 50 |
| Y45G12C.9 | *srd-72* | Integral component of membrane | 22% | 10% | 68% | 50 |
| Y53C12A.1 | *wee-1.3* | Integral component of membrane | 32% | 16% | 52% | 50 |
| Y54E10BR.5 | *Y54E10BR.5* | Integral component of membrane | 24% | 14% | 62% | 50 |
| Y55B1BM.1 | *stim-1* | Integral component of membrane | 28% | 18% | 54% | 50 |
| Y56A3A.32 | *wah-1* | Integral component of membrane | 22% | 14% | 64% | 50 |
| Y57G11C.2 | *lgc-7* | Integral component of membrane | 24% | 14% | 62% | 50 |
| Y57G11C.31 | *Y57G11C.31* | Integral component of membrane | 26% | 14% | 60% | 50 |
| Y60A3A.19 | *Y60A3A.19* | Integral component of membrane | 24% | 18% | 58% | 50 |
| Y60A3A.9 | *Y60A3A.9* | Integral component of membrane | 26% | 24% | 50% | 50 |
| Y61B8B.1 | *sri-70* | Integral component of membrane | 22% | 18% | 60% | 50 |
| Y62E10A.13 | *Y62E10A.13* | Integral component of membrane | 28% | 14% | 58% | 50 |
| Y73C8B.4 | *lag-2* | Integral component of membrane | 20% | 20% | 60% | 50 |
| ZC395.3 | *toc-1* | Integral component of membrane | 22% | 22% | 56% | 50 |
| ZK154.7 | *adm-4* | Integral component of membrane | 28% | 14% | 58% | 50 |
| ZK262.10 | *srj-26* | Integral component of membrane | 24% | 16% | 60% | 50 |
| ZK616.6 | *ZK616.6* | Integral component of membrane | 22% | 18% | 60% | 50 |
| ZK637.8 | *unc-32* | Integral component of membrane | 22% | 20% | 58% | 50 |
| ZK686.3 | *ZK686.3* | Integral component of membrane | 22% | 18% | 60% | 50 |
| ZK792.3 | *inx-9* | Integral component of membrane | 24% | 16% | 60% | 50 |
| ZK829.8 | *srj-1* | Integral component of membrane | 18% | 14% | 68% | 50 |
| F46C8.6 | *dpy-7* | Integral component of membrane; Extracellular space | 18% | 22% | 60% | 50 |
| C35A5.7 | *C35A5.7* | Integral component of membrane; GPCR | 29% | 8% | 63% | 49 |
| F42C5.2 | *F42C5.2* | Integral component of membrane; GPCR | 24% | 14% | 62% | 50 |
| K06C4.6 | *dmo-1* | Integral component of membrane; GPCR | 22% | 14% | 64% | 50 |
| **Y70D2A.1** | ***Y70D2A.1**** | **Integral component of membrane; GPCR** | **12%** | **20%** | **68%** | **150** |
| C01G8.5 | *erm-1* | Morphogensis of an epithelium | 24% | 10% | 66% | 50 |
| D2013.6 | *D2013.6* | Receptor mediated endocytosis | 20% | 12% | 68% | 50 |

^±^Full invasion, partial invasion, and no invasion were defined by the degree to which the BM beneath the AC was cleared, as previously described (Sherwood and Sternberg 2003).

Bolded genes are significant hits also included in Table 1.

*Compared with *unc-40(e271)*; p<0.001

**Compared with *unc-40(e271)*; p<0.0001

Sherwood, D. R. and P. W. Sternberg (2003). "Anchor cell invasion into the vulval epithelium in C. elegans." Dev Cell **5**(1): 21-31.
