# Supplementary material for: A Sensitized Screen for Genes Promoting Invadopodia Function In Vivo: CDC-42 and Rab GDI-1 Direct Distinct Aspects of Invadopodia Formation
Source: PLoS Genet. 2016 Jan 14;12(1):e1005786. doi: 10.1371/journal.pgen.1005786 (PMC4713207; doi:10.1371/journal.pgen.1005786)
Supplement: S3 Table — (DOCX) [file pgen.1005786.s003.docx]

**S3 Table. List of transgenes created and primers used.**

| ***qyIs204[wsp-1>GFP]*** | wsp-1> F (outer) | GACCTCCCAGTCGCTTCTTAATC |
| --- | --- | --- |
|  | wsp-1> F (inner) | TTTATTCCTCTCGGGTTTCGCTC |
|  | wsp-1> R | CTGTATGTGTATGTGATTGTGGTG |
|  | GFP F | CACCACAATCACATACACATACAGAT |
|  | GFP R (outer) | ACCTTCCACTGAGCCTCAAAC |
|  | GFP R (inner) | CCCATAGACACTACTCCACTTTC |
| ***qyIs212[cdh-3>GFP::wsp-1]*** | cdh-3>GFP F (outer) | CAGCTATGACCATGATTACGC |
|  | cdh-3>GFP F (inner) | CACTCACAACGATGGATACGC |
|  | cdh-3>GFP R | GATATACCGACATTTTGTATAGTTCATCCATG |
|  | WSP-1 F (inner) | TTTATTCCTCTCGGGTTTCGCTC |
|  | WSP-1 R (inner) | CACGGAATAGGCACTGATTTG |
|  | WSP-1 R (outer) | GTTTGTTGGTATTTCGGGTGTGTG |
| ***qyIs412[cdh-3>GFP::GBDwsp-1]*** | cdh-3> F (outer) | CAGCTATGACCATGATTACGC |
|  | cdh-3> F (inner) | CACTCACAACGATGGATACGC |
|  | cdh-3> R | TTTTTCTGAGCTCGGTACCCTC |
|  | GFP:GBDwsp-1 F | GAGGGTACCGAGCTCAGAAAAAATGAGTAAAGGAGAAGAACT |
|  | GFP:GBDwsp-1 R (outer) | GAGAAAGCTCAACAAAGTGATG |
|  | GFP:GBDwsp-1 R (inner) | TGTGAACCTCATCTTCAGGTAATA |
| ***qyEx507[cdh-3>GFP::gdi-1]*** | cdh-3>GFP F (outer) | TGTAAAACGACGGCCAGT |
|  | cdh-3>GFP F (inner) | AAGGTACCCTAGAGCATGATGTCCTTAC |
|  | cdh-3>GFP R | TTTGTATAGTTCATCCATGCCATGTG |
|  | GDI-1 F | GCATGGATGAACTATACAAAATGGATGAGGAATACGAT |
|  | GDI-1 R (outer) | TATATGCTTATTCTTCTCCAAT |
|  | GDI-1 R (inner) | GATCTCCCTTCATCAAATGTAA |
| ***qyEx515[gdi-1>GFP]*** | gdi-1> F (outer) | ATACCAGCGTCGAGGATTTCTGTC |
|  | gdi-1> F (inner) | CAGAGGCCAGGAATGCAGAATAA |
|  | gdi-1> R | TTCGAGCGAGTTTTTCGGGGAA |
|  | GFP F | CCCCGAAAAACTCGCTCGAAATGAGTAAAGGAGAAGAACTTT |
|  | GFP R (outer) | AAGGGCCCGTACGGCCGACTAGTAGG |
|  | GFP R (inner) | AAACAGTTATGTTTGGTATATTGGG |
| ***qyEx533[cdh3>GFP::Cbgdi-1]*** | cdh-3>GFP F (outer) | CAGCTATGACCATGATTACGC |
|  | cdh-3>GFP F (inner) | CACTCACAACGATGGATACGC |
|  | cdh-3>GFP R | GATGGCATCGTATTCCTCATCCATTTTGTATAGTTCATCCATGCCATG |
|  | Cbrgdi-1 F | ATGGATGAGGAATACGATGCC |
|  | Cbrgdi-1 R (outer) | CTAAATATCGCCCCCACGTG |
|  | Cbrgdi-1 R (inner) | TGATACTCTCCCGCCTTGTG |
