# Supplementary figures and images for: A Sensitized Screen for Genes Promoting Invadopodia Function In Vivo: CDC-42 and Rab GDI-1 Direct Distinct Aspects of Invadopodia Formation
Source: PLoS Genet. 2016 Jan 14;12(1):e1005786. doi: 10.1371/journal.pgen.1005786 (PMC4713207; doi:10.1371/journal.pgen.1005786)

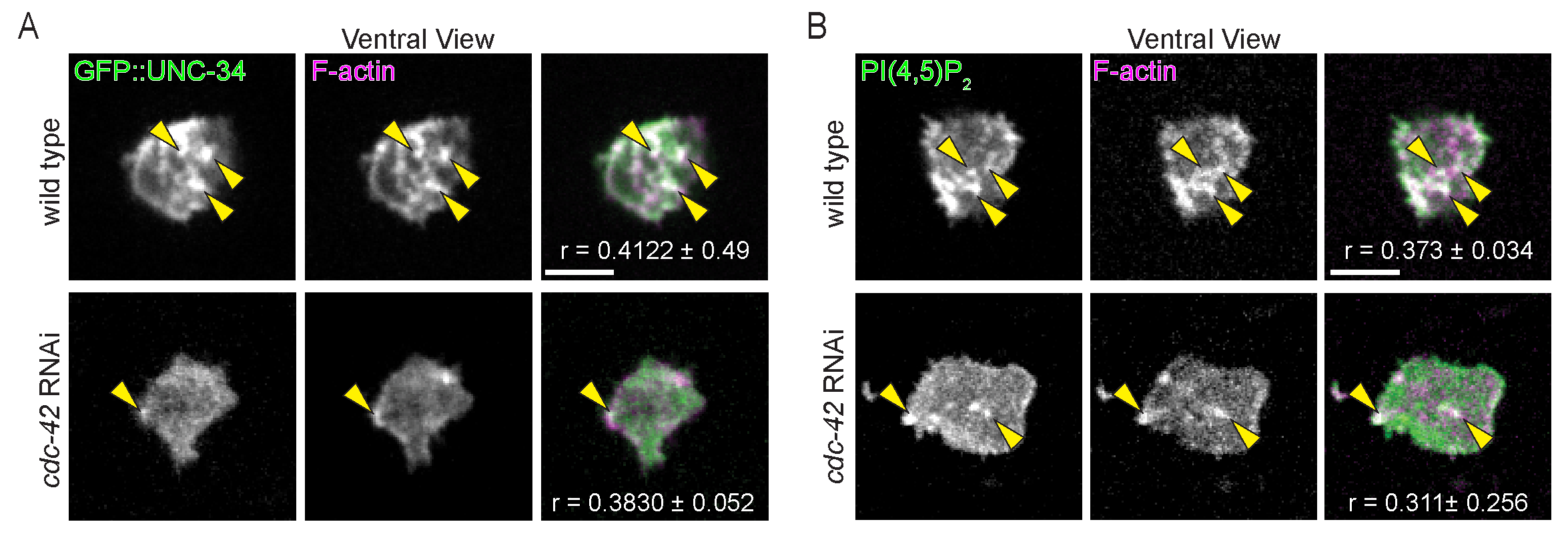

Supplement: S1 Fig — (A, B) Ventral views showing the composition of invadopodia. Top panels: the invadopodial markers GFP::UNC-34 (A; cdh3>GFP::unc-34) and PI(4,5)P2 (B, cdh3> mCherry::PLCδPH) colocalize with F-actin (cdh3>mCherry::moeABD; arrowheads; overlaid text report Pearson’s colocalization coefficients, r). Bottom panels: Knockdown of cdc-42 by RNAi reduces the number of invadopodia, but GFP::UNC-34 (A) and PI(4,5)P2 (B) still colocalize with F-actin (arrowheads). The correlation coefficients are not different between wild type animals and animals treated with cdc-42 RNAi (n > 10 animals for each condition; p = 0.56 (GFP::UNC-34) and 0.26 (PI(4,5)P2, Student’s t-tests). Scale bar, 5 μm. (TIF) [file pgen.1005786.s004.tif]

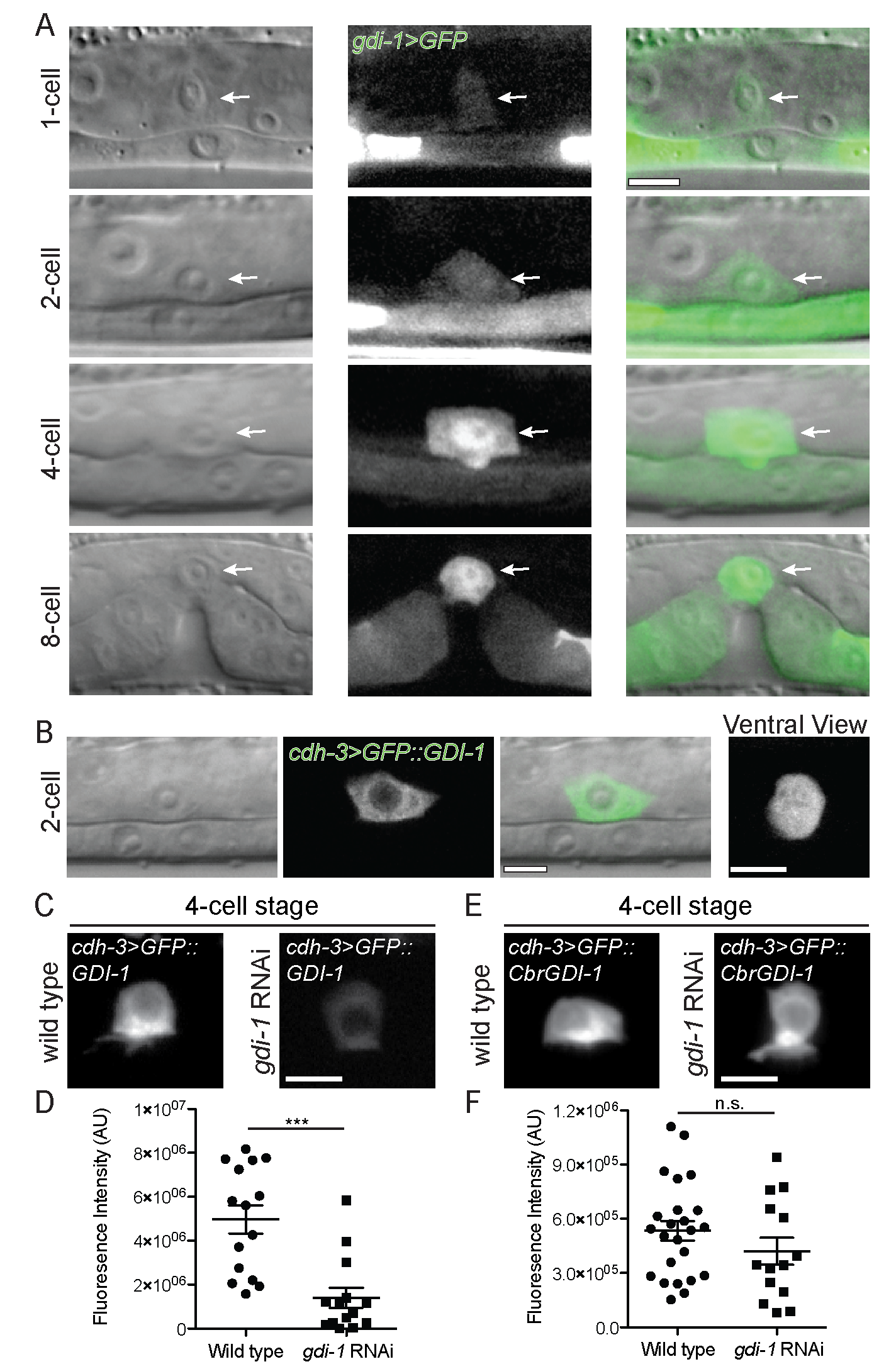

Supplement: S2 Fig — (A) A transcriptional reporter (gdi-1>GFP) revealed gdi-1 expression in the AC and vulval precursor cells throughout invasion. Expression of gdi-1 is specifically upregulated in the AC. (B) An AC-specific full-length fusion of GFP to GDI-1 (cdh3>GFP::gdi-1) showed cytosolic distribution with no specific subcellular enrichment. (C, D) The gdi-1 RNAi construct reduced levels of an AC expressed GDI-1 reporter (cdh3>GFP::gdi-1; n = 15 wild type and 14 gdi-1 RNAi treated animals; *** p < 0.0001, Student’ t-test). (E, F) The gdi-1 RNAi construct from C. elegans did not alter the fluorescence intensity of a GDI-1 reporter made from the related nematode C. briggsae (cdh3>GFP::Cbrgdi-1; n = 15 wild type and 13 gdi-1 RNAi treated animals; p = 0.586, Student’s t-test; n.s = not significant). AU = arbitrary units; scale bar, 5 μm. (TIF) [file pgen.1005786.s005.tif]

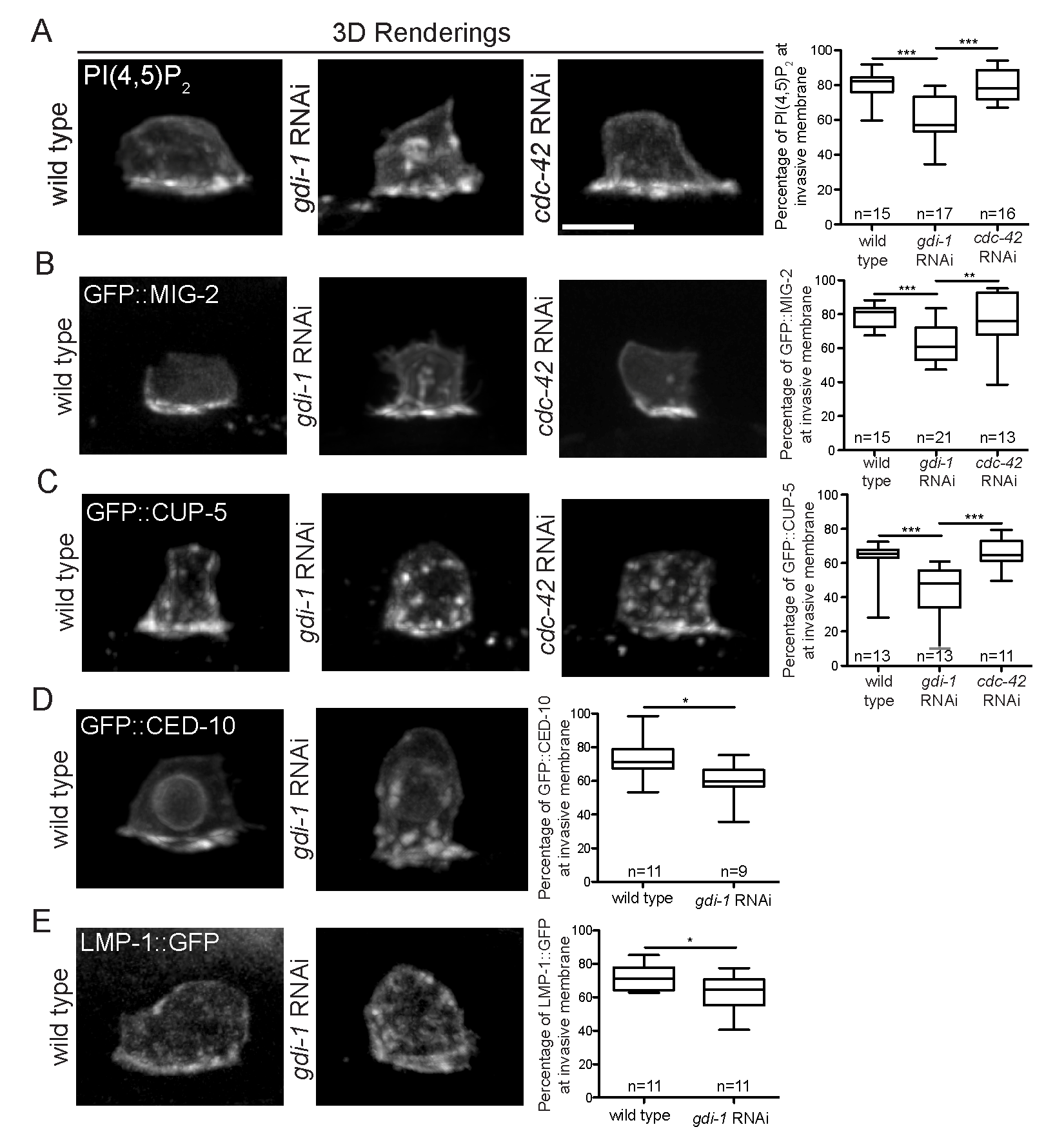

Supplement: S3 Fig — (A-E) 3D renderings showing the distribution of PI(4,5)P2 (A, cdh3> mCherry::PLCδPH), GFP::MIG-2 (B, cdh3>GFP::MIG-2), GFP::CUP-5 (C, cdh3>GFP::cup-5), GFP::CED-10 (D, cdh3>GFP::ced-10), and LMP-1::GFP (E, cdh3>lmp-1::GFP). RNAi mediated knockdown of gdi-1 (middle panels) resulted in mis-trafficking of the invadopodial membrane components PI(4,5)2, GFP::MIG-2, and GFP::CED-10, as well as GFP::CUP-5 and LMP-1::GFP (which are found both in the invadopodial membrane and the endolysosome) relative to wild type (left panels). RNAi targeting of cdc-42 did not affect the distribution of PI(4,5)P2, GFP::MIG-2, or GFP::CUP-5 (right panels). Box plots (line shows median, boxes cover the interquartile range, and bars show minimum and maximum) display the percentage of the total fluorescent signal at or near the basal invasive cell membrane of the AC. For all conditions a minimum of 9 animals were analyzed (n is noted on each graph). In (A-C) comparisons were made using Tukey’s multiple comparisons tests, ** p < 0.01, *** p < 0.001. In (D-E) comparisons were made using a Student’s t-test, * p < 0.05. Scale bar, 5 μm. (TIF) [file pgen.1005786.s006.tif]
